# SLC14A1 prevents oncometabolite accumulation and recruits HDAC1 to transrepress oncometabolite genes in urothelial carcinoma

**DOI:** 10.7150/thno.51655

**Published:** 2020-09-23

**Authors:** Ti-Chun Chan, Wen-Jeng Wu, Wei-Ming Li, Meng-Shin Shiao, Yow-Ling Shiue, Chien-Feng Li

**Affiliations:** 1 Department of Medical Research, Chi Mei Medical Center, Tainan, Taiwan; 2 Institute of Biomedical Sciences, National Sun Yat-sen University, Kaohsiung, Taiwan; 3 National Institute of Cancer Research, National Health Research Institutes, Tainan, Taiwan; 4 Graduate Institute of Clinical Medicine, College of Medicine, Kaohsiung Medical University, Kaohsiung, Taiwan; 5 Department of Urology, Kaohsiung Medical University Hospital, Kaohsiung, Taiwan; 6 Department of Urology, School of Medicine, College of Medicine, Kaohsiung Medical University, Kaohsiung, Taiwan; 7 Cohort Research Center, Kaohsiung Medical University, Kaohsiung, Taiwan, Kaohsiung, Taiwan; 8 Center for Infectious Disease and Cancer Research, Kaohsiung Medical University, Kaohsiung, Taiwan; 9 Center for Stem Cell Research, Kaohsiung Medical University, Kaohsiung, Taiwan; 10 Department of Urology, Ministry of Health and Welfare Pingtung Hospital, Pingtung, Taiwan; 11 Research Center, Faculty of Medicine Ramathibodi Hospital, Mahidol University, Bangkok, Thailand; 12 Institute of Precision Medicine, National Sun Yat-Sen University, Kaohsiung, Taiwan; 13 Department of Pathology, School of Medicine, College of Medicine, Kaohsiung Medical University, Kaohsiung, Taiwan

**Keywords:** SLC14A1, arginine, urea, HK2, DEGS1, MTOR

## Abstract

Urothelial carcinoma (UC), including upper tract urothelial carcinoma (UTUC) and urinary bladder urothelial carcinoma (UBUC), is a common malignant disease in developed countries. Oncogenic metabolic lesions have been associated with UC development.

**Methods:** Using data mining, a series of studies were performed to study the involvement of SLC14A1 in UC specimens, animal models and UC-derived cell lines.

**Results:** In two cohorts of UTUC (*n* = 340) and UBUC (*n* = 295), the SLC14A1 protein level was an independent prognostic factor. Epigenetic silencing contributed to SLC14A1 downregulation in UCs. Total and membranous SLC14A1 played tumor suppressive roles through the inhibition of cell proliferation and metastasis in distinct UC-derived cells and animal models. Functional SLC14A1 prevented the accumulation of arginine and urea, enhanced mitochondrial fusion and aerobic respiration, inhibited glycolysis by altering the expression levels of several related proteins and sensitized arginine-deprivation treatment in *ASS1*-deficient UC-derived cells. In vitro and in vivo, SLC14A1 inhibited the mTOR signaling pathway and subsequently tumorigenesis, supported by reduced arginine concentrations in vitro. Nuclear SLC14A1 transrepressed *HK2* and *DEGS1* genes via recruitment of HDAC1 and/or SIN3A to maintain metabolic homeostasis and thereafter impeded tumorigenesis.

**Conclusion:** Clinical associations, animal models and in vitro indications provide solid evidence that the *SLC14A1* gene is a novel tumor suppressor in UCs. Total and membranous SLC14A1 prevents urea and arginine accumulation via the mTOR signaling pathway. Nuclear SLC14A1 recruits HDAC1 to transrepress oncometabolite genes.

## Introduction

Urothelial carcinoma (UC), also known as transitional cell carcinoma, is one of the most common malignancies worldwide 1. Among UCs, urinary bladder urothelial carcinoma (UBUC) accounts for the majority of UC (90-95%), while only ~5-10% of UC cases occur in the upper urinary tract (upper tract urothelial carcinoma, UTUC) 2. An unusually high prevalence of UTUC has been reported in certain areas including Taiwan 3. Environmental, infectious and genetic factors impact UC development 4-6. Genetic and clinical heterogeneity identified in UC patients additionally complicates the usage of standard therapies. To avoid side and adverse effects of chemotherapies, targeted therapies with drugs directed at specific molecular pathways have emerged as encouraging strategies to improve patient outcomes.

The human *SLC14A1* gene encoding the type B urea transporter protein was mapped to chromosome 18q12.3, adjacent to another urea transporter, *SLC14A2*, locus 7. This protein was found to be expressed in multiple cells and tissues including erythrocytes, kidneys and bladder. SLC14A1 facilitates passive urea transport, which is responsible for establishing an osmotic gradient in the inner medulla and preventing intracellular toxicity in extrarenal cells 8. In *SLC14A1* knockout mice, high urea concentrations caused DNA damage and apoptosis in bladder urothelium 9. Recently, a meta-analysis of genome-wide association studies of UBUCs revealed that *SLC14A1*-rs10775480, a variant at intron 6, is highly associated with susceptibility in bladder cancer 10, suggesting that *SLC14A1* may play a causal or regulatory role.

High *SLC14A1* mRNA was correlated with low-stage lung adenocarcinoma 11 as well as benign and androgen-divested prostate cancers 12. High-grade UBUCs displayed low *SLC14A1* mRNA and its corresponding protein levels 13. Accordingly, it is rational to hypothesize that SLC14A1 downregulation possibly leads to the accumulation of urea followed by the accumulation of cytotoxic agents in the urothelial cells, inducing critical damage. We therefore aimed to study the correlations between the SCL14A1 protein level and clinicopathological features, its potential regulatory elements, downstream targets and underlying molecular signaling pathway(s) by using two independent UTUC and UBUC cohorts, animal models and distinct UTUC- and UBUC-derived cell lines.

## Materials and methods

### Data mining, tumor materials, patients, QuantiGene assay and immunohistochemistry

Data mining is described in the Supplementary materials. For the QuantiGene branched DNA assay and immunohistochemistry, the Institutional Review Board of Chi Mei Medical Center approved the retrospective retrieval (IRB10302015) of 42 and another 340 primary UTUCs, as well as 36 and another 295 UBUCs with available tissue blocks from patients who underwent surgical treatment with curative intent between 1996 and 2004, while samples from those who underwent palliative resection were excluded (Supplementary materials). To determine the clinical relevance of the *SCL14A1* transcript level, 36 UBUCs and 42 UTUCs with normal, pTa-pT1 and pT2-pT4 specimens were evaluated. For immunohistochemistry, another 340 primary UTUCs and 295 UBUCs with available tissue blocks were used (Supplementary materials). One specific probe targeting the *SLC14A1* transcript was designed for QuantiGene™ Sample Processing Kit, formalin-fixed paraffin-embedded (FFPE) samples (QS0107, ThermoFisher, USA) and QuantiGene™ Plex Assay Kit (QP1013, ThermoFisher) based on the user guides (Supplementary materials). Immunohistochemical (IHC) staining was performed on representative tissue sections cut from FFPE tissues at 4-µm thickness as in our previous study 14 by probing specific anti-human antibodies (Supplementary materials).

### Cell culture and preparation of replication-incompetent viruses for stable overexpression and knockdown of specific genes in UC-derived cells

UBUC-derived cell lines including J82 (ATCC, USA), UMUC3 (ATCC) and BFTC905 (Food Industry Research and Development Institute, Taiwan) and one UTUC-derived cell line, RTCC1 (from Professor LC Chiang, Kaohsiung Medical University, Taiwan) 15, were used. Cell line authentication was performed by short tandem repeat DNA profiling (Mission Biotech, Taiwan). Culture conditions and preparation of replication-incompetent viruses are described in the Supplementary materials. Cells were transduced with viral particles carrying the target gene or small hairpin RNA (shRNA), and stable clones were selected with 2 μg/mL puromycin.

### Quantitative RT-PCR, immunoblot analysis, next-generation and bisulfite sequencing

Quantitative RT-PCR, immunoblot analysis and extraction of total cell lysate and membranous and nuclear proteins are described in the Supplementary materials. The total genomic DNA was extracted and used to construct the coding sequences library of the *SLC14A1* gene. Purified amplicons from the library were ligated to adapters with barcodes and loaded into a MiSeq System (Illumina, USA) for exon 3-11 sequencing. The bioinformatics analysis workflow was performed (Supplementary materials). One GC-rich region in the *SLC14A1* promoter was identified with MethPrimer software 16. Genomic DNA was extracted using the QIAamp DNA Mini Kit (Qiagen, Hilden, Germany) and subjected to bisulfite conversion with the EpiTect Fast DNA Bisulfite Kit (Qiagen) followed by a pyrosequencing assay (PyroMark Q24 system, Qiagen). Bisulfite sequencing for genomic DNA from UC tissues was performed by Mission Biotech (Supplementary materials).

### Quantitative chromatin immunoprecipitation

The quantitative chromatin immunoprecipitation assay was performed using the SimpleChIP® Enzymatic Chromatin IP Kit (#9003, Cell Signaling, USA). Cells were treated with paraformaldehyde to crosslink proteins and DNA, followed by glycine treatment to terminate the reaction. Chromatin was digested into 150- to 900-bp DNA/protein complexes by micrococcal nuclease. Sonication was further used to breakdown the nuclear membrane. Fragmented crosslinked chromatins were collected and incubated with the complex of protein G and the antibody against H3K27me3 (#ab6002, Abcam, UK), H3K9me2/3 (#5327, Cell Signaling) or HDAC1 (#34589, Cell Signaling). Purified ChIP DNA was subjected to quantitative PCR (Supplementary materials).

### Chemicals, site-directed mutagenesis, plasmids, in vitro and in vivo assays

All chemicals were purchased from Sigma‐Aldrich (USA) unless stated otherwise. The pLVX-puro-6HIS-*SLC14A1_*v1 plasmid served as the template to generate a double-mutant pLVX-puro-6HIS-*SLC14A1-C25S/C30S*_v1 using the QuikChange Lightning Site-Directed Mutagenesis Kit (Agilent, USA) (Supplementary). The *SLC14A1* gene was ligated to a DNA fragment corresponding to the SV40 nuclear localization signal (NLS: PKKKRKV) at the 3' end to generate the pLVX-pur-pHIS-SLC14A1-NLS_v1 plasmid. Cell viability, proliferation, migration, invasion and tube formation with HUVECs were performed according to the methods used in our previous study 17 and are shown in the Supplementary materials.

A plasmid containing a mitochondrial targeting sequence fused to a red fluorescence tag (pLV-MitoDsRed) was used to generate replication-incompetent lentivirus. Transduction of the lentiviral particles containing MitoDsRed showed the morphology and localization of each mitochondrion within a single cell, and 4',6-diamidino-2-phenylindole (DAPI) staining was used to identify nuclei. Mitochondrial fusion and fission were measured as in our previous study 18. For immunocytofluorescence, cells were fixed with paraformaldehyde and permeabilized with Triton X-100. After blocking with BSA, cells were incubated with anti-human SLC14A1 (ab23872, Abcam), plasma membrane markers and conjugated secondary antibodies (Supplementary). Cells were visualized with a confocal microscope for mitochondrial morphology and immunostaining in cellular compartments.

The cellular oxygen consumption rate (OCR) and extracellular acidification rate (ECAR) were measured using the Seahorse XFp Analyser (Agilent) according to the manufacturer's instructions (Supplementary). Glucose uptake (colorimetric, #ab136955, Abcam) was performed according to the manual. NOD/SCID mice (LASCO, Taiwan) were randomly grouped and subjected to subcutaneous and tail vein injections with UMUC3 cells (Supplementary materials). All animal treatments (#109041701) were performed according to the guidelines of Institution Animal Care and Use Committee, and all the protocols were approved by the Chi-Mei Medical Center, Tainan, Taiwan.

### Coimmunoprecipitation

Coimmunoprecipitation was performed using the Pierce™ Co-Immunoprecipitation Kit (ThermoFisher). Protein lysates were incubated with anti-human SLC14A1 antibody and subsequently, magnetic beads. The protein complexes were eluted from antibody/beads after washing and subjected to immunoblot analysis by probing anti-SIN3A (Proteintech, USA), -HDAC1 (Cell Signalling, USA), -ARID4B (Proteintech) or -SUDS3 (Novus Biologicals, USA) antibody.

### Luciferase reporter assay

pKM2L-phHKII (region #1, RDB05882, RIKEN, Renilla luciferase) and pGL4.54[luc2/TK] Vector (E5061, Promega, USA) were cotransfected into the *mock*- and *SLC14A1-NLS*-overexpressing J82 cells in a 96-well white plate by using PolyJet™ In Vitro DNA Transfection Reagent (Signagen®, USA). After transfection for 24 h, the medium was replaced and incubated for another 24 h. The luminescence intensities were measured using the Dual-Glo® Luciferase Reporter Assay System (Promega) (Supplementary).

### Statistics

Statistical analysis was performed using the SPSS software (version 14.0, IBM, USA). The protein expression levels of SLC14A1 and those involved in glycolysis and mTOR activation as determined by IHC were dichotomized into high (≥ median H-score) and low (< median H-score). The associations of these proteins were evaluated by Chi-square test. The correlations between the SLC14A1 protein level and specific clinicopathological parameters were evaluated using the Chi-square test. The disease-specific survival (DSS) and metastasis-free survival (MeFS) were estimated by the log-rank test and plotted using Kaplan-Meier curves. A multivariate Cox proportional hazards model was used to evaluate independent prognostic impacts for selected parameters. A two-tailed *t*-test was used to examine significant differences in the relative mRNA levels, different phases of the cell cycle, percentages of cell viability, ability of cell migration, invasion, HUVEC tube formation and mouse xenografts (tumor volume). The comparison of *SLC14A1* transcription levels between different groups were determined using the Mann-Whitney U test.

## Results

### SLC14A1 downregulation confers a poor prognosis in UC patients

**Figure 1:**
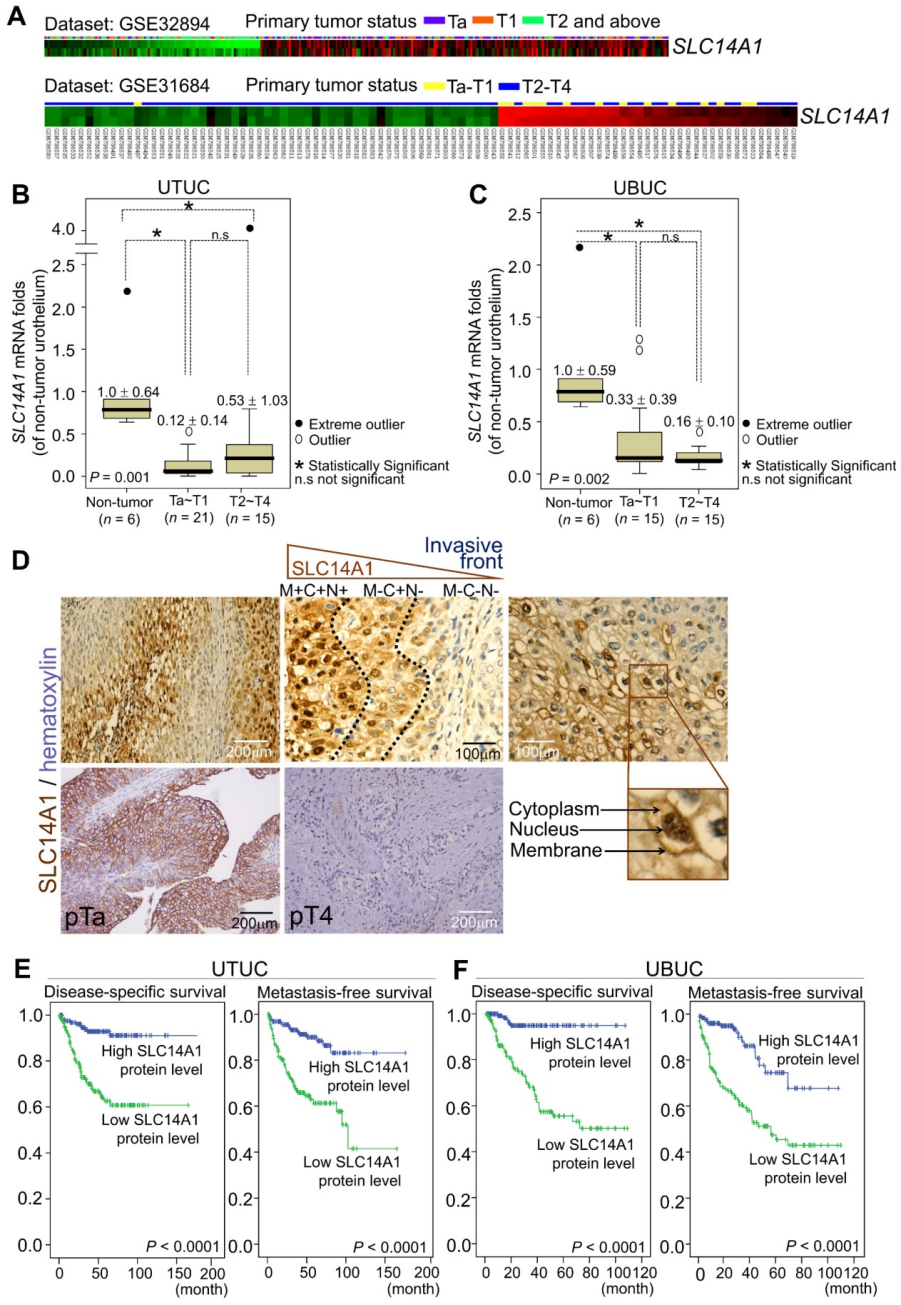
** Downregulation of the *SLC14A1* mRNA levels are correlated with poor clinical outcomes, and low SLC14A1 protein levels confer worse survival in UTUC and UBUC patients. (A)** Repeated analysis of the transcriptomes of UBUCs from the GEO database (GSE32894 and GSE31684) demonstrated that the *SLC14A1* transcript is downregulated during UBUC progression in two independent datasets. Heat maps from both datasets are shown, and red and green lines represent upregulated and downregulated *SLC14A1* mRNA levels, respectively, in different specimens. The primary tumor status is shown above each tissue specimen. **(B, C)** In two subsets of UTUC and UBUC patients, the QuantiGene branched DNA assay identified that the *SLC14A1* mRNA level (the means ± SD) was decreased during progression. A distinction between outliers that are more than 1.5 box lengths from one hinge of the box (using a circle) and outliers that are more than 3 box lengths from a hinge (using an asterisk) is present. **(D)** Immunohistochemistry showed high SLC14A1 protein levels in the membrane, cytoplasm and nucleus in low-stage and low-grade urothelial carcinomas but low expression in high-stage and invading UCs. In the process of tumor cell invasion, the SLC14A1 protein level initially declined at the membranous (from M+ to M-) and nuclear (from N+ to N-) compartments and subsequently disappeared from the cytoplasm (from C+ to C-). **(E, F)** Kaplan-Meier plot estimating that a low SLC14A1 protein level confers poor prognoses in terms of disease-specific and metastatic-free survival in UTUC (*n* = 340) and UBUC (*n* = 295) patients. Statistical significance: *P < 0.05; n.s: not significant.

In two Gene Expression Omnibus (GEO) datasets, GSE32894 and GSE31684, downregulation of the *SLC14A1* transcripts was found to be significantly correlated with a high pT status in UBUC patients (Figure 1A, Supplementary Table S1, Table S2). In the UTUC (*n* = 42) and UBUC (*n* = 36) subsets, *SLC14A1* mRNA levels were downregulated in tumor specimens compared to their normal counterparts (Figure 1B, 1C). High SLC14A1 protein levels were correlated with early primary tumor status (*P* < 0.001), negative nodal metastasis (*P* < 0.001), low histological grade (*P* < 0.001), negative vascular invasion (*P* < 0.001), negative perineural invasion (*P* < 0.05) and low mitotic rate (*P* < 0.01) in two large cohorts of UTUC (*n* = 340) and UBUC (*n* = 295) patients (Supplementary Table S3). Interestingly, SLC14A1 protein is expressed in the cytoplasm, nucleus and cell membrane. In the front end, during the invasion process, SLC14A1 protein levels gradually decreased and eventually vanished in the cytosol (Figure 1D). Moreover, univariate and multivariate analyses indicated that, in addition to several important clinicopathological parameters, high SLC14A1 protein levels predict high disease-specific, metastasis-free survivals (*P* < 0.0001; Figure 1E, 1F) and serve as an independent prognostic marker in UTUC (Table 1) and UBUC patients (Table 2). Thus, SLC14A1 plays a clinical role as a tumor suppressor in UCs.

### Epigenetic silencing contributes to SLC14A1 downregulation in UCs

**Figure 2:**
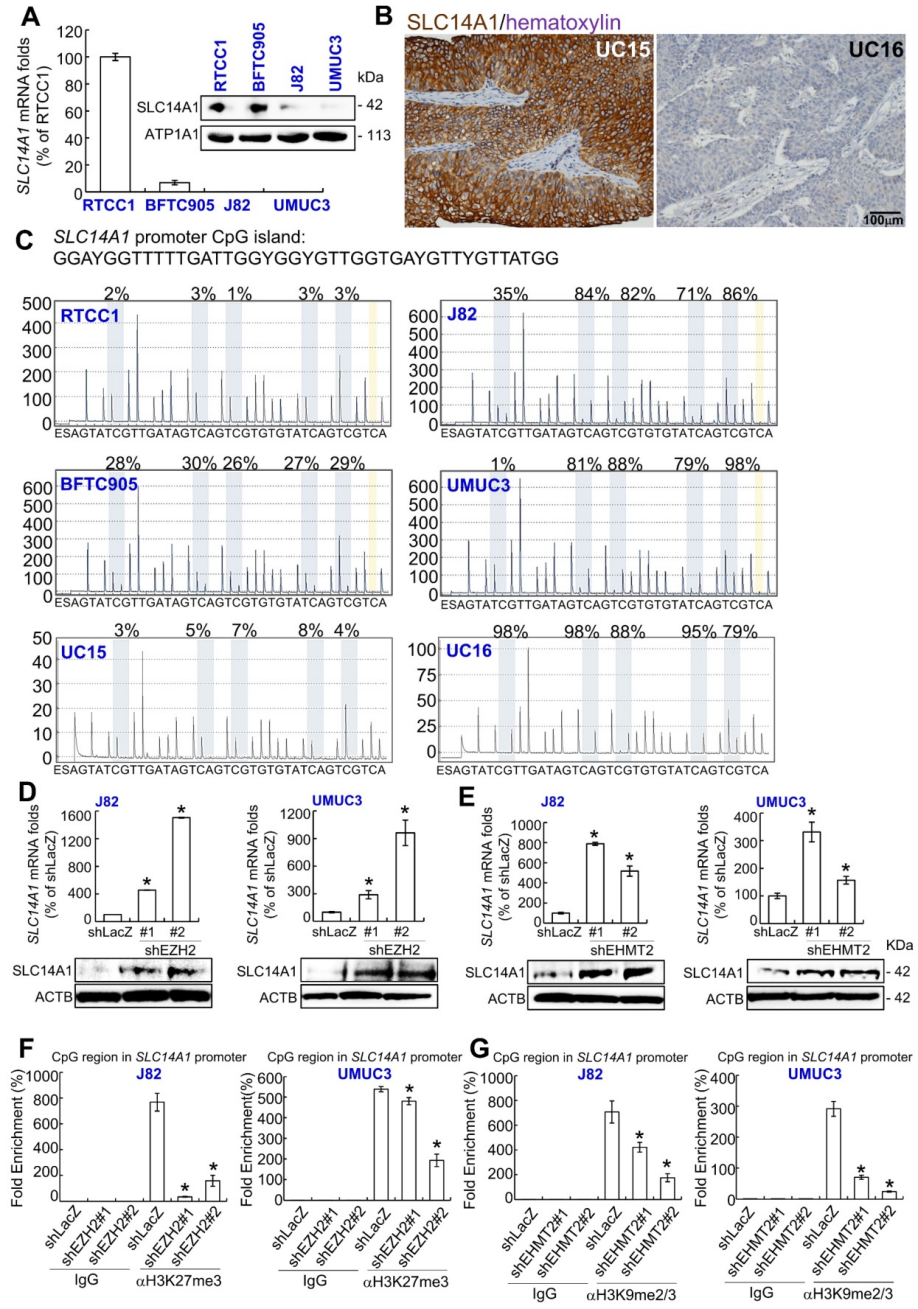
** Impact of epigenetic modifications on *SLC14A1* mRNA and its corresponding protein levels in distinct UC-derived cells and tumor specimens. (A)** Quantitative RT-PCR and immunoblot analysis indicated that *SLC14A1* mRNA and its corresponding protein are highly expressed in RTCC1 cells, while they are barely detected in J82 and UMUC3 cells. **(B)** Immunohistochemistry showed that SLC14A1 protein is highly expressed in UC15 cells (a low-grade Ta specimen), whereas it is lowly expressed in UC16 cells (a high-grade T2 specimen). **(C)** A quantitative DNA methylation analysis identified low methylation burdens in the CpG island (a large number of CpG dinucleotide repeats that are located within and close to sites of approximately 40% of mammalian gene promoters) of the *SLC14A1* promoter region in RTCC1 and BFTC905 cells and UC15 specimens with high *SLC14A1* levels. However, hypermethylation was found in J82 and UMUC3 cells and UC16 tumors with low *SLC14A1* levels. **(D-E)** Stable knockdown of the *EZH2* or *EHMT2* gene with 2 distinct shRNA clones notably upregulated *SLC4A1* mRNA and the corresponding protein levels in J82 and UMUC3 cells. **(F-G)** A quantitative ChIP assay performed by probing anti-H3K27me3 (αH3K27me3) or -H3K9me2/3 (αH3K9me2/3) antibody and quantitative PCR demonstrated that knockdown of the *EZH2* or *EHMT2* gene suppressed histone methylation status at the CpG island of the *SLC14A1* promoter region compared to that of the control (shLacZ) in J82 and UMUC3 cells. All experiments were performed in triplicate, and the results are expressed as the mean ± SD. For immunoblot analysis and immunohistochemistry, representative images are shown. Actin, beta (ACTB) and IgG served as a loading and negative control, respectively, for the immunoblot and quantitative ChIP assay, respectively. Statistical significance: *P < 0.05.

As shown in Figure 2A, *SLC14A1* mRNA and its corresponding protein levels were highly expressed in RTCC1 compared to other UC-derived cell lines. SLC14A1 protein was highly expressed in low-grade Ta specimens compared to high-grade ones (Figure 2B). However, no variant/mutation was found in the coding DNA sequence of the *SLC14A1* gene in the following UC-derived cell lines: RTCC1, BFTC905, J82, UMUC3 (Supplementary Table S4) and 67 UC specimens with various SLC14A1 protein levels (Supplementary Table S5), implying that other regulatory machinery may impact *SLC14A1* mRNA and its corresponding protein levels in UC patients.

One CpG island between exons 2 and 3 in the *SLC14A1* gene was identified (Supplementary Figure S1A). Hypermethylation from positions 2 to 5 of the *SLC14A1* promoter was found in J82 and UMUC3 cells (Supplementary Table S4). In 67 clinical specimens, hypermethylation at position 1 or 5 of the *SLC14A1* promoter was negatively correlated with SLC14A1 protein levels (*P* < 0.05, Supplementary Table S5 and data not shown). Treatment with 5-aza-2′-deoxycytidine (5-Aza) in J82 and UMUC3 cells upregulated *SLC14A1* mRNA and its corresponding protein levels (Supplementary Figure S1B). Low methylation burdens were found in RTCC1 and BFTC905 cells and specimens with high SLC141 protein levels, while high methylation rates in J82, UMUC3 cells and specimens with low SLC14A1 protein levels were detected in the CpG region of the *SLC14A1* gene (Figure 2C, Supplementary Table S4, S5). These observations indicate that *SLC14A1* promoter hypermethylation downregulates its mRNA and protein levels in UC-derived cells and UC tissues. Our findings were also supported by cases of invasive bladder cancer deposited in The Cancer Genome Atlas (TCGA) database (http://www.cbioportal.org/), indicating that *SLC14A1* mRNA levels and methylation status are negatively correlated (*n* = 408, *P* ~0, Supplementary Figure S2).

Reappraisal on the public genome-wide database showed negative correlations between *SLC14A1* and *EZH2* mRNA levels in a fetal lung-derived TIG-3 cell line (Supplementary Figure S3). Thus, J82 and UMUC3 cells were treated with an EZH2 (an H3K27 methyltransferase) inhibitor, DZNep; an EHMT2 (an H3K9 methyltransferase) inhibitor, UNC0638; and a histone lysine methylation inhibitor (α-ketoglutarate/AKG).

Treatment with any of the above chemicals upregulated *SLC14A1* mRNA and the corresponding protein levels (Supplementary Figure S4A-S4C). Stable knockdown of the *EZH2* and *EHMT2* genes downregulated *EZH2* and *EHMT2* mRNA and the corresponding protein levels, respectively (Supplementary Figure S4D, S4E), while upregulated *SLC14A1* mRNA and corresponding protein levels were found (shLacZ) in J82 and UMUC3 cells compared to the control (Figure 2D, 2E). Treatment with AKG decreased H3K27me3 and H3K9me2/3 histone methylation levels in the CpG region of the *SLC14A* promoter compared to the control (H_2_O) (Supplementary Figure S4F, S4G). Stable knockdown of the *EZH2* and *EHMT2* genes further suppressed histone methylation states in the *SLC14A1* promoter CpG region in J82 and UMUC3 cells, respectively (Figure 2F, 2G). All of these findings suggested that SLC14A1 downregulation in UCs is attributed to epigenetic silencing.

### Total and membranous SLC14A1 play tumor suppressive roles in vitro and in vivo

**Figure 3:**
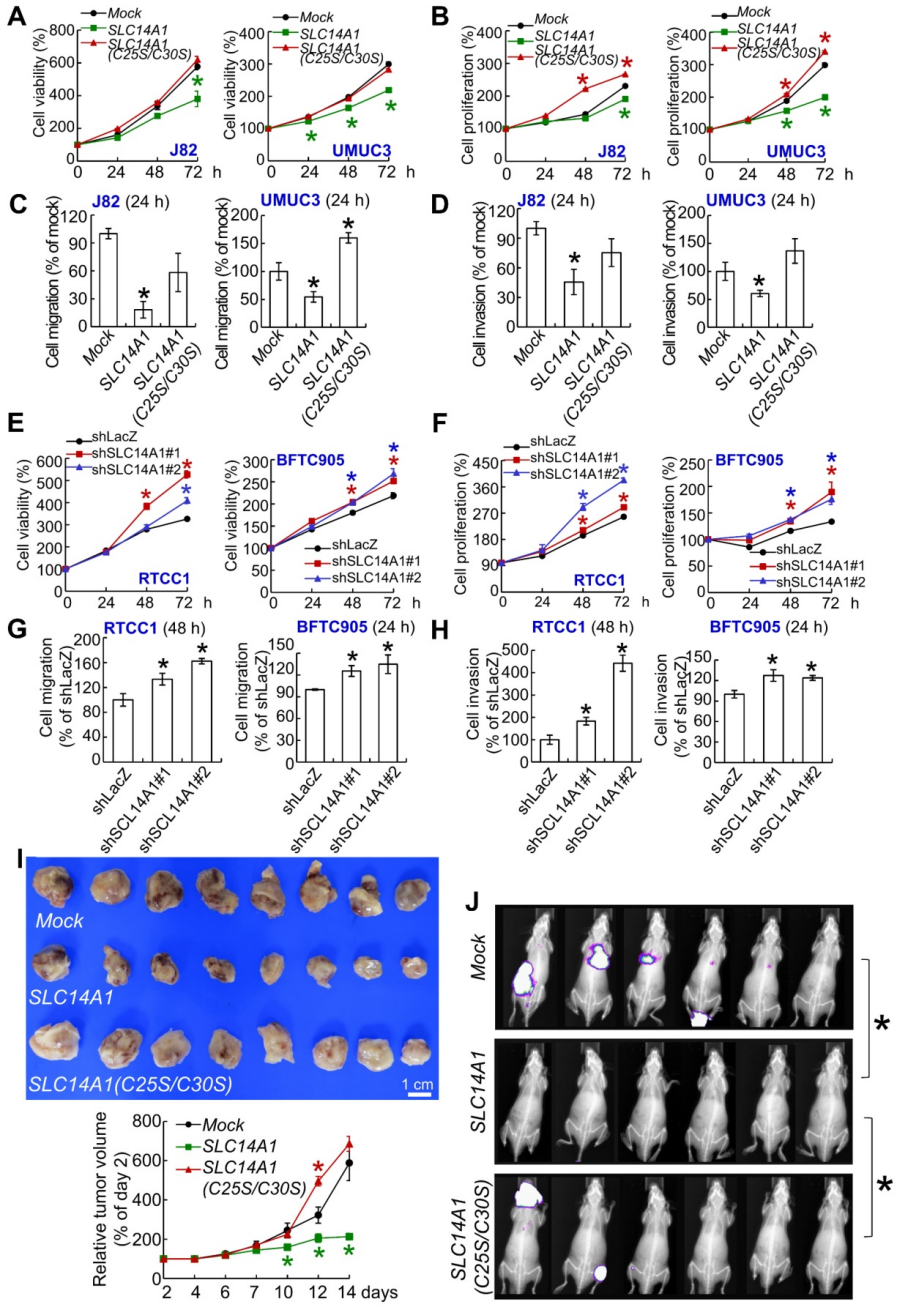
***SLC14A1* plays a tumor suppressive role in vitro and in vivo.** XTT, cell proliferation and Boyden chamber assays were performed to examine cell viability, proliferation, migration and invasion in vitro. **(A-D)** Compared to the control (*mock*), overexpression of the *SLC14A1* gene decreased while overexpression of the double mutation *SLC14A1(C25S/C30S)* gene (defects in membrane trafficking) restored cell viability **(A)**, cell proliferation **(B)**, cell migration **(C)** and cell invasion **(D)** at each indicated time point in J82 and UMUC3 cells. On the other hand, knockdown of the *SLC14A1* gene with 2 distinct shRNA clones in RTCC1 and BFTC905 cells increased cell viability, proliferation, migration and invasion at each indicated time point compared to the shLacZ control **(E-H)**. **(I)** In a mouse xenograft model, subcutaneous injection of *SLC14A1*-carrying UMUC3 cells (*n* = 8) reduced tumor growth, while *SLC14A(C25S/C30S)*-carrying UMUC3 cells (*n* = 8) enhanced tumor growth compared to the *mock* (*n* = 8) in NOD-SCID mice. **(J)** Tail vein injection of UMUC3 cells without manipulation (*mock*), *SLC14A1*- or *SLC14A(C25S/C30S)*-carrying UMUC3 cells in NOD/SCID mice (*n* = 6 for each group) were performed. Bioluminescence images captured by an in vivo imaging system (IVIS) showed that the tumor sizes were reduced in *SLC14A1*-overexpressing mice compared to the *mock* group (day 14), while lung metastases were identified in both the *SLC14A1(C25SC30S)*-overexpressing and *mock* groups at day 34 after injection.

A double mutation at cysteine 25 and 30 to serine (C25S/C30S) in the *SLC14A1* gene was created (Supplementary Figure S5A) to abolish its urea transport function following the deficiency in membranous SLC14A1 trafficking 19. Overexpression of *SLC14A1* and *SLC14A1(C25S/C30S)* genes was verified by mRNA upregulation (Supplementary Figure S5B). Overexpression of the *SLC14A1(C25S/C30S)* gene did not upregulate membranous (ATP1A1: membrane marker) or nuclear SLC14A1 protein (H2B: nuclear marker) level compared to those in *SLC14A1-*overexpressing J82 and UMUC3 cells (Supplementary Figure S5C, S5D). Overexpression of the *SLC14A1(C25S/C30S)* gene was not able to induce G_1_ cell cycle arrest, reduce HUVEC tube formation (Supplementary Figure S5E-S5G), suppress cell viability (Figure 3A), or decrease cell proliferation (Figure 3B) from 24 to 72 h after seeding, migration (Figure 3C), or invasion (Figure 3D), while it upregulated TYMS and DHFR protein levels in J82 and UMUC3 cells (Supplementary Figure S5H). On the other hand, knockdown of the *SLC14A1* gene in RTCC1 and BFTC905 cells downregulated *SLC14A1* mRNA (*P* < 0.05) and the corresponding protein levels, while it promoted cell cycle progression to S and G_2_ phases (*P* < 0.05; Supplementary Figure S6A, S6B), enhanced HUVEC tube formation (*P* < 0.05) (Supplementary Figure S6C), increased cell viability (*P* < 0.05), increased cell proliferation (*P* < 0.05) from 24 to 72 h after seeding (Figure 3E, 3F), promoted cell migration (*P* < 0.05) and cell invasion (*P* < 0.05) (Figure 3G, 3H) and upregulated TYMS and DHFR protein levels (Supplementary Figure S6D) compared to the control (shLacZ).

In mouse xenograft experiments, the average tumor size at day 14 after transplantation was smaller in *SLC14A1*-overexpressing UMUC3 cells than in the *mock* (*P* < 0.05), while it was restored to the similar size as the *mock* in xenografts from *SLC14A1(C25S/C30S)*-overexpressing cells (Figure 3I). TYMS and DHFR protein levels were downregulated in xenografts from *SLC14A1*-overexpressing UMUC3 cells, while they were restored in xenografts from *SLC14A1(C25S/C30S)*-overexpressing UMUC3 cells compared to the *mock* (Supplementary Figure S7A). Tail vein injection showed that *SLC14A1*-overexpressing UMUC3 cells suppressed metastasis development, whereas *mock* and *SLC14A1(C25S/C30S)*-overexpressing UMUC3 cells metastasized (Figure 3J) in the mouse model. High rates of lung metastasis were observed in *mock* (UMUC3 cells) and *SLC14A1(C25S/C30S)* compared to *SLC14A1*-overexpressing UMUC3 cells (Supplementary Figure S7B). Hence, a series of in vitro and animal model experiments suggested that SLC14A1 protein, particularly the membranous form, plays a tumor suppressive role in UC. Along with the observations from Figure 3A, 3B and Figure 3E, 3F, SLC14A1 may inhibit cell proliferation in vitro and in vivo accompanied by negative regulation of TYMS and DHFR protein levels.

### SLC14A1 prevents the accumulation of urea and arginine in vitro

As shown in Supplementary Figure S8A-S8F, overexpression of the *SLC14A1* gene downregulated arginine, urea, putrescine, spermidine and spermine, while it upregulated L-ornithine in J82 and UMUC3 cells. However, overexpression of the nonmembranous *SLC14A1(C25S/C30S)* gene in J82 and UMUC3 cells showed the opposite pattern. Knockdown of the *SLC14A1* gene in RTCC1 and BFTC905 cells displayed similar results to those from overexpression of the nonmembranous *SLC14A1(C25S/C30S)* gene, suggesting that SLC14A1 regularizes the urea cycle by continually releasing urea and subsequently maintains the catabolism of arginine and prevents excessive biogenesis of polyamines. Particularly, arginine was upregulated in *SLC14A1*-knockdown and* ASS1*-deficient RTCC1 cells (*P* < 0.05), indicating that SLC14A1-suppressed arginine synthesis is independent of *ASS1*. Manipulation of the SLC14A1 level was not able to consistently alter the mRNA abundance of several metabolic enzymes including *OTC*, *ASS1*, *ASL* and *ARG* in 4 UC-derived cell lines (Supplementary Figure S8B). Furthermore, the *SLC14A1* mRNA level was not correlated to *OCT*, *ASS1*, *ASL* or *ARG* mRNA levels in vivo (Supplementary Figure S8C). All of the above suggest that SLC14A1-prevented accumulation of arginine was due to regulation of the expression levels of these metabolic enzymes. On the other hand, in the process of polyamine biosynthesis, both ornithine decarboxylase (ODC) and arginine decarboxylase (ADC) are key enzymes for putrescine production 20. Since dysfunctional SLC14A1 downregulated L-ornithine (a precursor of canonical polyamine biosynthesis) but upregulated arginine and downstream polyamines (Supplementary Figure S8A-S8F), an alternative pathway via agmatinase to convert arginine to agmatine and then putrescine, which may be driven by dysfunctional SLC14A1, was considered. AZIN2 (an ADC) is a key enzyme to synthesize agmatine from arginine.

Thus, the expression levels of *SLC14A1* and *AZIN2* genes were adjusted to clarify their relationships. Stable knockdown of the *AZIN2* gene in RTCC1 and BFTC905 cells and in *SLC14A1*-knockdown RTCC1 and BFTC905 cells downregulated *AZIN2* mRNA and the corresponding protein levels compared to their respective controls, shLacZ, shSLC14A1#1 and shSLC14A1#2 (Supplementary Figure S8I, S8J). Double knockdown of *SLC14A1* and *AZIN2* genes further downregulated putrescine concentrations compared to knockdown the *SLC14A1* gene alone in RTCC1 and BFTC905 cells (Supplementary Figure S8I), suggesting that dysfunctional SLC14A1 upregulated arginine and downstream putrescine concentrations may be mediated through the AZIN2/agmatine axis.

### SLC14A1 enhances mitochondrial fusion and aerobic respiration yet inhibits glycolysis in UCs and sensitizes arginine-deprivation treatment in *ASS1*-deficient UC-derived cells

**Figure 4:**
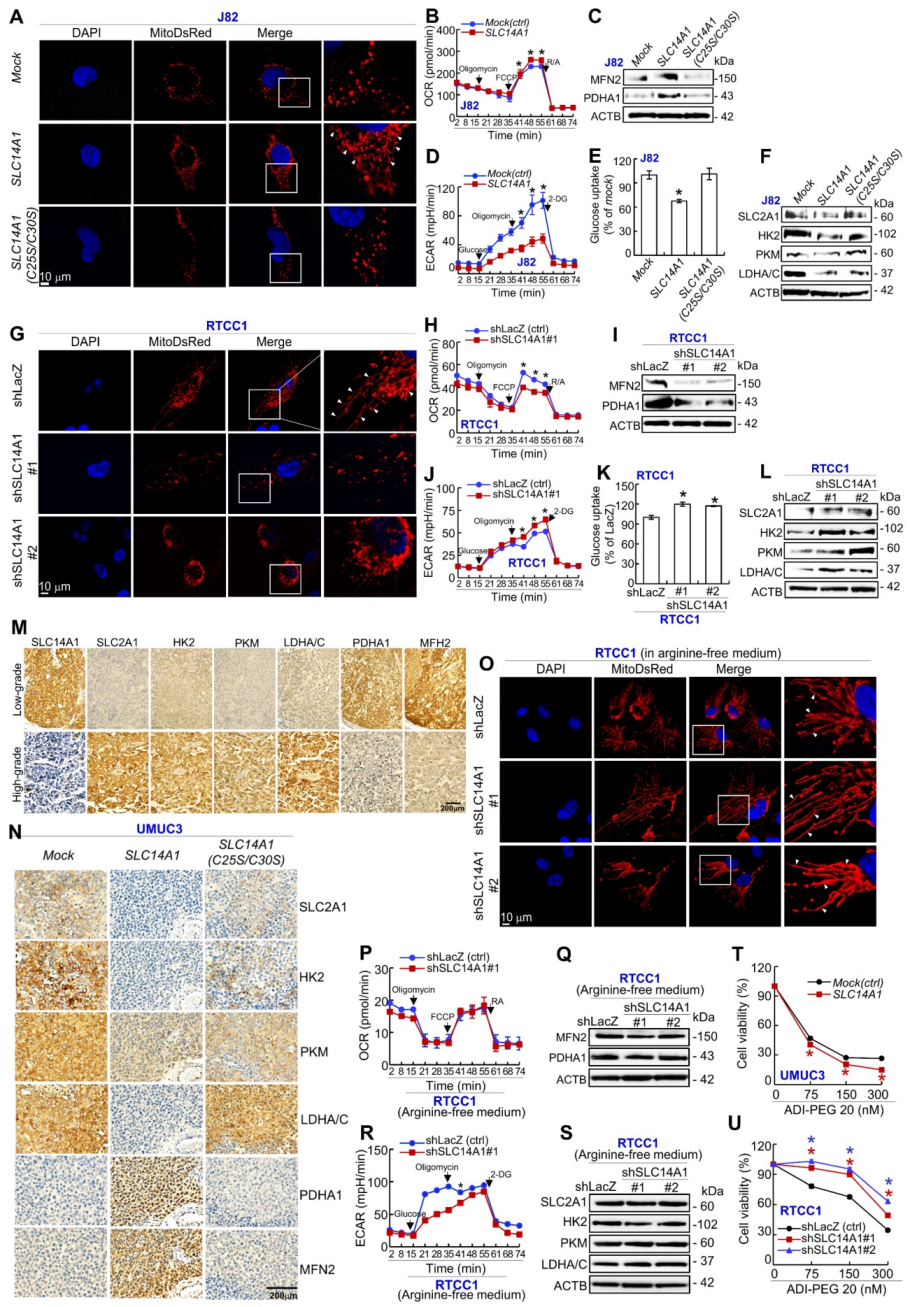
** Dysfunctional SLC14A1 protein induces metabolic reprogramming.** Cells were transduced with lentiviruses carrying a MitoDsRed tag to label each mitochondrion. Immunocytofluorescence and confocal microscopy, Seahorse XFp Analyser, immunoblot, glucose uptake, immunohistochemistry and XTT assays were performed to examine mitochondrial status, the oxygen consumption rate (OCR) and extracellular acidification rate (ECAR), protein levels, glucose uptake by cells and protein levels in cells and tissue specimens, respectively. **(A-F)** In J82 cells, stable overexpression of the *SLC14A1* gene suppressed mitochondrial fragmentation/fission **(A)**, increased the OCR **(B)**, upregulated MFN2 and PDHA1 protein levels **(C)**, decreased the ECAR** (D)** and glucose uptake **(E)** and downregulated SLC2A1, HK2, PKM and LDHA/C protein levels **(F)** compared to the *mock*. Similar to *SLC14A1(C25S/C30S)*-overexpressing J82 cells** (A-F)**, stable knockdown of the *SLC14A1* gene with 2 distinct shRNA clones in RTCC1 cells showed opposite phenotypes to those of *SLC14A1*-overexpressing J82 cells **(G-L)**. Immunohistochemistry showed that SLC14A1, PDHA1 and MFH2 were highly expressed, while SLC2A1, HK2, PKM, LDHA/C were barely expressed in low-grade, compared to high-grade UCs** (M),** and the protein levels of SLC2A1, HK2, PKM, and LDHA/C were downregulated, while PDHA1 and MFN2 were upregulated in xenografts from *SLC14A1*-overexpressing UMUC3 cells compared to the *mock*. Mutated *SLC14A1(C25S/C30S)* abolished these effects **(N)**. **(O-S)** Knockdown of the *SLC14A1* gene in *ASS1*-deficient RTCC1 cells in arginine-free medium showed mitochondrial fusion and a decreased ECAR, while other phenotypes remained unchanged. **(T, W)** Treatment with an arginine deiminase, ADI-PEG20, in *SLC14A1*-overexpressing UMUC3 cells decreased, while the same treatment in *SLC14A1*-knockdown RTCC1 cells increased cell viability at each indicated time point compared to the control (*mock* or shLacZ). For the OCR and ECAR measurements, the arrows indicate oligomycin, carbonyl cyanide-4-(trifluoromethoxy)phenylhydrazone (FCCP), rotenone (RA), glucose and 2-deoxy-glucose (2-DG) that were loaded at each indicated time point. All experiments were performed in triplicate, and the results are expressed as the mean ± SD. For immunocytofluorescence, immunoblot and immunohistochemistry, representative images are shown. Actin-beta (ACTB) served as a loading control for immunoblot analysis. Statistical significance: *P < 0.05.

Exogenous expression of the *SLC14A1* gene in J82 (Figure 4A-4F) and UMUC3 (Supplementary Figure S9A-S9F) cells enhanced mitochondrial fusion (Figure 4A, Supplementary Figure S9A), increased the OCR at the indicated time points (*P* < 0.05; Figure 4B, Supplementary Figure S9B), upregulated the levels of a mitochondrial membrane protein, MFN2 (one GTPase), which participates in mitochondrial fusion and as a key enzyme and PDHA1, which catalyzes pyruvate to acetyl coenzyme A (acetyl-CoA) (Figure 4C, Supplementary Figure S9C), whereas it decreased ECAR (Figure 4D, Supplementary Figure S9D) and glucose uptake (Figure 4E, Supplementary Figure S9E) and downregulated SLC2A1 (GLUT-1), HK2, PKM and LDHA/C protein levels (Figure 4F, Supplementary Figure S9F). However, overexpression of the mutated *SLC14A1(C25S/C30S)* gene in J82 and UMUC3 cells promoted mitochondrial fission (Figure 4A, Supplementary Figure S9A), downregulated MFN2 and PDHA1 protein levels (Figure 4C, Supplementary Figure 9C), increased glucose uptake (Figure 4E, Supplementary Figure S9E) and increased SLC2A1, HK2, PKM and LDHA/C abundances (Figure 4F, Supplementary Figure S9F). Knockdown of the *SLC14A1* gene with 2 distinct shRNA clones in RTCC1 (Figure 4G-4L) and BFTC905 (Supplementary Figure S9G-S9L) cells showed thoroughly opposite phenotypes compared to overexpression of the wild-type *SLC14A1* gene in J82 and UMUC3 cells. Quantification of the mitochondrial morphological changes are shown in Supplementary Figure 9M based on our previous study 18 (see also the Supplementary material).

Compared to high-grade UCs, SLC14A1, PDHA1 and MFH2 proteins were highly expressed, while SLC2A1, HK2, PKM and LDHA/C proteins were barely expressed in low-grade UCs (Figure 4M). Accordingly, the SLC14A1 protein level was negatively correlated to those of SLC2A1, HK2, PKM and LDHA/C, while it was positively correlated to those of PDHA1 and MFN2 in UTUCs (*n* = 170) and UBUCs (*n* = 148) (Supplementary Table S6, S7). In murine xenografts from *SLC14A1*-overexpressing UMUC3 cells, the protein levels of SLC2A1, HK2, PKM, and LDHA/C were notably downregulated, while MFN2 and PDHA1 were upregulated, and overexpression of *SLC14A1(C25S/C30S)* counteracted these effects in UMUC3 cells (Figure 4N). All of these findings suggested that SLC14A1 enhances mitochondrial fusion and aerobic respiration, while it inhibits mitochondrial fission and aerobic glycolysis in vitro, in UC patients and in vivo.

Interestingly, under arginine-deprivation, knockdown of the *SLC14A1* gene in *ASS1*-deficient RTCC1 cells showed mitochondrial fusion instead of fission (Figure 4O), while the OCR (Figure 4P), MFN2 and PDHA1 protein levels remained constant (Figure 4Q), ECAR was decreased (Figure 4R), and the expression levels of SLC2A1, HK2, PKM and LDHA/C were not altered (Figure 4S), suggesting that arginine-deprivation neutralized the effects of dysfunctional *SLC14A1*. Treatment with an arginine deiminase, ADI-PEG 20, in UMUC3 (*mock*) and *ASS1*-deficient RTCC1 (shLacZ) cells decreased cell viability (Figure 4T, 4W). ADI-PEG 20 treatment further reduced and increased cell viability in *SLC14A1*-overexpressing *ASS1*-positive UMUC3 cells (*P* < 0.05, Figure 4S) and *SLC14A1-*knockdown *ASS1*-negative RTCC1 cells (*P* < 0.05, Figure 4W), respectively, at each indicated time point compared to the corresponding control (*mock* and shLacZ). These observations indicated that regarding cell viability, arginine upregulation due to *SLC14A1-* knockdown in *ASS1*-deficient cells enhances the resistance to arginine removal by ADI-PEG 20. Accordingly, functional *SLC14A1* promotes mitochondrial fusion/respiration; nevertheless, it inhibits aerobic glycolysis along with altering the expression levels of several related proteins in UC specimens and UC-derived cells. Furthermore, upregulation of SLC14A1 sensitizes arginine-deprivation treatment in *ASS1*-deficiency cells.

### SLC14A1 suppresses the mammalian target of rapamycin signaling pathway in vitro and in vivo

**Figure 5:**
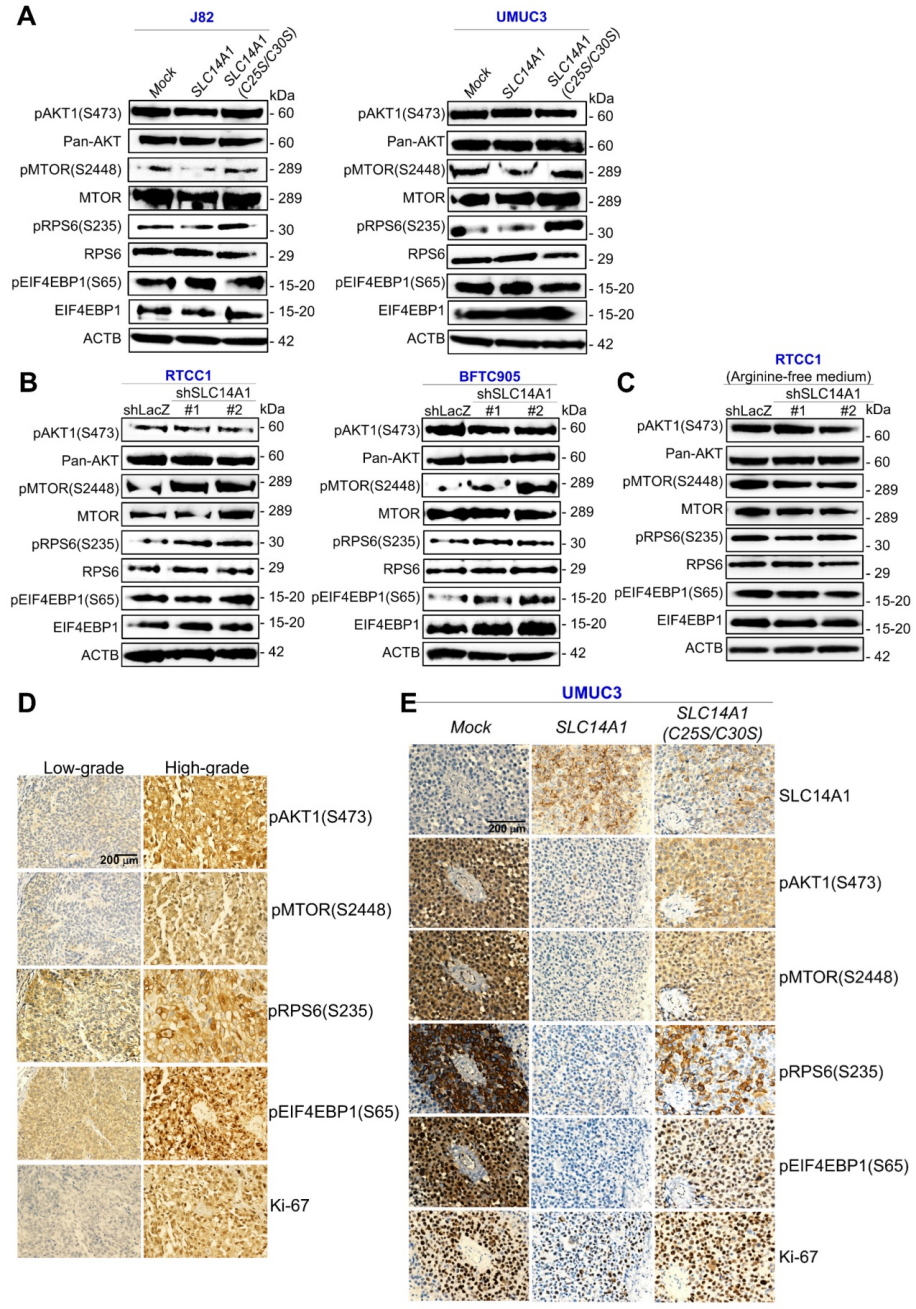
** Total and membranous SLC14A1 proteins suppress the mammalian target of rapamycin (mTOR) signaling pathway in vitro and in vivo.** Immunoblot analysis and immunohistochemistry were conducted. **(A)** pMTOR(S2448) and pRPS6(S235) (active, phosphorylated forms) were downregulated in stable *SLC14A1*- J82 and UMUC3 cells, while they were upregulated in stable *SLC14A1(C25S/C30S)*-overexpressing J82 and UMUC3 cells. All other examined proteins remained unchanged. **(B)** Instead, stable knockdown of the *SLC14A1* gene with 2 distinct shRNA clones in RTCC1 (*ASS1*-deficient) and BFTC905 cells notably upregulated pMTOR(S2448) and pRPS6(S235) protein levels. **(C)** Knockdown of the *SLC14A1* gene with 2 distinct shRNA clones in *ASS1*-deficient RTCC1 cells in arginine-free medium was not able to upregulate the pMTOR(S2448) or pRPS6(S235) protein level. Immunohistochemistry showed that pAKT1(S473), pMTOR(S2448), pRPS6(S235), pEIF2EP1(S65) and Ki-67 were highly expressed in high-grade compared to low-grade UCs **(D)**, and SLC14A1 was upregulated, while pAKT1(S473), pMTOR(S2448), pRPS6(S235), pEIF4EBP1(S65) and Ki-67 were downregulated in xenografts from *SLC14A1*-overexpressing UMUC3 cells compared to the *mock*. Except for the SLC14A1 protein, the immunostaining pattern in xenografts from *SLC14A1(C25S/C30S)*-overexpressing UMUC3 cells was similar to those of the *mock*. For immunoblot analysis and immunohistochemistry, representative images are shown and actin, beta (ACTB) served as a loading control for immunoblot analysis.

Reappraisal on a public genome-wide database showed that among a series of cell lines, high mRNA levels of *SLC14A1* (*n* = 19) were sensitive, while low *SLC14A1* (*n* = 7) cell lines were resistant to a PI3K/mTOR dual inhibitor, BEZ235, treatment (Supplementary Figure S10), suggesting that functional *SLC14A1* may intervene in the PI3K/mTOR axis. Since SLC14A1 suppressed cell proliferation and downregulated arginine in this study and arginine is one key activator of the mammalian target of rapamycin complex 1 (mTORC1) 21, we next examined how SLC14A1 regulated the mTOR pathway. Of several molecules involving in mTOR signaling, active/phospho-MTOR (S2448) [pMTOR(S2448)] and pRPS6(S235) were notably downregulated in *SLC14A1*-overexpressing cells but restored in *SLC14A1(C25S/C30S)*-overexpressing J82 and UMUC3 cells as well as in *SLC14A1*-knockdown RTCC1 and BFTC905 cells. However, the level of pAKT1(S473) or pEIF4EBP1(S65) protein in the abovementioned cells was not consistently correlated to the *SLC14A1* status (Figure 5A, 5B), signifying that SLC14A1-inhibited mTOR pathways may be irrelevant to Akt signaling. Intriguingly, under arginine deprivation, pMTOR (S2248) and pRPS6(S235) were not upregulated in *SLC14A1*-knockdown *ASS1*-deficienct RTCC1 cells (Figure 5C), indicating that arginine is essential for MTOR activation upon *SLC14A1-*knockdown in vitro.

Compared to low-grade UCs, pAKT1(S473), pMTOR(S2448), pRPS6(S235), pEIF2EP1(S65) and Ki-67 proteins were highly expressed in high-grade UCs (Figure 5D). Consequently, the SLC14A1 protein level was negatively correlated to those of pAKT1(S473), pMTOR(S2448), pRPS6(S235), pEIF4EP1(S65) and Ki-67 in UTUCs (*n* = 170) and UBUCs (*n* = 148) (Supplementary Table S6, S7). In murine xenografts from *SLC14A1*-overexpressing UMUC3 cells, SLC14A1 was upregulated, while pMTOR(S2448), pRPS6(S235) and Ki-67 were notably downregulated, and these downregulated proteins were restored in xenografts from *SCL14A1(C25S/C30S)*-overexpressing UMUC3 cells compared to the *mock* (Figure 5E), supporting in vitro and immunohistochemistry observations.

### Nuclear SLC14A1 plays a tumor suppressive role through recruitment of HDAC1 to transrepress *HK2* and *DEGS1* genes

**Figure 6:**
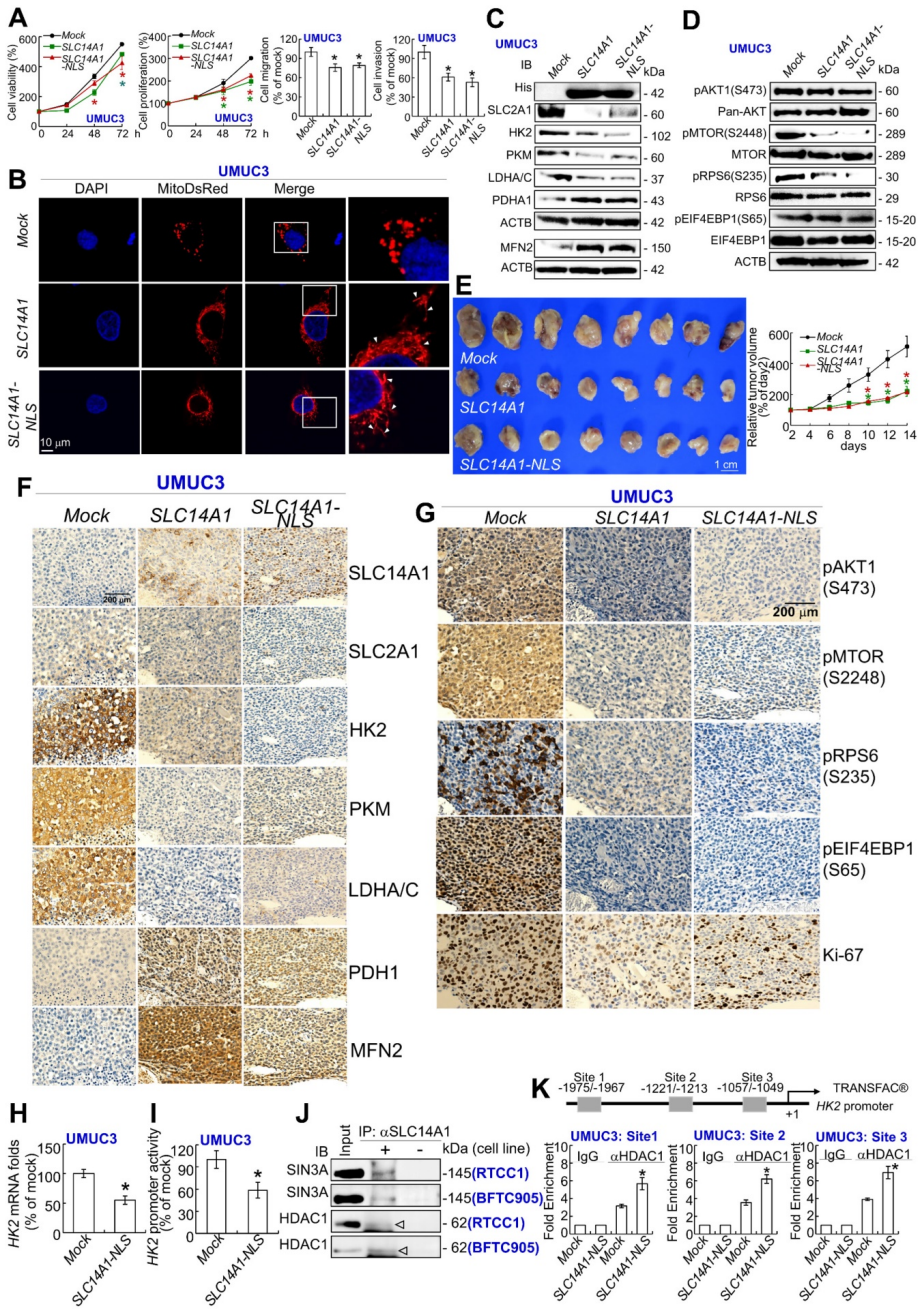
** Nuclear SLC14A1 plays a tumor suppressor role through recruitment of HDAC1 to transrepress the *HK2* gene.** XTT, cell proliferation, and Boyden chamber assays along with immunocytofluorescence and confocal microscopy were performed in vitro. Either *SLC14A1* or *SLC14A1-NLS* (nuclear form) overexpression suppressed cell viability, proliferation, migration and invasion **(A)** and induced mitochondrial fusion** (B)** in UMUC3 cells compared to the *mock*. **(C, D)** Either *SLC14A1* or *SLC14A1-NLS* overexpression downregulated SLC2A1, HK2, PKM, LDHA/C, pMTOR(S2448), and pRPS6(S235) and upregulated PDHA1 and MFN2 protein levels in UMUC3 cells compared to the *mock*.** (E)** Subcutaneous injection of *SLC14A1-NLS*-overexpressing UMUC3 cells into NOD/SCID mice (*n* = 8) suppressed tumor growth compared to the *mock*.** (F, G)** Immunohistochemistry showed that SLC14A1, PDHA1 and MFN2 protein levels were notably upregulated, whereas pMTOR(S2448), pRPS6(S235), Ki-67, SLC2A1, HK2, PKM and LDHA/C protein levels were markedly downregulated in xenografts from *SLC14A1-NLS*-overexpressing UMUC3 cells, similar to those of xenografts from *SLC14A1*-overexpressing UMUC3 cells. **(H)** Quantitative RT-PCR showed that overexpression of the *SLC14A1-NLS* gene downregulated the *HK2* mRNA level. **(I)** Cotransfection of the pKM2L-PhHKII (region #1) and pGL4.54[luc2/TK] plasmids into stable *SLC14A1-NLS*-overexpressing UMUC3 cells along with the Dual-Glo® Luciferase Reporter Assay displayed that *HK2* promoter activity was downregulated compared to the *mock*. **(J)** Coimmunoprecipitation by probing anti-SLC14A1 antibody (αSLC14A1) and immunoblot with anti-SIN3A or anti-HDAC1 antibody confirmed that SLC14A1 interacts with SIN3A and HDAC1 in both RTCC1 and BFTC905 cells. **(K)** The TRANSFAC® database predicted three putative HDAC1-responsive elements in the promoter region of the *HK2* gene, and a quantitative ChIP assay validated that *SCL14A1-NLS* overexpression increased the SCL14A1-NLS binding folds at site 1, site 2 and site 3. All experiments were performed in triplicate, and the results are expressed as the mean ± SD. For the immunocytofluorescence, immunoblot and immunohistochemistry assays, representative images are shown. Actin, beta (ACTB) served as a loading control for immunoblot analysis. Statistical significance: *P < 0.05.

In addition to nuclear SLC14A1 being found in clinical specimens (Figure 1D), nuclear and membranous SLC14A1 were further detected in RTCC1 and BFTC905 cells (Supplementary Figure S11A, S11B). Stable overexpression of the *SLC14A-NLS* gene upregulated *SLC14A1* mRNA and nuclear SLC14A1 protein levels in J82 and UMUC3 cells (Supplementary Figure S11C, S11D) and promoted nuclear SLC14A1 protein expression compared to *SLC14A1*-overexpressing J82 cells (Supplementary Figure S11E). A series of in vitro experiments showed that, similar to wild-type SLC14A1, nuclear SLC14A1-NLS induced G_1_ cell cycle arrest and inhibited cell viability, cell proliferation, cell migration, cell invasion, HUVEC tube formation, glucose uptake, while it enhanced mitochondrial fusion in J82 and UMUC3 cells (Supplementary Figure S12A-S12G; Figure 6A, 6B). SLC14A1-NLS regulated proteins related to DNA synthesis, glycolysis, mitochondrial respiration and mTOR signaling pathways in the same patterns as those of wild-type SLC14A1 in J82 and UMUC3 cells and xenografts from *SLC14A1*- and *SLC14A1-NLS*-overexpressing UMUC3 cells (Supplementary Figure S12H-S12K; Figure 6C, 6D). The average tumor size of xenografts from *SLC14A-NLS*- was similar to that of *SLC14A1*-overexpressing UMUC3 cells and much smaller than that of the *mock* (*P* < 0.01; Figure 6E). The expression levels of several proteins involved in glycolysis, mitochondrial respiration and mTOR signaling were analogous between xenografts from *SLC14A1-NLS*- and *SLC14A1*-overexpressing UMUC3 cells compared to the *mock* (Figure 6F, 6G).

To evaluate whether nuclear SLC14A1 regulates any genes involved in glycolysis, mitochondrial respiration or the mTOR signaling pathway, the expression levels of several transcripts were evaluated. Overexpression of the *SLC14A1-NLS* gene downregulated *SLC2A1*, *HK2*, and *LDHA* mRNA levels in J82 and UMUC3 cells (*P* < 0.05, Supplementary Figure S13A, S13B; Figure 6H).

In particular, exogenous *SLC14A1-NLS*- overexpression downregulated *HK2* promoter activity in J82 and UMUC3 cells (*P* < 0.05, Supplementary Figure S13C; Figure 6I). Further data mining on two independent experimental series in the Gene Expression Omnibus (GEO, NCBI) identified that *CDC25B*, *DEGS1*, *CLIC4* and *COLGALT1* were negatively correlated with *SLC14A1* mRNA levels (*P* < 0.05, Supplementary Figure S13D). Among these, *DEGS1* mRNA levels and promoter activities were consistently downregulated in *SLC14A1-NLS*-overexpressing J82 and UMUC3 cells (Supplementary Figure S13E, S13F). Stable knockdown of the *DEGS1* gene with 2 distinct shRNA clones downregulated *DEGS1* mRNA (*P* < 0.05) and the corresponding protein levels (Supplementary Figure S13G) and decreased cell proliferation (Supplementary Figure S13H). These data suggested that SLC14A1 may suppress *HK2* and/or *DEGS1* transcription along with cell proliferation.

The lack of NLS in the SLC14A1 protein suggested that SLC14A1 may translocate into the nucleus through interaction with other protein(s). Three potential SLC14A1-interacted proteins, namely, ARID4B, SIN3A and SUDS3 were identified in UMUC3 and J82 cells (Supplementary Figure S14A), and their interactions were verified by coimmunoprecipitation in distinct cell lines (Supplementary Figure S14B). Among these, ARID4B and SUDS3 are components of the SIN3A/HDAC corepressor complex, which is essential for transcriptional repression 22. The interaction between SLC14A1 and SIN3A or HDAC1 protein was next confirmed in RTCC1 and BFTC905 cells (Figure 6J). Three and one HDAC1 responsive elements in the *HK2* and *DEGS1* promoter regions, respectively, were predicted (Supplementary Figure S14C). A quantitative chromatin immunoprecipitation assay further confirmed the binding of HDAC1 to putative HDAC1-responsive elements in the *HK2* promoter in J82 (Supplementary Figure S14D) and UMUC3 (Figure 6K) cells as well as the *DEGS1* promoter in both cell lines (Supplementary Figure S14E), suggesting that nuclear SLC14A1 recruits HDAC1 with the coordination of SIN3A, ARID4B and/or SUDS3 to transrepress *HK2* and *DEGS1* genes in UC-derived cells (Supplementary Figure S15).

## Discussion

In this study, we uncovered that the nuclear as well as membranous SLC14A1 proteins play tumor suppressive roles through several signaling pathways in UC in clinical specimens, in vitro and in vivo. We initially performed data mining on the GEO dataset and identified that the *SLC14A1* mRNA level is frequently and gradually downregulated during UC progression and further validated this finding in UTUC and UBUC specimens at both the mRNA and protein levels. Data mining on the TCGA portal in cases of invasive bladder cancer reinforced our observations.

Although SLC14A1 was originally identified as a transmembrane urea transporter, we found that SLC14A1 protein was also expressed in the nucleus and cytosol in UC specimens. Importantly, the fact that nuclear SLC14A1 protein gradually faded away in the invasive front of UC specimens promoted us to investigate the nuclear roles of SLC14A1. We found that nuclear SLC14A1 is able to recruit HDAC1/SIN3A complex to transrepress the *HK2* gene. Since the interactions between SLC14A1 and HDAC1 were weaker than those between SLC14A1 and SIN3A, this interaction might be indirect. Tumor cells including urothelial cancer cells rely upon a particular switch from mitochondrial respiration to aerobic glycolysis/the Warburg effect as a major energy source to maintain rapid cell proliferation 23. In the first step of most glucose metabolism pathways, HK2 phosphorylates glucose to produce glucose-6-phosphate. Indeed, a high HK2 level in cancer cells is the foundation of 2-[(18)F]Fluoro-2-deoxyglucose positron emission tomography (^18^FDG-PET) imaging technology for clinical tumor detection. A meta-analysis showed that high HK2 levels conferred a poor prognosis in solid tumors of the digestive system 24. HK2 promoted tumor growth in human glioblastoma multiforme 25 and was integral to pathogenesis of medulloblastoma 26. Clinical, in vitro and in vivo evidence strongly suggested that upregulation of HK2 in ovarian cancer was correlated with metastasis and poor survival and mediated migration, invasion and stemness via PTK2/MAPK1/3/MMP9/NANOG/SOX9 signaling cascades in vitro and in a nude mouse model 27. Conditional knockout mice showed that *Hk2* was required for tumor initiation and maintenance in *Kras*-driven lung cancer and *Erbb2*-driven breast cancer. Without any adverse effects, mice with lung tumors could be controlled by systematic *Hk2* deletion 28. Therefore, SLC14A1 may suppress cell proliferation, migration and invasion through inhibition of *HK2* transcription and subsequent translation.

Another SLC14A1-transrepressed gene is *DEGS1*, and its protein product catalyzes the final step of *de novo* biosynthesis from dihydroceramide to ceramides in the sphingolipid signaling pathway. Knockdown of the *DEGS1* gene using small inhibitor RNA led to the accumulation of endogenous dihydroceramide, reduced cell growth and induced G_1_ cell cycle arrest in SMS-KCNR neuroblastoma cells 29. In addition, cell proliferation was defective in *DEGS1-*knockout embryonic fibroblasts 30. Loss-of-function through pharmacological or genetic ablation of *DEGS1* in preadipocytes prevented adipogenesis, resulting in an increase in oxidative stress, cellular death and cell cycle arrest due to dihydroceramide upregulation 31. In mitochondria isolated from mouse brain, either ischemia-induced or exogenously added ceramide caused respiratory chain damage 32. Pathological dyslipidemia causes fat oversupply to tissues not for lipid storage and induces cellular dysfunctions (i.e., lipotoxicity) including disruption of mitochondrial metabolism in insulin-resistant heart 33. Accordingly, followed by suppression of *DEGS1* transcription and translation, SLC14A1 may also inhibit tumor growth/cell proliferation in UC-derived cells through hindering lipotoxicity.

Prominently, we found that SLC14A1 overexpression promotes mitochondrial fusion and inhibits glycolysis, which was supported by mitochondrial elongation, an increased OCR, decreased ECAR and glucose uptake, downregulation of several glycolysis-related enzymes including SLC2A1, HK2, PKM, enzymes for the final step of anaerobic glycolysis, and LDHA/C and upregulation of MFN2 and PDHA1 protein levels in vitro and/or in an animal model. These aspects were also supported by significant correlations between SLC14A1 and related protein levels in UC specimens. Indeed, mitochondria are dynamic organelles and constantly change owing to coordinated fission, fusion or movement with the microtubular structure 34. A dynamic balance between MFN-dependent mitochondrial fusion and DNM1L-mediated mitochondrial fission determines the dimension and morphology of mitochondria 35. Mitochondrial fusion allows mingling of these organelles within a cell, avoiding losses of indispensable constituents 36. Mitochondrial fission and MFN2 downregulation are often found in various cancer cells 37. PDHA1 belongs to the PDH complex, which is comprised of multiple copies of three enzymatic members. It catalyzes the overall conversion of pyruvate to acetyl-CoA and CO_2_. Of these, PDHA1 contains the E1 active site and plays a pivotal role in the function of the PDH complex. Furthermore, downregulation of protein levels involving the glycolytic pathway suggests that SLC14A1 disfavors glycolysis. Thus, earlier studies strongly fortified our findings, consistent with the development of novel glycolytic inhibitors as a new class of anticancer agents.

We verified that functional SLC14A1 maintains a normal urea cycle by the prevention of arginine and urea accumulation and downregulation of downstream polyamines in vitro. Increasing evidence substantiates that tumor cells reprogram metabolism to expand nitrogen and carbon usage for cell proliferation. In normal cells, enzymes involved in the urea cycle are dynamically expressed to adapt to cellular requirements. However, in cancer cells, expression levels of these enzymes are adjusted to exploit nitrogen for DNA and/or protein biosynthesis 38. Our in vitro and in vivo studies showed that dysfunctional SLC14A1 upregulated TYMS and DHFR proteins, which participate in *de novo* DNA synthesis and repair, providing another indication to support our conclusions.

Moreover, we identified that functional SLC14A1 sensitizes arginine deprivation treatment in an *ASS1*-deficient UC-derived cell line. Arginine is a nonessential amino acid in humans, and ASS1 catalyzes the penultimate step of the arginine biosynthetic pathway in the urea cycle. Arginine auxotrophy due to *ASS1* loss/mutation is one recurrence characteristic in human malignancies 39. Recently, *ASS1* negativity was detected in ~40% of bladder cancer, and a multivariate analysis indicated worse disease-specific and metastasis-free survival rates 40. Surprisingly, functional SLC14A1 sensitizes ADP-PEG20 treatment (arginine deprivation) in *ASS1*-deficient RTCC1 cells, strengthening the finding that SLC14A1 also prevents arginine accumulation in addition to urea. Thus, SLC14A1 is critical to maintain a normal urea cycle and metabolic homeostasis in the urothelial system.

In vitro and in the animal model, we identified that SLC14A1 downregulated pMTOR(S2448), which is an active component of mTORC1 41. The mTORC1 complex comprises MTOR kinase and several accessory proteins. Increasing evidence shows that several mechanisms including the PI3K/Akt/mTOR pathway affect cancer cell metabolism 42. However, manipulation of SLC14A1 levels was not able alter pAKT1(S473) protein abundance in vitro and in the animal model, excluding that SLC14A1 regulated MTOR through the PI3K/Akt signaling. These notations were also reinforced by significant correlations between SLC14A1 and related protein levels in UC specimens. Spatiotemporal regulation of mTORC1 at the lysosome level identified that mTORC1 is a key signaling hub coordinating nutrient status and cell growth 43. Arginine is able to disrupt the complex CASTOR1/GATOR2 by binding to CASTOR1 and activates mTORC1 in cells 44. In this study, activated mTORC1 next phosphorylates RPS6 in the kinase domain, which is involved in multiple pathways including ribosome biogenesis and protein synthesis. *RPS6* encodes the 70 kDa serine/threonine kinase and regulates the translation of a subset of mRNA with an oligopyrimidine fragment at the 5' untranslated region. These mRNAs represent ~20% of entire cellular mRNA and are central components for translation apparatus 45. Phosphorylated/active RPS6 protein, which plays a crucial and positive role in controlling the cell cycle, growth and survival has been well documented 46. Further, mTORC1 promotes glycolysis by increasing HK2 translation in prostate cancer cells 47. Thus, SLC14A1 directly transrepresses *HK2* and inhibits arginine/MTOR-mediated HK2 translation. Instead, *DEGS1* ablation activates the Akt/mTOR signaling pathway in mouse embryonic fibroblasts 30; no negative feedback loop has been reported so far. As shown by our data, SLC14A1 inhibits the mTOR signaling pathway by restraint of arginine rather than by PI3K/AKT signaling.

In clinical specimens and UC-derived cell lines, we unearthed that negative mutations in the coding DNA sequence in the *SLC14A1* gene and both hypermethylation and histone/lysine methylation in CpG island of the *SLC14A1* promoter region cause low transcriptional and subsequent translational activities. Truly, epigenetics catalyzes diverse biochemical modifications to either the DNA or the histone proteins to adjust chromatin conformation, which further regulates gene expression temporally and spatially. Akin to most human malignancies, urological cancers are characterized by extensive epigenetic changes, especially histone methylation in euchromatin, resulting in the silencing of tumor suppressor genes and genomic instability 48, such as the *SLC14A1* gene in this study. The mRNA and protein levels of the *EZH2* gene, which encodes a polycomb-group protein catalyzing H3K27me2/3, were upregulated in muscle-invasive urothelial carcinoma 49. As a predominant methyltransferase, EHMT2 is required for overall mono- and dimethylation of H3K 50. Overexpression of EHMT2 in distinct carcinomas including esophageal squamous cell carcinoma, hepatocellular carcinoma, aggressive lung cancer, brain cancer, aggressive ovarian cancer and multiple myeloma were observed 51. The first approval of an EZH2 inhibitor, Tazverik™, by the USA FDA 52 and the rapidly development of EHMT2 inhibitors 53 as anticancer agents are consistent with our in vitro observations using EZH2 and EHMT2 inhibitors and knockdown of *EZH2* and *EHMT2* genes. Although the SLC14A1 protein was frequently lowly expressed in several and advanced cancers, this is the first study to identify that epigenetic silencing contributes to its low transcription and subsequent low translation.

Taken together, we identified that low SLC14A1 is a poor prognostic factor for disease-specific and metastasis-free survival in UTUC and UBUC patients. Epigenetic modifications including DNA hypermethylation and H3K27 and H3K9 histone methylations on the *SLC14A1* promoter resulted in low *SLC14A1* transcription and subsequent translation. In vitro, xenograft and/or tail vein injection experiments in mouse models provided evidence that total and membranous SLC14A1 inhibited cell viability, proliferation, migration, invasion, ECAR, tumor growth and metastasis, induced mitochondrial fusion, increased the OCR along with upregulation of proteins related to mitochondrial respiration and downregulated proteins associated with aerobic glycolysis, which may be mediated by downregulation of arginine and phosphorylated/active MTOR and RPS6. Functional SLC14A1 further sensitized arginine deprivation therapy in *ASS1*-deficient cells. Nuclear SLC14A1 recruited HDAC1 to transrepress *HK2* and *DEGS1* genes to maintain metabolic homeostasis.

### Availability of supporting data

All data generated or analyzed during this study are included in this published article and its supplementary documentation file.

## Supplementary Material

Supplementary materials and methods, figures, tables.

## Figures and Tables

**Table 1:** Univariate log-rank and multivariate analyses for disease-specific and metastasis-free survival in upper urinary tract urothelial carcinomas (UTUCs)

Parameter	Category	*n*	Disease-specific survival						Metastasis-free survival			
			Univariate analysis		Multivariate analysis				Univariate analysis	Multivariate analysis		
			*n*	*P* value	^A^R.R.	95% ^B^C.I.	*P* value	*n*	*P* value	^A^R.R.	95% ^B^C.I.	*P* value
Gender	Male	158	28	0.8286	-	-	-	32	0.7904	-	-	-
	Female	182	33		-	-	-	38		-	-	-
Age (years)	< 65	138	26	0.9943	-	-	-	30	0.8470	-	-	-
	≥ 65	202	35		-	-	-	40		-	-	-
Tumor side	Right	177	34	0.7366	-	-	-	38	0.3074	-	-	-
	Left	154	26		-	-	-	32		-	-	-
	Bilateral	9	1		-	-	-	0		-	-	-
Tumor location	Renal pelvis	141	24	0.0079*	1	-	0.666	31	0.0659	1	-	0.379
	Ureter	150	22		0.782	0.420-1.455		25		1.642	0.510-5.293	
	Renal pelvis & ureter	49	15		1.095	0.302-3.970		14		1.393	0.430-4.516	
Multifocality	Single	273	48	0.0026*	1	-	0.003*	52	0.0127*	1	-	0.025*
	Multifocal	62	18		3.391	1.538-7.486		18		3.313	1.163-9.439	
Primary tumor (T)	Ta	89	2	< 0.0001*	1	-	0.072	4	< 0.0001*	1	-	0.583
	T1	92	9		2.255	0.456-11.452		15		1.791	0.551-5.824	
	T2-T4	159	50		3.679	0.754-17.958		51		1.666	0.479-5.796	
Nodal metastasis	Negative (N0)	312	42	< 0.0001*	1	-	< 0.001*	55	< 0.0001*	1	-	< 0.001*
	Positive (N1-N2)	28	19		5.252	2.800-9.851		15		3.149	1.669-5.940	
Histological grade	Low grade	56	4	0.0215*	1	-	0.062	3	0.0027*	1	-	0.036*
	High grade	284	57		2.892	0.950-8.806		67		3.679	1.087-12.456	
Vascular invasion	Absent	234	24	< 0.0001*	1	-	0.100	26	< 0.0001*	1	-	0.002*
	Present	106	37		1.665	0.906-3.058		44		2.678	1.429-5.017	
Perineural invasion	Absent	321	50	< 0.0001*	1	-	0.001*	61	< 0.0001*	1	-	0.015*
	Present	19	11		3.567	1.707-7.454		9		2.572	1.197-5.527	
Mitotic rate (per 10 high power fields)	< 10	173	27	0.167	-	-	-	30	**0.0823**	1	-	0.521
	≥ 10	167	34		-	-	-	40		0.851	0.520-1.393	
SLC14A1 level	High	170	11	< 0.0001*	1	-	0.033*	16	< 0.0001*	1	-	0.012*
	Low	170	50		2.182	1.065-4.470		54		2.252	1.195-4.246	

^A^Relative Ratio (R.R.); ^B^Confidence Interval (C.I.); *Statistically significant

**Table 2:** Univariate log-rank and multivariate analyses for disease-specific and metastasis-free survival in urinary bladder urothelial carcinomas (UBUCs)

Parameter	Category	*n*	Disease-specific Survival					Metastasis-free Survival				
			Univariate analysis		Multivariate analysis			Univariate analysis		Multivariate analysis		
			*n*	*P* value	^A^R.R.	95% ^B^C.I.	*P* value	*n*	*P* value	^A^R.R.	95% ^B^C.I.	*P* value
Gender	Male	216	41	0.4446	-	-	-	60	0.2720	-	-	-
	Female	79	11		-	-	-	16		-	-	-
Age (years)	< 65	121	17	0.1136	-	-	-	31	0.6875	-	-	-
	≥ 65	174	35		-	-	-	45		-	-	-
Primary tumor (T)	Ta	84	1	< 0.0001*	1	-	< 0.001*	4	< 0.0001*	1	-	0.010*
	T1	88	9		4.960	0.547-44.980		23		4.574	1.333-15.701	
	T2-T4	123	42		14.978	1.697-132.221		49		5.892	1.684-20.624	
Nodal metastasis	Negative (N0)	266	41	0.0002*	1	-	0.498	61	< 0.0001*	1	-	0.040*
	Positive (N1-N2)	29	11		1.275	0.632-2.570		15		1.915	1.031-3.556	
Histological grade	Low grade	56	2	0.0013*	1	-	0.998	5	0.0007*	1	-	0.710
	High grade	239	50		1.002	0.219-4.579		71		0.809	0.275-2.383	
Vascular invasion	Absent	246	37	0.0024*	1	-	0.063	54	0.0001*	1	-	0.820
	Present	49	15		0.521	0.262-1.035		22		0.931	0.502-1.726	
Perineural invasion	Absent	275	44	0.0001*	1	-	0.023*	66	0.0007*	1	-	0.107
	Present	20	8		2.650	1.143-6.144		10		1.845	0.876-3.884	
Mitotic rate (per 10 high power fields)	< 10	139	12	< 0.0001*	1	-	0.016*	23	< 0.0001*	1	-	0.031*
	≥ 10	156	40		2.269	1.164-4.422		53		1.764	1.054-2.954	
SLC14A1 level	High	147	5	< 0.0001*	1	-	0.005*	16	< 0.0001*	1	-	0.031*
	Low	148	47		3.972	1.502-10.505		60		1.961	1.063-3.618	

^A^Relative Ratio (R.R); ^B^Confidence Interval (C.I.); *Statistically significant

## References

[1] Rosenberg JE, Hoffman-Censits J, Powles T, van der Heijden MS, Balar AV, Necchi A (2016). Atezolizumab in patients with locally advanced and metastatic urothelial carcinoma who have progressed following treatment with platinum-based chemotherapy: a single-arm, multicentre, phase 2 trial. *Lancet (London, England)*.

[2] Audenet F, Isharwal S, Cha EK, Donoghue MTA, Drill E, Ostrovnaya I (2019). Clonal relatedness and mutational differences between upper tract and bladder urothelial carcinoma. *Clinical Cancer Research*.

[3] Yang M-H, Chen K-K, Yen C-C, Wang W-S, Chang Y-H, Huang WJ-S (2002). Unusually high incidence of upper urinary tract urothelial carcinoma in Taiwan. *Urology*.

[4] Li N, Yang L, Zhang Y, Zhao P, Zheng T, Dai M (2011). Human Papillomavirus Infection and Bladder Cancer Risk: A Meta-analysis. *The Journal of Infectious Diseases*.

[5] Colin P, Koenig P, Ouzzane A, Berthon N, Villers A, Biserte J (2009). Environmental factors involved in carcinogenesis of urothelial cell carcinomas of the upper urinary tract. *BJU Int*.

[6] Gui Y, Guo G, Huang Y, Hu X, Tang A, Gao S (2011). Frequent mutations of chromatin remodeling genes in transitional cell carcinoma of the bladder. *Nature genetics*.

[7] Lucien N, Sidoux-Walter F, Olivès B, Moulds J, Le Pennec PY, Cartron JP (1998). Characterization of the gene encoding the human Kidd blood group/urea transporter protein. Evidence for splice site mutations in Jknull individuals. *J Biol Chem*.

[8] Shayakul C, Clémençon B, Hediger MA (2013). The urea transporter family (SLC14): Physiological, pathological and structural aspects. *Molecular Aspects of Medicine*.

[9] Dong Z, Ran J, Zhou H, Chen J, Lei T, Wang W (2013). Urea Transporter UT-B Deletion Induces DNA Damage and Apoptosis in Mouse Bladder Urothelium. *PLOS ONE*.

[10] de Maturana EL, Rava M, Anumudu C, Saez O, Alonso D, Malats N (2018). Bladder Cancer Genetic Susceptibility. A Systematic Review. *Bladder Cancer*.

[11] Frullanti E, Colombo F, Falvella FS, Galvan A, Noci S, De Cecco L (2012). Association of lung adenocarcinoma clinical stage with gene expression pattern in noninvolved lung tissue. *Int J Cancer*.

[12] Vaarala MH, Hirvikoski P, Kauppila S, Paavonen TK (2012). Identification of androgen-regulated genes in human prostate. *Molecular medicine reports*.

[13] Hou R, Kong X, Yang B, Xie Y, Chen G (2017). SLC14A1: a novel target for human urothelial cancer. *Clinical & translational oncology: official publication of the Federation of Spanish Oncology Societies and of the National Cancer Institute of Mexico*.

[14] Wu WR, Lin JT, Pan CT, Chan TC, Liu CL, Wu WJ (2020). Amplification-driven BCL6-suppressed cytostasis is mediated by transrepression of FOXO3 and post-translational modifications of FOXO3 in urinary bladder urothelial carcinoma. *Theranostics*.

[15] Chiang LC, Chiang W, Chang LL, Wu WJ, Huang CH (1996). Characterization of a new human transitional cell carcinoma cell line from the renal pelvis, RTCC-1/KMC. *Kaohsiung J Med Sci*.

[16] Li LC, Dahiya R (2002). MethPrimer: designing primers for methylation PCRs. *Bioinformatics*.

[17] Li CF, Wu WR, Chan TC, Wang YH, Chen LR, Wu WJ (2017). Transmembrane and Coiled-Coil Domain 1 Impairs the AKT Signaling Pathway in Urinary Bladder Urothelial Carcinoma: A Characterization of a Tumor Suppressor. *Clin Cancer Res*.

[18] Cheng CT, Kuo CY, Ouyang C, Li CF, Chung Y, Chan DC (2016). Metabolic Stress-Induced Phosphorylation of KAP1 Ser473 Blocks Mitochondrial Fusion in Breast Cancer Cells. *Cancer Res*.

[19] Lucien N, Sidoux-Walter F, Roudier N, Ripoche P, Huet M, Trinh-Trang-Tan M-M Antigenic and Functional Properties of the Human Red Blood Cell Urea Transporter hUT-B1. 2002;277(37):34101-8.

[20] Halaris A, Plietz J (2007). Agmatine: metabolic pathway and spectrum of activity in brain. *CNS Drugs*.

[21] Carroll B, Maetzel D, Maddocks OD, Otten G, Ratcliff M, Smith GR (2016). Control of TSC2-Rheb signaling axis by arginine regulates mTORC1 activity. *eLife*.

[22] Fleischer TC, Yun UJ, Ayer DE (2003). Identification and characterization of three new components of the mSin3A corepressor complex. *Mol Cell Biol*.

[23] Liberti MV, Locasale JW (2016). The Warburg Effect: How Does it Benefit Cancer Cells?. *Trends Biochem Sci*.

[24] Wu J, Hu L, Wu F, Zou L, He T (2017). Poor prognosis of hexokinase 2 overexpression in solid tumors of digestive system: a meta-analysis. *Oncotarget*.

[25] Wolf A, Agnihotri S, Micallef J, Mukherjee J, Sabha N, Cairns R (2011). Hexokinase 2 is a key mediator of aerobic glycolysis and promotes tumor growth in human glioblastoma multiforme. *J Exp Med*.

[26] Gershon TR, Crowther AJ, Tikunov A, Garcia I, Annis R, Yuan H (2013). Hexokinase-2-mediated aerobic glycolysis is integral to cerebellar neurogenesis and pathogenesis of medulloblastoma. *Cancer Metab*.

[27] Siu MKY, Jiang YX, Wang JJ, Leung THY, Han CY, Tsang BK (2019). Hexokinase 2 Regulates Ovarian Cancer Cell Migration, Invasion and Stemness via FAK/ERK1/2/MMP9/NANOG/SOX9 Signaling Cascades. *Cancers (Basel)*.

[28] Patra KC, Wang Q, Bhaskar PT, Miller L, Wang Z, Wheaton W (2013). Hexokinase 2 is required for tumor initiation and maintenance and its systemic deletion is therapeutic in mouse models of cancer. *Cancer Cell*.

[29] Kraveka JM, Li L, Szulc ZM, Bielawski J, Ogretmen B, Hannun YA (2007). Involvement of dihydroceramide desaturase in cell cycle progression in human neuroblastoma cells. *J Biol Chem*.

[30] Siddique MM, Li Y, Wang L, Ching J, Mal M, Ilkayeva O (2013). Ablation of dihydroceramide desaturase 1, a therapeutic target for the treatment of metabolic diseases, simultaneously stimulates anabolic and catabolic signaling. *Mol Cell Biol*.

[31] Barbarroja N, Rodriguez-Cuenca S, Nygren H, Camargo A, Pirraco A, Relat J (2015). Increased dihydroceramide/ceramide ratio mediated by defective expression of degs1 impairs adipocyte differentiation and function. *Diabetes*.

[32] Yu J, Novgorodov SA, Chudakova D, Zhu H, Bielawska A, Bielawski J (2007). JNK3 signaling pathway activates ceramide synthase leading to mitochondrial dysfunction. *J Biol Chem*.

[33] Makrecka-Kuka M, Liepinsh E, Murray AJ, Lemieux H, Dambrova M, Tepp K Altered mitochondrial metabolism in the insulin-resistant heart. *Acta Physiol (Oxf)* 2019:e13430.

[34] Youle RJ, van der Bliek AM (2012). Mitochondrial fission, fusion, and stress. *Science*.

[35] Santel A, Fuller MT (2001). Control of mitochondrial morphology by a human mitofusin. *J Cell Sci*.

[36] Chen H, Chan DC (2010). Physiological functions of mitochondrial fusion. *Ann N Y Acad Sci*.

[37] Senft D, Ronai ZA (2016). Regulators of mitochondrial dynamics in cancer. *Curr Opin Cell Biol*.

[38] Keshet R, Szlosarek P, Carracedo A, Erez A (2018). Rewiring urea cycle metabolism in cancer to support anabolism. *Nat Rev Cancer*.

[39] Riess C, Shokraie F, Classen CF, Kreikemeyer B, Fiedler T, Junghanss C (2018). Arginine-Depleting Enzymes - An Increasingly Recognized Treatment Strategy for Therapy-Refractory Malignancies. *Cell Physiol Biochem*.

[40] Allen MD, Luong P, Hudson C, Leyton J, Delage B, Ghazaly E (2014). Prognostic and therapeutic impact of argininosuccinate synthetase 1 control in bladder cancer as monitored longitudinally by PET imaging. *Cancer Res*.

[41] Dyachok J, Earnest S, Iturraran EN, Cobb MH, Ross EM (2016). Amino Acids Regulate mTORC1 by an Obligate Two-step Mechanism. *J Biol Chem*.

[42] Pelicano H, Martin DS, Xu RH, Huang P (2006). Glycolysis inhibition for anticancer treatment. *Oncogene*.

[43] Kim J, Guan KL (2019). mTOR as a central hub of nutrient signalling and cell growth. *Nat Cell Biol*.

[44] Chantranupong L, Scaria SM, Saxton RA, Gygi MP, Shen K, Wyant GA (2016). The CASTOR Proteins Are Arginine Sensors for the mTORC1 Pathway. *Cell*.

[45] Hornstein E, Git A, Braunstein I, Avni D, Meyuhas O (1999). The expression of poly(A)-binding protein gene is translationally regulated in a growth-dependent fashion through a 5'-terminal oligopyrimidine tract motif. *J Biol Chem*.

[46] Bahrami BF, Ataie-Kachoie P, Pourgholami MH, Morris DL (2014). p70 Ribosomal protein S6 kinase (Rps6kb1): an update. *J Clin Pathol*.

[47] Wang L, Xiong H, Wu F, Zhang Y, Wang J, Zhao L (2014). Hexokinase 2-mediated Warburg effect is required for PTEN- and p53-deficiency-driven prostate cancer growth. *Cell Rep*.

[48] O'Rourke CJ, Knabben V, Bolton E, Moran D, Lynch T, Hollywood D (2013). Manipulating the epigenome for the treatment of urological malignancies. *Pharmacol Ther*.

[49] Lauss M, Aine M, Sjodahl G, Veerla S, Patschan O, Gudjonsson S (2012). DNA methylation analyses of urothelial carcinoma reveal distinct epigenetic subtypes and an association between gene copy number and methylation status. *Epigenetics*.

[50] Tachibana M, Sugimoto K, Nozaki M, Ueda J, Ohta T, Ohki M (2002). G9a histone methyltransferase plays a dominant role in euchromatic histone H3 lysine 9 methylation and is essential for early embryogenesis. *Genes Dev*.

[51] Casciello F, Windloch K, Gannon F, Lee JS (2015). Functional Role of G9a Histone Methyltransferase in Cancer. *Front Immunol*.

[52] Hoy SM (2020). Tazemetostat: First Approval. *Drugs*.

[53] Cao H, Li L, Yang D, Zeng L, Yewei X, Yu B (2019). Recent progress in histone methyltransferase (G9a) inhibitors as anticancer agents. *Eur J Med Chem*.

