# Supplementary material for: SLC14A1 prevents oncometabolite accumulation and recruits HDAC1 to transrepress oncometabolite genes in urothelial carcinoma
Source: Theranostics. 2020 Sep 23;10(25):11775–93. doi: 10.7150/thno.51655 (PMC7546005; doi:10.7150/thno.51655)
Supplement: Supplementary file 1 — Supplementary materials and methods, figures, tables. [file thnov10p11775s1.pdf]

## Supplementary documentation

## Supplementary Materials and Methods

### Data mining

To explore the clinical relevance of *SLC14A1* mRNA level in urothelial carcinoma (UC), data mining was initially performed on two datasets (GSE32894 & GSE31684) from the Gene Expression Omnibus (GEO), analysis on 308 and 93 urinary bladder urothelial carcinomas (UBUCs) using Illumina HumanHT-12 V3.0 Expression BeadChip (Sjodal et al. 2012; *Clin Cancer Res* 18:3377-86) and Affymetrix Human Genome U133 Plus 2.0 Array (Riester et al. 2012; *Clin Cancer Res* 18:1323-33), respectively. To computerize the expression level, raw files were imported into the Nexus Expression 3 software (BioDiscovery) as described earlier (Li et al. 2017; *Clin Cancer Res* 23:7650-7663). The relationship between *SLC14A1* mRNA level and its mutation and promoter methylation statuses of invasive bladder cancers in the TCGA database was investigated using the cBioPortal online plateform (http://cbioportal.org).

### Patients and tumor materials

Patients were underwent surgery with curative intent. Those with confirmed or suspected lymph node metastasis received regional lymph node dissection. Cisplatin-based postoperative adjuvant chemotherapy was performed in patients with pT3-pT4 status or nodal involvement. The histological diagnoses of UBUCs and UTUCs were confirmed in all cases based on the latest World Health Organization classification. Histologic grading was assigned on the basis of Edmonson-Steiner criteria, whereas tumor stages were determined according to the 7$^{th}$ edition of the American Joint Committee on Cancer system. Medical charts were reviewed for each patient to ascertain the accuracy of other pertinent clinicopathologic data. Follow-up information was available in all cases with a median period of 44.7 months (ranging 3.0-175.8) for UTUCs and 30.8 months (ranging 3.0-109.0) for UBUCs. Histological features of all cases were evaluated independently by two expert pathologists (CF Li & TJ Chen).

### Quantigene assay

A branched DNA (bDNA) hybridization was used to quantitate the mRNA abundance of housekeeping and target transcripts in tissue homogenates from formalin-fixed specimens (FFPE) (Knudsen et al. 2018; *J Mol Diagn* 10:169-76). Briefly, the oligonucleotide probes targeting *SLC14A1* and *GAPDH* were incubated with total RNA in a capture plate overnight at 55°C. The probes cooperatively hybridized to *SLC14A1* or *GAPDH* mRNA and capture probes bound to the plate. About 300 μL of wash buffer was applied for 3 runs to remove unbound debris. Signal amplification was next performed via sequential hybridization of the bDNA preamplifier, amplifier, and label-probe molecules. The dioxetane alkaline phosphatase substrate Lumiphos Plus was added to the reaction wells for detection with a Luminex Bio-Plex 100 system (Luminex, Austin, TX, USA). The readout of the *SLC14A1* mRNA was further normalized to the reference *GAPDH* mRNA level.

### Immunohistochemistry, hematoxylin and eosin staining

We used tissue samples from BioBank of Chi Mei Medical Center. As a rule, informed consent is required before collection of the samples. Paraffin-embedded blocks from human specimens or mouse xenografts were sectioned into 4-μm thick slices and positioned on pre-coated slides. To melt the paraffin, an oven with 65°C was used. Next, the slides were de-paraffinized with xylene twice and rehydrated with 100% ethanol twice, 95% ethanol twice, 75% ethanol once and distilled water once, 10 min for each step. Antigen retrieval procedure was performed by treatment with 10 mM citrate buffer (pH 6) in a microwave for 20 min. Peroxidase-Blocking Solution (Dako, Agilent, Santa Clara, CA, USA) was used to remove endogenous peroxidase from tumor slices. After three

washes with PBS, the slices were incubated with each primary antibody for 1 h at room temperature, followed by washing with PBS three times, incubated in peroxidase-conjugated secondary antibody reagent [REAL™ EnVision™/HRP, Rabbit/Mouse (ENV); Dako] for 30 min at room temperature and detection of immune-staining via incubation with EAL™ DAB+ Chromogen diluted in REAL™ Substrate Buffer (Dako). Hematoxylin counterstaining was used to detect the nuclear location, followed by a dehydration procedure with 75% ethanol, 95% ethanol and 100% ethanol, 5 min for each step. Finally, the slices were mounted and examined. Primary antibodies used in the immunohitochemistry assay are listed in **Table 1**. Staining outcomes were examined by two expert pathologists (CF Li & TJ Chen) in Chi Mei Medical Center (Taiwan). Hematoxylin and eosin (H&E) staining was used to examine the lung metastasis in animal model by tail vein injection of cells which carried different *SLC14A1* genotypes.

**Table 1.** Primary antibodies used for immunohistochemistry

| Symbol/antibody | Protein | Dilution/Cat./Company |
|---|---|---|
| DHFR | Dihydrofolate reductase | 1:100, #3531-1, Labome |
| HK2 | Hexokinase 2 | 1:50, #2867, Cell Signaling |
| Ki-67 (MKI67) | Marker of proliferation Ki-67 | 1:200, ab66155, Abcam |
| LDHA/C | Lactate dehydrogenase A/C | 1:50, #3558, Cell Signaling |
| MAP1LC3B | Microtubule associated protein 1 light chain 3 beta | 1:50, #2775, Cell Signaling |
| MFN2 | Mitofusion 2 | 1:100, WH0009927M3, Sigma |
| pAKT1(S473) | AKT serine/threonine kinase 1<br>Substrate of MTOR | 1:25, #4060, Cell Signaling |
| pEIF4EBP1(S65) | Eukaryotic translation initiation factor 4E-binding protein 1<br>Substrate of MTOR, MAPK1/3 | 1:50, #9644, Cell Signaling |
| pMTOR(S2448) | Mechanistic target of rapamycin kinase<br>Substrate of RPS6KB1 | 1:50, ab51044, Abcam |
| pRPS6(S235) | Ribosomal protein S6<br>Substrate of RPS6KA1 | 1:100, ab80158, Abcam |
| SLC14A1 | Solute carrier family 14 member 1 | 1:50, AV48116, Sigma |
| SLC2A1 | Solute carrier family 2 member 1 | 1:200, #12939, Cell Signaling |
| PDHA1 | Pyruvate dehydrogenase E1 subunit alpha 1 | 1:100, ab168397, Abcam |
| PKM | Pyruvate kinase M1/2 | 1:400, #4053, Cell Signaling |
| TYMS | Thymidylate synthetase | 1:50, ab108995, Abcam |

**Cell culture**

Cell culture media, amino acid, antibiotics and serum were all purchased from ThermoFIsher (Waltham, MA, USA). J82 cells were incubated in DMEM containing 10% FBS. UMUC3 cells were cultured in DMEM supplemented with 10% FBS, 1 X L-glutamine. BFTC905 cells were maintained in DMEM supplemented with 10% Calf Serum (CS, #26010074, ThermoFisher). The RTCC1 cell line was cultured in RPMI 1640 medium with 10% CS and 1X L-glutamine. All cell lines were cultured in a humidified incubator with 5% $CO_2$ at 37°C.

**Chemicals, plasmids, mitochondrial fussion and fission**

5-aza-2′-deoxycytidine (5-Aza, Sigma-Aldrich, St. Louis, MI, USA), 3-deazaneplanocin A (DZNeP, Sigma-Aldrich), UNC0638 (Biovision, Milpitas, CA, USA), α-ketoglutaric acid (AKG, Sigma-Aldrich) were obtained. Plasmids including pLVX-puro-6HIS_v1, pLVX-puro-6HIS-*SLC14A1*_v1, pLVX-puro-6HIS-*SLC14A1*-NLS_v1 and pLVX-puro-6HIS-*SLC14A1(C25SC30S)*_v1 were reconstructed from the pLVX-Puro vector and pLenti-GIII-CMV-*SLC14A1*-GFP-2A-Puro plasmid (abm Inc., Vancouver, Canada). The pLV-mitoDsRed plasmid was purchased from Addgene (Watertown, MA, USA). Mitochondrial morphology was scored by the following criteria. Fragmented: more than 70% of mitochondria are small and round (< 3 μm); intermediated: mixture

of globular and shorter tabulated mitochondria (3 to 5 μm); and tabulated: more than 70% of mitochondrial are filamentous (> 5 μm); scale bar: 10 μm, based on our previous study (Cheng et al. 2016; *Cancer Res* 76:5006-5018). The *HK2* promoter reporter (HPRM30172-LvPG04) for UMUC3 cells was ordered from Genecopoeia (Rockville, MD, USA). Another *HK2* promoter reporter for J82 cells (pKM2L-phHKII, RDB05882) was obtained from RIKEN BRC (Ibaraki, Japan). The pGL4.54[luc2/TK] Vector (E5061) was purchased from Promega (Madison, WI, USA). *E. coli* embracing small hairpin RNA (shRNA) plasmids targeting specific human genes are listed in **Table 2** and were obtained from the National RNAi Core Facility (Institute of Molecular Biology/Genomic Research Center, Academia Sinica, Taipei, Taiwan).

**Table 2.** Clones used to stable knockdown specific human genes in this study

| Clone ID | RefSeq ID | Targeting region | Gene[3] | NCBI Gene ID |
|---|---|---|---|---|
| TRCN0000043608 | NM_015865 | CDS[1] | *SLC14A1* | 6563 |
| TRCN0000043609 | NM_015865 | CDS | *SLC14A1* | 6563 |
| TRCN0000431111 | NM_015865 | CDS | *SLC14A1* | 6563 |
| TRCN0000010475 | NM_004456 | 3'-UTR[2] | *EZH2* | 2146 |
| TRCN0000018365 | NM_004456 | CDS | *EZH2* | 2146 |
| TRCN0000040073 | NM_004456 | 3'-UTR | *EZH2* | 2146 |
| TRCN0000115667 | NM_025256 | 3'-UTR | *EHMT2* | 10919 |
| TRCN0000115668 | NM_025256 | CDS | *EHMT2* | 10919 |
| TRCN0000115669 | NM_025256 | CDS | *EHMT2* | 10919 |
| TRCN0000078514 | NM_052998 | CDS | *AZIN2* | 113451 |
| TRCN0000078516 | NM_052998 | CDS | *AZIN2* | 113451 |
| TRCN0000078517 | NM_052998 | CDS | *AZIN2* | 113451 |

[1]CDS: coding DNA sequence; [2]3'-UTR: 3'-untranslated region; *SLC14A1*: solute carrier family 14 member 1 (Kidd blood group); *EZH2*: enhancer of zeste 2 polycomb repressive complex 2 subunit; *EHMT2*: euchromatic histone lysine methyltransferase 2; *AZIN2*: antizyme inhibitor 2.

**Plasmid isolation**

The Wizard® Plus SV Minipreps DNA Purification System (Promega) was used for plasmid isolation. Bacterial pellets were suspended in Cell Resuspension Solution. Cells were lysed by adding Cell Lysis Solution, followed by Alkaline Protease Solution to remove endotoxins. Neutralization Solution was next joined to the mixture and vortexed thoroughly. The supernatant was retrieved after centrifugation at 15000 × g and next transferred into the spin column with a collection tube. After centrifugation, the waste inside the collection tube was discarded. The spin column containing plasmid was washed with Wash Solution. Finally, the spin column was moved to a fresh 1.5 mL microcentrifuge tube and Nuclease-Free Water was added into the central part of the column. The plasmid solution was harvested after centrifugation at 16, 000 × g for 1min.

**Preparation of viral particles and stable overexpression/knockdown of specific genes in UC-derived cells**

Phoenix-AMPHO cells (ATCC, Manassas, VA, USA) were transfected with the mixture containing plasmid with specific gene, psPAX2, PMD2.G and PolyJet™ reagent (SignaGen® Laboratories, Gaithersburg, MD, USA) diluted in DMEM medium. After transfection for 16 h, the medium was replaced with fresh ones. Supernatants containing viral particles were collected and purified using 0.45-μM PVDF Syringe Filters (Merck Millipore, Darmstadt, Germany). Cells were transduced with viral particles carrying genes of interest or shRNA targeting specific genes and stable clones were continually selected using 2 μg/mL of puromycin.

**RNA extraction and quantitative reverse transcription-polymerase chain reaction**

Total RNA was extracted using a Total RNA Purification Kit (GenMark Diagnostic, Carlsbad, CA, USA). Cell pellets were lysed with RNA Lysis/2-mercaptethanol (2-ME) Solution at room temperature. RNA was next precipitated using 70% of ethanol and incubated for 10 min at room temperature. Later, the mixture was transferred into an RNA spin column with a 2-mL collection tube and spun at the 15,000 × g for 1 min. The waste was discarded, RNA Wash Solution I was added and subjected to centrifugation. DNase I Incubation Buffer with DNase I was next added onto the spin column and incubated for 15 min at room temperature to avoid DNA contamination. Afterwards, RNA Wash Solution I was joined to the spin column and spun at 15,000 × g for 1 min. RNA Wash Solution II was dropped into the spin column with a collection tube and spun, repeated once more. The spin column was next transferred to a fresh collection and spun at 15,000 × g for one min to thoroughly remove the remaining solution around the column membrane. Nuclease-free water was added into the spin column in a fresh 1.5-mL microtube and incubated for one min, RNA solution was eluted after centrifugation. Purified RNA was subjected to cDNA synthesis using the Maxima First Strand cDNA Synthesis Kit for quantitative RT-PCR (ThermoFisher). Briefly, 20 µL of a reverse transcription mixture including RNA, 5X Reaction Mix, Maxima Enzyme Mix and nuclease-free water was incubated at 25°C for 10 min, followed by 30 min at 50°C and finally terminated at 85°C for 5 min.

Quantitative reverse-transcription polymerase chain reaction (qRT-PCR) was performed to measure the relative mRNA abundance using TaqMan™ Fast Advanced Master Mix, Pre-designed TaqMan assay reagents and a StepOne Plus System (Applied Biosystems, Waltham, MA, USA) according to the manufacturers' instructions. The protocol for thermal cycling were 95°C, 20 s (Taq activation); 95°C, 1 s (denaturation) and 60°C, 20 s (annealing and extension); a total of 40 cycles. Pre-designed TaqMan assay reagents from Applied Biosystems were *SLC14A1*: Hs00998197_m1; *EZH2*: Hs00544830_m1; *EHMT2*: Hs00198710_m1 and *POLR2A* (reference transcript): Hs00172187_m1. Other customized primers and probes, *HK2*: NM_000189.4; *LDHA*: NM_001135239.1; *LDHC*: NM_002301.4; *SLC2A1*: NM_006516.2 and *DEGS1*: NM_001321542.1 were ordered from Topgen (Taiwan). Relative mRNA levels were measured by the threshold cycle (CT) method. The equation $2^{-\Delta\Delta CT}$ was used to calculate the expression fold changes of target genes relative to the reference gene (*POLR2A*) in the experimental relative to the control group.

**Immunoblot analysis**

Exactly 30 µg of protein were separated on 4-12% gradient NuPAGE™ gels (Invitrogen, Carlsbad, CA, USA) and transferred onto polyvinylidene difluoride (PVDF) membranes (Merck Millipore). The membranes were blocked with 5% skim milk in Tris-buffered saline and polysorbate 20 (Tween 20) (TBST) buffer. Subsequently, the blots were incubated with individual primary antibody diluted in the TBST with 5% skim milk at 4°C overnight. After washing with TBST for three times, the blots were incubated with an appropriate secondary antibody [HRP Donkey anti-rabbit IgG [minimal x-reactivity, #406401, BioLegend® (San Diego, CA, USA)] or HRP goat anti-mouse IgG H&L (ab97023, Abcam, Cambridge, UK) diluted in the TBST with 5% skim milk at room temperature for 1 h. Blots were further washed in TBST for three times, and then the target proteins were visualized using the Pierce™ ECL western blotting Substrate or SuperSignal™ West Femto Maximum Sensitivity Substrate (ThermoFisher). The primary antibodies are listed in **Table 3**. ATPase Na+/K+ transporting subunit alpha 1 (ATP1A1) and Histone H2B type 1 (H2B) served as plasma membrane and nuclear markers.

**Table 3.** Primary antibodies and dilution for immunoblot analysis in this study

| Symbol/antibody | Protein | Dilution/Cat.#/Company |
|---|---|---|
| ACTB | Actin, beta | 1:3000, ab8226, Abcam |
| ATP1A1 | ATPase Na+/K+ transporting subunit alpha 1 | 1:5000, ab76020, Abcam |
| DHFR | Dihydrofolate reductase | 1:1000, #3531, Epitomics |

| | | |
|---|---|---|
| EIF4EBP1 | Eukaryotic translation initiation factor 4E binding protein 1 | 1:500, #9451, Cell Signaling |
| EHMT2 | Euchromatic histone lysine methyltransferase 2 | 1:1000, #3306, Cell Signaling |
| EZH2 | Enhancer of zeste 2 polycomb repressive complex 2 subunit | 1:1000, #5245, Cell Signaling |
| H2B | Histone H2B type 1 | 1:1000, ab1790, Abcam |
| HK2 | Hexokinase 2 | 1:1000, #2867, Cell Signaling |
| LDHA/C | Lactate dehydrogenase A/C | 1:1000, #3558, Cell Signaling |
| MAP1LC3B | Microtubule associated protein 1 light chain 3 beta | 1:1000, #2775, Cell Signaling |
| MFN2 | Mitofusin 2 | 1:1000, WH0009927M3, Sigma |
| MTOR | Mechanistic target of rapamycin kinase | 1:500, ab32028, Abcam |
| pAKT1(S473) | Phospho-AKT1 at serine 473 | 1:2000, #4060, Cell Signaling |
| Pan-AKT | AKT serine/threonine kinase | 1:2000, #4691, Cell Signaling |
| PDHA1 | Pyruvate dehydrogenase E1 subunit alpha 1 | 1:1000, #ab168379, Abcam |
| pEIF4EBP1(S65) | Phospho-EIF4EBP1 at serine 65 | 1:500, #9644, Cell Signaling |
| PKM | Pyruvate kinase M1/2 | 1:1000, #4053, Cell Signaling |
| pMTOR(S2448) | Phospho-MTOR at tyrosine 391 | 1:500, ab51044, Abcam |
| pRPS6(S235) | Phospho-RPS6 at serine 235 | 1:20000, ab80158, Abcam |
| RPS6 | Ribosomal protein S6 | 1:1000, ab137826, Abcam |
| SLC14A1 | Solute carrier family 14 member 1 (Kidd blood group) | 1:1000, AV48116, Sigma-Aldrich |
| SLC2A1 | Solute carrier family 2 member 1 | 1:1000, #12939, Cell Signaling |
| TYMS | Thymidylate synthetase | 1:1000, ab108995, Abcam |

**Next-generation sequencing assay on *SLC14A1* coding DNA sequence**

Genomic DNA from RTCC1, BFTC905, J82 and UMUC3 cell lines and FFPE tissues were extracted using the Total DNA Extraction Kit (Topgen, Taiwan) and FastPure FFPE DNA Isolation Kit (Vazyme, Nanjing, China), respectively. MultiNA MCE-202 with DNA-2500 Kit (Shimadzu, Kyoto, Japan) was applied to measure the concentration and length of extracted gDNA. Next, 50 ng of genomic DNA (gDNA) was used to construct a coding DNA sequence (CDS) library of the *SLC14A1* gene. Briefly, customized primer pools (Topgen), VAHTS AmpSeq Library Prep Kit V2 (NA201, Vazyme, Nanjing, China) and VAHTS Multiplex Oligo Set 4 (N321, Vazyme) for illumina were used to amplify exon 3-11 of the *SLC14A1* gene. The amplicon lengths ranged from 270 to 310 bp. Next, amplicons were purified using VAHTS DNA Clean Magnetic Beads (N411, Vazyme). The concentration and amplicon lengths were also determined using MultiNA MCE-202 with the DNA-2500 Kit. After sequencing, the bioinformatics analysis workflow including alignment between amplicons and Human Genome (GRCh38) was performed by Pear Paired-End Read Merger (Zhang et al. 2014; *Bioinformatics* 30:614-620; usegalaxy.org), Bowties2 (Langmead and Salzberg, 2012; *Nature Methods* 9:357-359; usegalaxy.org), Naïve Variant Caller (usegalaxy.org) and Variant Effect Predictor (McLaren et al. 2016; *Genome Biology* 17:122; Ensembl.org/vep) by Topgen Biotechnology (Taiwan).

**Bisulfite sequencing**

A CpG island in the promoter region of the *SLC14A1* gene was identified by MethPrimer CpG island prediction software (Li and Dahiya, 2002; *Bioinfomatics* 18:1427-31). Genomic DNAs from UTCC1, BFTC905, J82 and UMUC3 were extracted using the QIAamp DNA Mini Kit (Qiagen, Hilden, Germany). Briefly, cell pellets were suspended in Dulbecco’s phosphate-Buffered Saline (DPBS) and treated with Buffer AL. After mixing vigorously, Qiagen Protease K was added and incubated at 56°C for 10 min. Next, 100% of ethanol was used to precipitate genomic DNA and

transferred to the QIAamp Mini spin column with a collection tube and centrifuged at 15,000 × g for 1 min. The flowthrough was discarded and the spin column containing genomic DNA was washed using Buffer AW1 and centrifuged at 15,000 × g for 1 min. Afterward, the spin column was washed using Buffer AW2 and spun again. The spin column was subsequently placed in a fresh collection tube and centrifuged at 15,000 × g for 2 min. The spin column was subsequently positioned in a fresh 1.5-mL microcentrifuge tube. Distilled water was added to the central column membrane and incubated for 2 min. Finally, the spin column was spun at 16,000 × g for 1 min to elute the genomic DNA. Genomic DNA was subjected to bisulfite conversion via the EpiTect Fast DNA Bisulfite Kit (Qiagen), followed by the pyrosequencing assay (PyroMark Q24 system; Qiagen). Bisulfite sequencing for specimens of urothelial carcinomas was operated by MISSION BIOTECH (Taiwan). PCR and sequencing primers for pyrosequencing analysis are listed in **Table 4**.

**Table 4.** Primers for PCR and bisulfite sequencing in the CG-rich promoter region of the *SLC14A1* gene

| Primer | Sequence |
|---|---|
| PCR-forward | 5'-GTTATGTATTGAGAAAAAGTAAGGATGAA-3' |
| PCR-reverse | 5'-Biotin-ACCTATTCCTACCACCCATCTACCAA-3' |
| Sequencing | 5'-GAGAAAAAGTAAGGATGAAT-3' |

**Chromatin immunoprecipitation**

Briefly, paraformaldehyde (1% final concentration, 10 min) was used to cross-link histone and non-histone proteins to DNA. Glycine Solution (1X) was used to terminate the above reaction. Adherent cells were scratched and centrifuged. Cell pellets were suspended in 1X Buffer A containing dithiothreitol (DTT) and Protease Inhibitor Cocktail (PIC) and incubated on ice for 10 min, vortexed every 3 min. The mixture was centrifuged at 2,000 × g for 5 min at 4°C and the supernatant was removed. The pellet (nuclei) was resuspended in ice-cold 1X Buffer B with DTT and centrifuged at 2,000 × g for 5 min at 4°C. After removing the supernatant, nuclear pellets were resuspended in 1X Buffer B with DTT and chromatin was digested into 150- to 900-bp DNA/protein complexes by adding Micrococcal Nuclease for 20 min at 37°C and quenched using EDTA. Nuclear pellets were resuspended in 1X chromatin immunoprecipitation (ChIP) Buffer with PIC and incubated on ice for 10 min. After optimization the conditions: 5 kHz, 3 sets of 20-sec pulses with a 1/8-inch probe and on ice, a QSonica Q500 sonicator (M2 Scientifics, Holland, MI, USA) was applied to breakdown the nuclear membrane. The fragmented cross-linked chromatins were collected via centrifugation at 2,000 × g for 10 min (4°C). For ChIP assay, cross-linked chromatins were incubated with a specific antibody against H3K27me3, H3K9me2/me3 or HDAC1, followed by overnight incubation at 4°C in a lab rotator. The coprecipitates were next captured with protein G magnetic beads. The complex of precipitates/protein G was washed in a low-salt buffer for three times and a high-salt buffer once. The chromatins were eluted from the antibody/protein G complex by adding 1X ChIP Elution Buffer for 30 min at 65°C. By adding NaCl and proteinase, the chromatins were decrosslinked. Eluted DNA was analysed by quantitative RT-PCR (**Table 5**).

**Table 5.** Primers used for quantitative chromatin immunoprecipitation assays

| Primer | Sequence (5'→ 3") |
|---|---|
| CpG region in *SLC14A1* promoter-F | 5'-TCACATATTTTTGCCCTTTGTCAT-3' |
| CpG region 1 in *SLC14A1* promoter-R | 5'-TCACCAGCTTTAGTTCCAAAAGGG-3' |
| HDAC1-responsive element in *HK2* promoter-1F | 5'-AAGTGGGAGGACTGCTTGAGC-3' |
| HDAC1-responsive element in *HK2* promoter-1R | 5'-CATAATCCATCTAGCCTCTCAGCA-3' |
| HDAC1 binding site in *HK2* promoter -2F | 5'-CCTCGAACTCCTGGGCTCAAG-3' |

| | |
|---|---|
| HDAC1 binding site in *HK2* promoter -2R | 5'-GCCTGGGCGACATAGTGAGA-3' |
| HDAC1 binding site in *HK2* promoter -3F | 5'-AACCTTGGACTCCCAAAGTGCT-3' |
| HDAC1 binding site in *HK2* promoter -3R | 5'-CCATCCTCAAAACCACTGATAGG-3' |
| HDAC1 binding site in *DEGS1* promoter-F | 5'-CACTTGAGACCATTCCTTCCT-3' |
| HDAC1 binding site in *DEGS1* promoter-R | 5'-GTTCTGAGCTTCGGTGACTC-3' |

**Site-directed mutagenesis**

PCR-based technology and the QuikChange Lightning Site-Directed Mutagenesis Kit (Agilent) were used to generate a plasmid carrying a double mutation of the *SLC14A1* gene from cysteine (C) to serine (S) at residues 25 and 30, according to the manufacture's instruction. The pLVX-puro-6HIS-*SLC14A1*_v1 plasmid served as the first template to generate the pLVX-puro-6HIS-*SLC14A1(C25S)*_v1 plasmid by using C25S primers: 5'-GTGAAAACCAGGTTTCGCCATCTCAAGGGAGAAGGTGCTTCCCCA-3' and 5'-TGGGGAAGCACCTTCTCCCTTGAGATGGCGAAACCTGGTTTTCAC-3'). This plasmid was further used to construct pLVX-puro-6HIS-*SLC14A1(C25S/C30S)*_v1 plasmid using C30S primers: 5'-GCCATCTCAAGGGAGAAGGTCCTTCCCCAAAGCTCTTGGCTATGT-3' and 5'-ACATAGCCAAGAGCTTTGGGGAAGGACCTTCTCCCTTGAGATGGC-3'.

**Flow cytometric analysis**

Cells were harvested, followed by fixing via adding cold 70% of ethanol gradually to prevent aggregation and stored at -20°C for 24 h. Fixed cells were centrifuged to collect the pellet, washed and resuspended in PBS and stained with propidium iodide (PI)/ribonuclease (RNase) staining buffer (BD Biosciences, San Jose, CA, USA) for 15 min at room temperature in the dark. The cell cycle distribution was subsequently analysed using a Novocyte™ flow cytometer (ACEA Biosciences, San Diego, CA, USA) with the NovoExpress software built-in cell cycle analysis module (ACEA).

**Cell viability assay and cell proliferation assay**

Cell viability was analysed using the 2,3-Bis-(2-Methoxy-4-Nitro-5-Sulfophenyl) 2H-Tetrazolium-5-Carboxanilide (XTT) assay. Briefly, 1000-2000 cells (dependent on the cell size of each cell line) were seeded in 96-well microplates. After incubation for 24, 48 and 72 h, respectively, culture medium was replaced with the XTT and phenazine methosulfate (PMS) in phenol-free RPMI 1640 with a final concentration of 0.3 mg/mL and 20 μM, respectively. After incubating for 3 h, the absorbances were measured at a wavelength of 450 nm along with a reference of 600 nm (background value). The Cell Proliferation Assay Kit (Fluorometric, Biovision) was used to evaluate cell proliferation. Literally 1000-2000 cells were seeded in 96-well microplates. After incubation for 24, 48 and 72 h, respectively, 25 μL of reaction mixture containing 1X Nuclear Dye/Cell Lysis Buffer solution and 1X Nuclear Dye was added to each well. After appropriate incubation time period, the fluorescent intensity was measured on a microplate reader (GM3000, Promega) with excitation/emission: 480/538 nm.

**Cell migration and invasion assay**

The Boyden chamber assay was used to measure the capacity cell migration and cell invasion of cells. Falcon® Permeable Support for 24-well Plate with 8.0-μm Transparent Polyester (PET) Membrane inserts (#353097, Corning, Corning, NY, USA) and QCM ECMatrix Cell Invasion Assay, 24-well Plate with 8.0-μm (fluorimetric, #ECM554, Merck Millipore), respectively, were used for cell migration and invasion assay. Each insert was rehydrated with serum-free medium for 15 min and an appropriate number of cells suspended in serum-free medium were seeded in each insert and incubated with medium containing 10% serum in the bottom chamber. After incubation for 24 h (J82, UMUC3, and BFTC905 cells) or 48 h (RTCC1 cells), migration and invasion cells

passing through the inserts were detached and stained with the Lysis Buffer/Dye Solution followed by fluorescence detection on an ELISA reader with excitation/emission: 480/520 nm.

**Tube formation assay**

Human umbilical vein endothelial cells (HUVECs; ATCC) were used for the tube formation assay. Briefly, Matrigel® Basement Membrane Matrix (Corning) was dropped into the inner wells of a μ-Slide Angiogenesis (Ibidi, Grafelfing, Germany) and placed into a 37°C humidified incubator before HUVECs seeding. Next, HUVECs ($7 \times 10^3$) were suspended with supernatant (conditioned medium) from the indicated cultured cell lines and were seeded into each well. After 4 to 6 h, the images of tube formation in each well were captured by high-quality phase contrast microscopy.

**Ultra-performance liquid chromatography electrospray ionization-tandem mass spectrometry in multiple reactions monitoring**

To measure urea, arginine, L-ornithine, putrescine, spermidine and spermine concentrations in cells, ultra-performance liquid chromatography electrospray ionization-tandem mass spectrometry in multiple reactions monitoring mode (UPLC-MS-MRM) was performed in Core Facilities for Proteomics and Chemistry at National Health Research Institute, Taiwan. After gene manipulation, cells ($1 \times 10^7$) were collected and washed with PBS for three times. Cell pellets were suspended in 1 mL of cold methanol (100%) and subjected to liquid-nitrogen snap freezing for 1 h. Supernatants were next collected through centrifugation at 800 × g for 5 min and vacuum-dried. Samples were subjected to be analysed.

**Total, membranous and nuclear protein fractionation**

Total cell lysate was extracted using the PRO-PREP™ Protein Extraction Solution (iNtRON, Gyeonggi-do, Korea) containing Protease and Phosphatase Inhibitor Cocktail (ab20119, Abcam, Cambridge, UK). Cells were suspended in extraction solution and incubated on ice for 30 min. The total protein solution was collected by centrifugation at 15,000 × g for 15 minutes at 4°C. The Mem-PER™ Plus Membrane Protein Extraction Kit (#89842, ThermoFisher) was used to isolate membrane protein. Briefly, the cell pellet was washed twice with Cell Wash Solution and centrifuged at 300 × g for 5 min, resuspended in Permeabilization Buffer and incubated for 10 min at 4°C. Cytosolic proteins were separated by centrifugation at 16,000 × g for 10 min at 4°C. After removing the cytosolic extraction thoroughly, the pellet was suspended in Solubilization Buffer and incubated at 4°C for 30 min with vigorous vortex every 5 min. Membranous proteins were extracted via centrifugation at 16,000 × g for 15 minutes at 4°C.

Nuclear proteins were separated using the Nuclear/Cytosol Fractionation Kit (BioVision). Cell pellets were suspended in Cytosol Extraction Buffer A (CEB-A) containing Protease Inhibitors. After vigorous mixing and incubation for 10 min on ice, ice-cold CEB-B was added, mixed for 5 s and incubated for 1 min on ice. The supernatant (cytoplasmic fraction) was clearly removed after centrifugation at 16,000 × g and 4°C for 10 min. The cell pellet was resuspended in ice-cold Nuclear Extraction Buffer containing Protease Inhibitors and next admixed completely. The nuclear extract was collected after incubation for 40 min on ice and centrifugation at 16,000 × g and 4°C for 10 min.

**In vivo experiments (animal model)**

For xenograft model, *mock*-, *SLC14A1*-, *SLC14A1(C25SC30S)*- or *SLC14A1-NLS*-overexpressed UMUC3 cells ($5 \times 10^6$) were resuspended in 100 μL of cold PBS and mixed with 100 μL of high concentration Matrigel (Corning). The mixture was injected subcutaneously into 5-week-old NOD/SCID male mice ($n$ = 8 per group). Tumor size was measured with a digital caliper every other days. The tumor volume was calculated using the following formula: $V\ (mm)^3 = (\pi/6) \times width\ (mm)^2 \times length\ (mm)$. The mice were sacrificed at day 14 after injection. Solid tumors were

harvested and fixed in 10% formalin for paraffin embedded whole tissue sections. For metastatic evaluation, mice were tail vein injection with UMUC3 ($2 \times 10^6$) with *mock*-, *SLC14A1*- or *SLC14A1(C25S/C30S)*-overexpressed along with transfection of the pHIV-Luc-ZsGreen luciferase reporter (#39196, Addgene)'. Mice were anesthetized using isoflurane vaporizer and intraperitoneal injection with D-Luciferin, followed by detection of the luminescent signals using an In-Vivo Xtreme II system (Bruker, Billerica, MA, USA) every week and the X-ray image was captured to show the contour of the mice. Bioluminescent signals indicated the metastatic status after injection for 34 days. Mice were sacrificed and the lung were harvested, followed by fixing in 10% formalin for further immunohistochemistry.

**Immunocytofluorescence with confocal microscopy**

The appropriate number of cells (RTCC1, BFTC905, *mock*-, *SLC14A1*-, *SLC14A1-NLS*-carrying J82) was seed onto cover glass placed onto the 6-well cell culture dish. After incubation for 24 h, cells were fixed with 4 % paraformaldehyde for 20 min and permeabilized with 0.1% Triton X-100 for 10 min and washed with PBS. Cells were next treated with 1% BSA for 1 h and incubated with anti-human SLC14A1 (ab23872, Abcam) antibody at 4°C for 24 h, followed by secondary antibody conjugated with Alexa Fluor® 568 (ab175696, Abcam) at room temperature for 1 h and washed with PBS. In RTCC1, *mock*-, *SLC14A1*- and *SLC14A1-NLS*-carrying J82, cells were incubated with anti-cadherin 2 (CDH2) (ab6528, Abcam; plasma membrane marker) antibody for another 24 h, followed by an appropriate secondary antibody conjugated with Alexa Fluor® 488 (ab150113, Abcam) for 1 h. In BFTC905, cells were probed with recombinant ATPase $Na^+/K^+$ transporting subunit alpha 1 (ATP1A1) (plasma membrane marker, Alexa Flour® 488, ab197713, Abcam) antibody at 4°C for another 24 h. After double staining, cells were mounted with Aqueous Mounting Medium containing 4',6-diamidino-2-phenylindole (DAPI, Santa Cruz, Santa Cruz, CA, USA) and visualized with a confocal microscope to detect the immunostaining in cellular compartments.

**Oxygen consumption rate and extracellular acidification rate assays**

Cellular oxygen consumption rate (OCR) and extracellular acidification rate (ECAR) were measured using a Seahorse XFp analyser (Seahorse Bioscience, North Billerica, MA, USA) according to the manufacturer's instructions. Cells were seeded in an 8-well cell culture miniplate. The probe tip of the sensor cartridge was hydrated by adding Seahorse XF Calibrant Solution to the lower plate of the cartridge. Subsequently, the sensor cartridge was placed at a non-$CO_2$ 37°C incubator overnight. For the OCR assay, oligomycin, carbonyl cyanide-4-(trifluoromethoxy)phenylhydrazone (FCCP), rotenone and antimycin A (RA) were sequentially loaded into appropriate pores of the sensor cartridge. The sensor cartridge was next placed into the Seahorse XFp Extracellular Flux Analyzer to calibrate and equilibrate. At the same time, the cells seeded in the culture miniplate were treated with Seahorse XF base medium with glucose. Once the calibration and equilibration steps were finished, the lower plate of cartridge was replaced by a cell culture plate. For ECAR assay, glucose, oligomycin and 2-deoxy-D-glucose (2-DG) were sequentially loaded into appropriate pores of the sensor cartridge. The sensor cartridge was next placed into the Seahorse XFp Extracellular Flux Analyzer to calibrate and equilibrate. In the meantime, the cells seeded in the culture miniplate were treated with Seahorse XF base medium without glucose for 30 min in a non-$CO_2$ incubator at 37°C. Next, the lower plate of the cartridge was removed and replaced with a cell culture miniplate. The data for OCR and ECAR in each well were collected at each indicated time point (min) and were analysed by the Agilent Seahorse Wave Desktop software.

**Glucose uptake**

Based on the protocol of Glucose Uptake Colorimetric Assay Kit (#ab136955, Abcam), cells seeded in the 96 well plate were starved with serum- and glucose-free medium overnight. Cells were treated with Kerb-Ringer-Phosphate-HEPES (KRPH) buffer [HEPES (20 mM), $KH_2PO_4$ (5 mM),

$MgSO_4$ (1 mM), $CaCl_2$ (1 mM), NaCl (136 mM), KCl (4.7 mM); pH 7.4] supplemented with 2% BSA and insulin (1 μM) for 20 min to activate the glucose transporter. Subsequently, 10 µL of 2-Deoxy-D-Glucose (2-DG, 10 mM) was added and incubated for 20 min. After three rounds of washing with PBS, cells were suspended in 80 µL of Extraction Buffer and incubated at 85°C for 40 min to degrade the endogenous NAD(P). Through neutralization with Neutralizing Buffer, 5 µL of suspended cells were diluted in 45 µL of Assay Buffer (1:9) and mixed with 10 µL of Reaction Mix A (mixture: 8 μL of Assay Buffer and 2μL of Enzyme Mix), followed by an incubation at 37°C for 15 min to generate NADPH from 2-deoxy-D-glucose-6-phosphate (2DG6P). Thereafter, 90 µL of Extraction Buffer was added and heated to 90°C for 40 min to degrade remaining NAD(P). Finally, Reaction Mix B including 20 µL of Glutathione Reductase, 16 µL of Substrate and 2 µL of Recycling Mix was added into each sample and incubated at 37°C for 15 min. Absorbances were measured at a wavelength of 405 nm. Similar to glucose in structure, 2-DG was taken by glucose transporters, metabolized to 2DG6P and accumulated within cells. The gathered 2DG6P is directly proportional to 2-DG uptake by cells. The 2DG6P was oxidized to generate NADPH and determined by an enzymatic recycling amplification reaction (Supplementary).

**Nanoflow liquid chromatography-nano electrospray ionization-tandem mass spectrometry**

Dynabeads™ His-Tag Isolation & Pulldown assay Kit (#10103D, Invitrogen) was used to pulldown 6HIS-SLC14A1/protein complexes using metal affinity chromatography chemistry. Target bands in the SDS-PAGE gel were sliced and minced with wash buffer (10 mM ammonium bicarbonate in 50% acetonitrile). Proteins were reduced using 10 mM DTT/10 mM ammonium bicarbonate at 56°C for 15 min, followed by alkylation with 55 mM iodoacetamide and 10 mM ammonium bicarbonate at room temperature for 20 min in the dark. Trypsin digestion was performed overnight at 37°C. Next, 1% trifluoroacetic acid in 50% acetonitrile was used to extract peptides. Vacuum-dried samples were examined by nanoflow-liquid chromatography-nano electrospray ionization-tandem mass spectrometry (nano-LC-nanoESI MS/MS) analysis (Academia Sinica Common Mass Spectrometry Facilities, Taiwan) to identify the peptide identities using the MaxQuant software (Max Planck Institute of Biochemistry, Munchen, Germany).

**Coimmunoprecipitation**

Total protein was extracted from *SLC14A1*-overexpressed J82 and UMUC3. Appropriate amount of lysate was incubated with primary antibody against SLC14A1 at 4°C overnight. Sample/antibody mixture was incubated with pre-washed magnetic beads for 1 h at room temperature. The sample/antibody/beads admixture was next washed with immunoprecipitation (IP) Lysis/Wash Buffer and the protein complexes were eluted using Elution Buffer. Eluted immunocomplexes were separated by SDS-PAGE and detected by the indicated antibody against SIN3A (Proteintech, Rosemont, IL, USA) or HDAC1 (Cell Signaling, Danvers, MA, USA) or ARID4B (Proteintech) or SDS3 (Novus Biologicals, Centennial, CO, USA).

**Luciferase reporter assays**

Briefly, the reaction buffer was prepared by combining the Enzyme mix with Dual-Glo® Luciferase Reagent and added into a 96-well plate with cells in culture medium. The activity of firefly luciferase was detected using an ELISA reader. Afterwards, the firefly luciferase reaction in each well was quenched by adding Dual-Glo® Stop & Glo® Reagent and the activity of *Renilla* luciferase was also detected using an ELISA reader. *Renilla* activity was normalized to the Firefly activity for each sample.

Region #2 of the *HK2* promoter (HPRM30172-LvPG04, Lentiviral system, Genecopoeia) was cloned into the Dual-reporter vector containing the Gaussia luciferase (Gluc) reporter. The Secreted Alkaline Phosphatase (SEAP) reporter served as an internal control. The viral particles were produced as described previously. *Mock*- and *SLC14A1-NLS*-overexpressed UMUC3 cells were transduced with viral supernatant for 24 h and replaced with fresh medium for another 24 h. Culture

medium was next collected and analysed using The Secrete-Pair™ Dual Luminescence Assay (LF033, Genecopoeia). Briefly, Gluc Assay Working Solution was prepared by mixing substrate GL and 1X Buffer GL-S, followed by incubation for 25 min at room temperature. Afterward, 10 µL of culture media from the *mock*- and *SLC14A1-NLS*-overexpressed UMUC3 cells were added to a Corning® 96-well white plate and mixed with 100 µL GLuc Assay Working Solution, followed by incubation for 1 min at room temperature. The luminescence intensities catalysed by the Gaussia luciferase were measured using an ELISA reader. For the SEAP activity assay, the culture medium was heated at 65°C for 15 min and then placed on ice. The SEAP Assay Working Solution was prepared by mixing substrate alkaline phosphatase (AP) and 1X Buffer AP. Later, 10 µL of processed medium (sample) from *mock*- and *SLC14A1-NLS*-overexpressed UMUC3 cells was dropped into a 96-well white plate and mixed with 100 µL of SEAP Assay Working Solution. After incubation for 10 min at room temperature, the luminescence catalyzed by SEAP was assessed using an ELISA reader. Gluc was normalized to the SEAP activity for each sample. A similar system was used to detect the promoter activity of the *DEGS1* gene using *DEGS1* promoter (HPRM45067-LvPG04, Genecopoeia) and dual reporter system (LF033, Genecopoeia) in both J82 and UMUC3 cells.

**Statistics**

Statistical analysis was performed using SPSS software (IBM, Armonk, NY, USA). Two-tailed *t*-test was used to examine the significance of the relative mRNA levels, different phases of the cell cycle, percentages of cell viability, ability of cell migration/invasion and HUVEC tube formation. The Kaplan-Meier method was applied to estimate the effect of SLC14A1 protein level on patient outcomes. The survival curves were conducted using log-rank test. A Cox proportional-hazards model was used to identify independent predictors for disease-specific survival (DSS) and metastasis-free survival (MFS). For all statistics, $P < 0.05$ was considered as statistical significance.

**Table S1.** Data mining on the urothelial carcinoma transcriptome, GSE32894, identified a stepwise downregulation of *SLC14A1* transcript during the progression of urothelial carcinomas

| **Probe** | **T2-4 vs. Ta** | | **T1 vs. Ta** | | **T2-4 vs. T1** | | **Gene** | **Biological process** | **Molecular function** |
|---|---|---|---|---|---|---|---|---|---|
| | **log ratio** | ***P* value** | **log ratio** | ***P* value** | **log ratio** | ***P* value** | | | |
| ILMN_1805561 | -1.6149 | < 0.0001 | -0.6109 | 0.0003 | -1.0041 | < 0.0001 | ***SLC14A1*** | Transport, urea transport, water transport | Copper ion binding, ubiquitin-ubiquitin ligase activity, urea transmembrane transporter activity, water transporter activity |
| ILMN_2197659 | -0.6326 | < 0.0001 | -0.3288 | 0.0004 | -0.3037 | 0.0068 | ***SLC14A1*** | Transport, urea transport, water transport | Copper ion binding, ubiquitin-ubiquitin ligase activity, urea transmembrane transporter activity, water transporter activity |

**Table S2.** Data mining on the urothelial carcinoma transcriptome, GSE31684, identified downregulation of *SLC14A1* during the progression of urothelial carcinomas

| **Probe** | **T2-4 vs. Ta-T1** | | **Gene** | **Biological process** | **Molecular function** |
|---|---|---|---|---|---|
| | **log ratio** | ***P* value** | | | |
| 205856_at | -3.2088 | < 0.0001 | ***SLC14A1*** | Transport, urea transport, water transport | Copper ion binding, ubiquitin-ubiquitin ligase activity, urea transmembrane transporter activity, water transporter activity |
| 229151_at | -4.1652 | < 0.0001 | ***SLC14A1*** | Transport, urea transport, water transport | Copper ion binding, ubiquitin-ubiquitin ligase activity, urea transmembrane transporter activity, water transporter activity |

**Table S3.** Correlations between SLC14A1 protein levels and important clinicopathological parameters in urothelial carcinomas

| **Parameter** | **Category** | **Upper Tract Urothelial Carcinoma (UTUC)** | | | | **Urinary Bladder Urothelial Carcinoma (UBUC)** | | | |
|---|---|---|---|---|---|---|---|---|---|
| | | ***n*** | **SLC14A1 protein level** | | ***P* value** | ***n*** | **SLC14A1 protein level** | | ***P* value** |
| | | | **High** | **Low** | | | **High** | **Low** | |
| Gender | Male | 158 | 80 | 78 | 0.828 | 216 | 106 | 110 | 0.667 |
| | Female | 182 | 90 | 92 | | 79 | 41 | 38 | |
| Age (years) | < 65 | 138 | 71 | 67 | 0.659 | 121 | 60 | 61 | 0.994 |
| | ≥ 65 | 202 | 99 | 103 | | 174 | 87 | 87 | |
| Tumor location | Renal pelvis | 141 | 64 | 77 | 0.228 | - | - | - | - |
| | Ureter | 150 | 77 | 73 | | - | - | - | - |
| | Renal pelvis & ureter | 49 | 29 | 20 | | - | - | - | - |
| Multifocality | Single | 278 | 133 | 145 | 0.092 | - | - | - | - |
| | Multifocal | 62 | 37 | 25 | | - | - | - | - |
| Primary tumor (T) | Ta | 89 | 84 | 5 | **< 0.001*** | 84 | 72 | 12 | **< 0.001*** |
| | T1 | 92 | 42 | 50 | | 88 | 54 | 34 | |
| | T2-T4 | 159 | 44 | 115 | | 123 | 21 | 102 | |
| Nodal metastasis | Negative (N0) | 312 | 165 | 147 | **< 0.001*** | 266 | 143 | 123 | **< 0.001*** |
| | Positive (N1-N2) | 28 | 5 | 23 | | 29 | 4 | 25 | |
| Histological grade | Low grade | 56 | 45 | 11 | **< 0.001*** | 56 | 48 | 8 | **< 0.001*** |
| | High grade | 284 | 125 | 159 | | 239 | 99 | 140 | |
| Vascular invasion | Absent | 234 | 140 | 94 | **< 0.001*** | 246 | 142 | 104 | **< 0.001*** |
| | Present | 106 | 30 | 76 | | 49 | 5 | 44 | |
| Perineural invasion | Absent | 321 | 167 | 154 | **0.002*** | 275 | 142 | 133 | **0.021*** |
| | Present | 19 | 3 | 16 | | 20 | 5 | 15 | |
| Mitotic rate (per 10 high power fields) | < 10 | 173 | 100 | 73 | **0.003*** | 139 | 82 | 57 | **0.003*** |
| | ≥ 10 | 167 | 70 | 97 | | 156 | 65 | 91 | |

*Statistical significance

**Table S4.** Urothelial carcinoma-derived cell lines subjected to next-generation sequencing at coding DNA sequence in *SLC14A1* gene and quantitative DNA methylation analyses by pyrosequencing in the *SLC14A1* promoter region

| **Cell line** | **Age at sampling** | **Gender** | **Mutation** | **SLC14A1 protein level** | ***SLC14A1* promoter CpG region** | | | | |
|---|---|---|---|---|---|---|---|---|---|
| | | | | | **Position 1** | **Position 2** | **Position 3** | **Position 4** | **Position 5** |
| | | | | | **Methylation (%)** | **Methylation (%)** | **Methylation (%)** | **Methylation (%)** | **Methylation (%)** |
| RTCC1 | 65 | F | Negative | High | 2 | 3 | 1 | 3 | 3 |
| BFTC905 | 51 | F | Negative | High | 28 | 30 | 26 | 27 | 29 |
| J82 | 58 | M | Negative | Low | 35 | 84 | 82 | 71 | 86 |
| UMUC3 | Unspecified | M | Negative | Low | 1 | 81 | 88 | 79 | 98 |

**Table S5.** The clinicopathological parameters in patients with urothelial carcinoma subjected to next-generation sequencing at complete DNA sequence (CDS) in *SLC14A1* gene and quantitative DNA methylation analyses by pyrosequencing in the *SLC14A1* promoter region

| Case ID | Age | Gender | Grade | pT status | pN status | [1]VI | [2]NI | [3]MF/10 | Mutation | SLC14A1 level | ***SLC14A1* promoter CpG region** | | | | |
|---|---|---|---|---|---|---|---|---|---|---|---|---|---|---|---|
| | | | | | | | | | | | **Position 1** | **Position 2** | **Position 3** | **Position 4** | **Position 5** |
| | | | | | | | | | | | **Methylation (%)** | **Methylation (%)** | **Methylation (%)** | **Methylation (%)** | **Methylation (%)** |
| UC1 | 60 | M | High | T3 | Nx | Y | N | 16 | Negative | High | 39.94 | 82.86 | 54.5 | 84.3 | 86.69 |
| UC2 | 69 | F | High | T2 | N0 | Y | N | 12 | N/A | Low | 41.65 | 91.7 | 89.57 | 76.75 | 97.97 |
| UC3 | 53 | M | High | Ta | N0 | N | N | 8 | N/A | High | 1.31 | 90.46 | 93.24 | 95.83 | 100 |
| UC4 | 60 | M | High | T1 | N0 | N | N | 22 | Negative | High | 1.12 | 54.23 | 57.19 | 60.29 | 33.68 |
| UC5 | 73 | M | High | Ta | N0 | N | N | 2 | Negative | High | 0 | 1.8 | 3.44 | 95.16 | 2.63 |
| UC6 | 69 | F | High | T2 | N0 | Y | N | 3 | Negative | High | 0.69 | 86.28 | 93.35 | 84.81 | 98.79 |
| UC7 | 58 | M | Low | Ta | N0 | N | N | 5 | N/A | High | 11.76 | 12.59 | 1.71 | 13.8 | 2.68 |
| UC8 | 60 | M | High | T3 | N0 | N | N | 15 | N/A | Low | 52.24 | 78.11 | 74.07 | 79.35 | 81.57 |
| UC9 | 62 | M | High | T1 | N0 | N | N | 3 | N/A | High | 36.2 | 93.16 | 80.05 | 83.83 | 87.71 |
| UC10 | 70 | M | High | T3 | N0 | Y | N | 2 | Negative | Low | 67.39 | 85.56 | 69.24 | 72.16 | 92.25 |
| UC11 | 49 | F | High | T2 | N0 | N | N | 38 | N/A | Low | 77.72 | 76.58 | 64.15 | 79.36 | 82.6 |
| UC12 | 55 | M | High | T1 | N0 | N | N | 12 | N/A | High | 21.36 | 66.12 | 65.84 | 56.18 | 59.14 |
| UC13 | 59 | M | High | Ta | N0 | N | N | 10 | Negative | Low | 43.92 | 77.93 | 74.99 | 74.95 | 64.98 |
| UC14 | 53 | M | High | T3 | N0 | Y | Y | 36 | N/A | Low | 98.13 | 6.37 | 85.32 | 93.97 | 95.48 |
| UC15 | 70 | F | Low | Ta | N0 | N | N | 8 | N/A | High | 3.4 | 5.31 | 6.92 | 8.28 | 3.66 |
| UC16 | 77 | M | High | T2 | N0 | Y | N | 21 | Negative | Low | 97.58 | 98.32 | 87.71 | 94.5 | 79.17 |
| UC17 | 68 | F | High | T3 | N0 | Y | N | 15 | Negative | High | 0.57 | 88.56 | 90.57 | 75.44 | 95.71 |
| UC18 | 76 | M | High | Ta | N0 | N | N | 9 | Negative | High | 0.65 | 88.99 | 93.75 | 95.27 | 93.96 |
| UC19 | 61 | F | High | T1 | N0 | N | N | 13 | N/A | High | 25.24 | 78.52 | 86.11 | 79.55 | 100 |
| UC20 | 67 | M | High | Ta | N0 | N | N | 5 | N/A | High | 0.84 | 85.28 | 89.39 | 90.71 | 100 |
| UC21 | 66 | F | High | T3 | N0 | Y | N | 9 | Negative | High | 1.4 | 89.05 | 78.27 | 94.39 | 79.85 |
| UC22 | 68 | F | High | Ta | N0 | N | N | 1 | Negative | High | 0.6 | 53.66 | 73.63 | 75.84 | 77.1 |
| UC23 | 65 | M | High | T2 | N0 | N | N | 9 | N/A | Low | 75.99 | 25.48 | 87.17 | 75.28 | 96.33 |
| UC24 | 56 | F | High | T3 | N0 | Y | N | 9 | Negative | High | 30.13 | 91.57 | 91.17 | 94.32 | 100 |
| UC25 | 54 | M | High | T1 | N0 | N | N | 4 | N/A | High | 33.18 | 79.68 | 70.47 | 79.52 | 79.37 |
| UC26 | 67 | F | High | T2 | N0 | N | N | 8 | Negative | High | 27.68 | 60.45 | 91.9 | 95.23 | 63.88 |
| UC27 | 69 | M | High | Ta | N0 | N | N | 1 | Negative | High | 2.2 | 59.59 | 72.59 | 90.15 | 97.15 |
| UC28 | 62 | M | High | T3 | N0 | Y | Y | 40 | N/A | Low | 51.8 | 89.61 | 84.85 | 89.68 | 100 |
| UC29 | 77 | M | High | T4 | N1 | Y | Y | 27 | N/A | Low | 89.43 | 96.56 | 69.72 | 83.21 | 100 |
| UC30 | 60 | M | High | T2 | N1 | N | N | 9 | N/A | Low | 91.81 | 95.07 | 76.16 | 64.13 | 96.15 |
| UC31 | 65 | F | High | T2 | N0 | N | N | 17 | N/A | Low | 35.14 | 82.94 | 82.06 | 88.58 | 98.66 |
| UC32 | 66 | M | High | Ta | N0 | N | N | 3 | Negative | Low | 42.75 | 82.27 | 64.72 | 79.28 | 100 |

| | | | | | | | | | | | | | | | |
|---|---|---|---|---|---|---|---|---|---|---|---|---|---|---|---|
| UC33 | 58 | M | High | T3 | N1 | Y | N | 20 | Negative | Low | 15.22 | 53.84 | 53.33 | 56.6 | 65.17 |
| UC34 | 72 | F | High | T2 | N0 | N | N | 13 | Negative | Low | 81.18 | 95.62 | 77.82 | 85.17 | 74.58 |
| UC35 | 59 | M | High | Ta | N0 | N | N | 12 | N/A | High | 2.72 | 79.4 | 82.63 | 83.65 | 75.16 |
| UC36 | 64 | F | High | T2 | N0 | N | N | 16 | Negative | Low | 94.14 | 96.47 | 78.73 | 88.66 | 100 |
| UC37 | 56 | F | High | T3 | N1 | Y | N | 9 | N/A | Low | 60.87 | 86.06 | 71.11 | 78.6 | 100 |
| UC38 | 72 | M | High | T2 | N0 | N | N | 15 | N/A | High | 33.54 | 71.16 | 80.63 | 78.87 | 90.31 |
| UC39 | 77 | M | High | Ta | N0 | N | N | 1 | Negative | High | 38.3 | 50.83 | 70.42 | 67.19 | 67.25 |
| UC40 | 67 | F | High | T3 | N0 | Y | N | 13 | Negative | High | 36.72 | 77.5 | 64.39 | 59.99 | 91.98 |
| UC41 | 65 | M | High | T3 | N0 | Y | N | 3 | Negative | Low | 48.08 | 73.71 | 74.81 | 80.72 | 99.8 |
| UC42 | 50 | F | High | Ta | N0 | N | N | 1 | Negative | Low | 55.5 | 89.84 | 83.17 | 86.98 | 100 |
| UC43 | 77 | F | High | Ta | N0 | N | N | 3 | Negative | Low | 47.1 | 61.16 | 70.12 | 72.01 | 99.14 |
| UC44 | 69 | M | High | T1 | N0 | N | N | 6 | Negative | Low | 40.21 | 77.95 | 57.59 | 68.65 | 97.58 |
| UC45 | 65 | M | High | T3 | N0 | Y | N | 12 | Negative | Low | 52.3 | 72.56 | 75.54 | 62.52 | 94.03 |
| UC46 | 74 | M | High | T3 | N0 | Y | N | 5 | Negative | High | 37.66 | 68.04 | 71.92 | 69.13 | 88.53 |
| UC47 | 34 | F | High | T1 | N0 | N | N | 3 | Negative | Low | 49.08 | 88.92 | 83.99 | 85.64 | 100 |
| UC48 | 65 | F | High | Ta | N0 | N | N | 7 | Negative | Low | 50.18 | 80.09 | 75.89 | 72.77 | 95.57 |
| UC49 | 78 | M | High | T4 | N1 | Y | Y | 9 | Negative | High | 27.15 | 70.03 | 62.34 | 63.16 | 100 |
| UC50 | 71 | M | High | T1 | N0 | N | N | 9 | Negative | High | 32.01 | 67.27 | 53.93 | 54.99 | 100 |
| UC51 | 74 | M | High | T2 | N0 | Y | N | 2 | Negative | Low | 41.59 | 67.91 | 67 | 67.4 | 95.6 |
| UC52 | 73 | F | High | T3 | N0 | N | N | 2 | Negative | Low | 38.96 | 58.48 | 83.98 | 76.38 | 99.58 |
| UC53 | 76 | M | High | T3 | N0 | N | N | 7 | Negative | Low | 39.18 | 91.05 | 47.52 | 50.26 | 100 |
| UC54 | 72 | M | High | T3 | N0 | N | N | 18 | Negative | Low | 42.74 | 65.61 | 77.68 | 86.39 | 100 |
| UC55 | 78 | M | High | T3 | N0 | Y | N | 6 | Negative | Low | 51.75 | 67.92 | 39.77 | 58.98 | 60.51 |
| UC56 | 75 | M | Low | Ta | N0 | N | N | 11 | Negative | Low | 52.93 | 74.56 | 85.51 | 88.18 | 100 |
| UC57 | 67 | M | High | Ta | N0 | N | N | 8 | Negative | High | 28.73 | 66.93 | 87.31 | 84.89 | 95.47 |
| UC58 | 68 | F | High | T3 | N0 | N | N | 25 | Negative | High | 31.61 | 72.12 | 73.76 | 66.31 | 84.21 |
| UC59 | 76 | M | High | T3 | N0 | N | N | 49 | Negative | Low | 54.48 | 45.18 | 81.12 | 84.1 | 96.8 |
| UC60 | 55 | M | High | T1 | N0 | N | N | | N/A | High | 25.76 | 69.46 | 76.17 | 82.54 | 85.06 |
| UC61 | 80 | M | High | T2 | N0 | Y | N | 8 | Negative | High | 34.51 | 63.67 | 51.08 | 71.11 | 91.73 |
| UC62 | 63 | M | High | T3 | N0 | Y | N | 20 | Negative | Low | 58.99 | 91.11 | 60.28 | 76.15 | 100 |
| UC63 | 67 | F | High | T1 | Nx | Y | N | 12 | Negative | Low | 46.03 | 77.99 | 71.54 | 69.46 | 100 |
| UC64 | 71 | M | High | T1 | Nx | N | N | 12 | Negative | Low | 44.29 | 65.03 | 46.4 | 57.4 | 66.3 |
| UC65 | 66 | M | Low | Ta | Nx | N | N | 3 | Negative | Low | 64.8 | 83.29 | 54.8 | 64.91 | 95.63 |
| UC66 | 56 | M | High | Ta | Nx | N | N | 14 | Negative | High | 36.53 | 63.55 | 59.69 | 50.43 | 82.15 |
| UC67 | 55 | F | High | Ta | Nx | N | N | 16 | Negative | High | 36.22 | 46.58 | 75.46 | 62.58 | 85.54 |

[1]Vascular Invasion: VI; [2]Perineurial Invasion: NI; [3]Mitotic rate per 10 high power fields: MF/10

**Table S6. SLC14A1 protein levels are significantly correlated with several proteins involving in mitochondrial morphology, glycolysis and cell proliferation in UTUCs (*n* = 170)**

| | | SLC14A1 level | | *P* value |
|---|---|---|---|---|
| | | High | Low | |
| SLC2A1[&] | Low | 106 | 64 | < 0.001* |
| | High | 64 | 106 | |
| HK2[&] | Low | 101 | 69 | 0.001* |
| | High | 69 | 101 | |
| PKM[&] | Low | 105 | 65 | < 0.001* |
| | High | 65 | 105 | |
| LDHA/C[&] | Low | 99 | 71 | 0.002* |
| | High | 71 | 99 | |
| PDHA1[&] | Low | 75 | 95 | 0.030* |
| | High | 95 | 75 | |
| MFN2[&] | Low | 69 | 101 | 0.001* |
| | High | 101 | 69 | |
| pAKT1(S473)[&] | Low | 102 | 68 | < 0.001* |
| | High | 68 | 102 | |
| pMTOR(S2448)[&] | Low | 109 | 61 | < 0.001* |
| | High | 61 | 109 | |
| pRPS6(S235)[&] | Low | 103 | 67 | < 0.001* |
| | High | 67 | 103 | |
| pEIF4EP1(S65)[&] | Low | 100 | 70 | 0.0001 |
| | High | 70 | 100 | |
| Ki-67[#] | Low | 105 | 70 | < 0.001* |
| | High | 65 | 100 | |

[&], High expression level defined as expression no less than median H-score

[#], High expression level defined as expression no less than 20% tumor cell nuclei

**Table S7. SLC14A1 protein levels are significantly correlated with several proteins involving in glycolysis, mitochondrial morphology and cell proliferation in UBUCs (*n* = 148)**

| | | SLC14A1 level | | |
|---|---|---|---|---|
| | | High | Low | *P* value |
| SLC2A1[&] | Low | 95 | 52 | < 0.001* |
| | High | 53 | 95 | |
| HK2[&] | Low | 91 | 56 | < 0.001* |
| | High | 57 | 91 | |
| PKM[&] | Low | 99 | 48 | < 0.001* |
| | High | 49 | 99 | |
| LDHA/C[&] | Low | 92 | 55 | < 0.001* |
| | High | 56 | 92 | |
| PDHA1[&] | Low | 59 | 88 | 0.001* |
| | High | 89 | 59 | |
| MFN2[&] | Low | 39 | 108 | < 0.001* |
| | High | 109 | 39 | |
| pAKT1(S473)[&] | Low | 84 | 63 | 0.017* |
| | High | 64 | 84 | |
| pMTOR(S2448)[&] | Low | 89 | 58 | < 0.001* |
| | High | 59 | 89 | |
| pRPS6(S235)[&] | Low | 83 | 64 | 0.031* |
| | High | 65 | 83 | |
| pEIF4EP1(S65)[&] | Low | 88 | 59 | 0.001* |
| | High | 60 | 88 | |
| Ki-67[#] | Low | 84 | 54 | < 0.001* |
| | High | 64 | 93 | |

[&], High expression level defined as expression no less than median H-score

[#], High expression level defined as expression no less than 20% tumor cell nuclei

## Supplementary figures

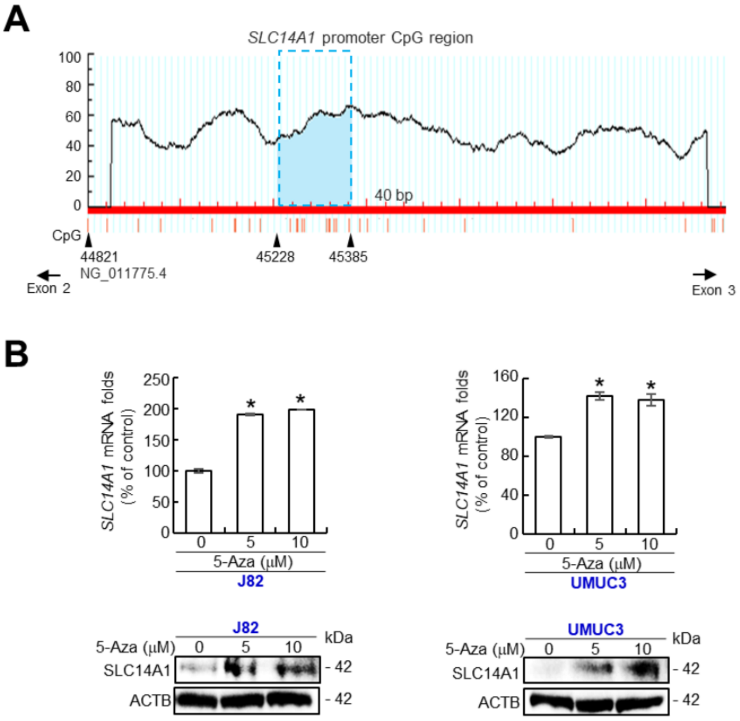


**Figure S1.** Hypermethylation in the proximal promoter region of the *SLC14A* gene downregulates its mRNA and protein levels in urothelial carcinoma-derived cells. **(A)** MethPrimer predicted a CpG island located between exon 2 and exon 3 in the *SLC14A1* gene. **(B)** Quantitative RT-PCR and immunoblot analysis showed that treatment with a DNA methylation inhibitor 5-aza-2'-deoxycytidine (5-Aza, 5 and 10 μM) increased *SLC14A1* mRNA and their corresponding protein levels in J82 and UMUC3 cells. All experiments were performed in triplicate and results are expressed as the mean ± SD. For immunoblot analysis, one representative image is shown and actin, beta (ACTB) served as a loading control. Statistical significance: *$P < 0.05$.

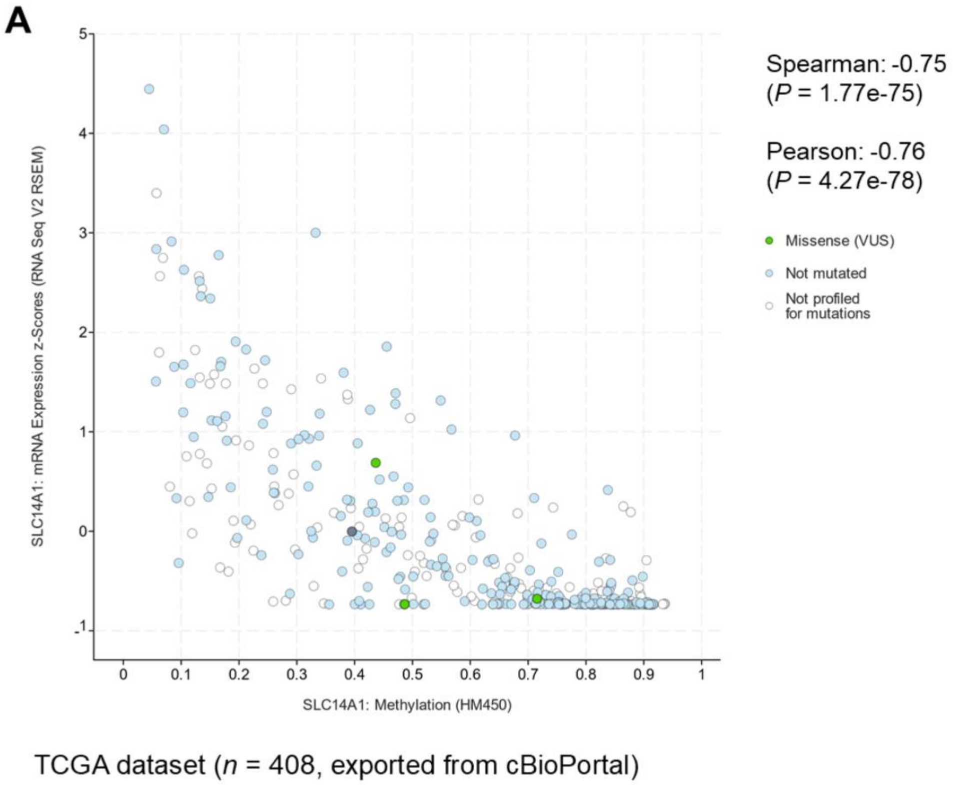


**Figure S2.** *SLC14A1* transcript level, methylation, and mutation statuses were evaluated in 408 UBUC samples from the TCGA database by using the cBioportal platform. The *SLC14A1* mRNA level is significantly negatively correlated with its methylation status. The aforementioned evidence suggests hypermethylation in the *SLC14A1* promoter region may be a crucial mechanism responsible for *SLC14A1* silencing in UC patients.

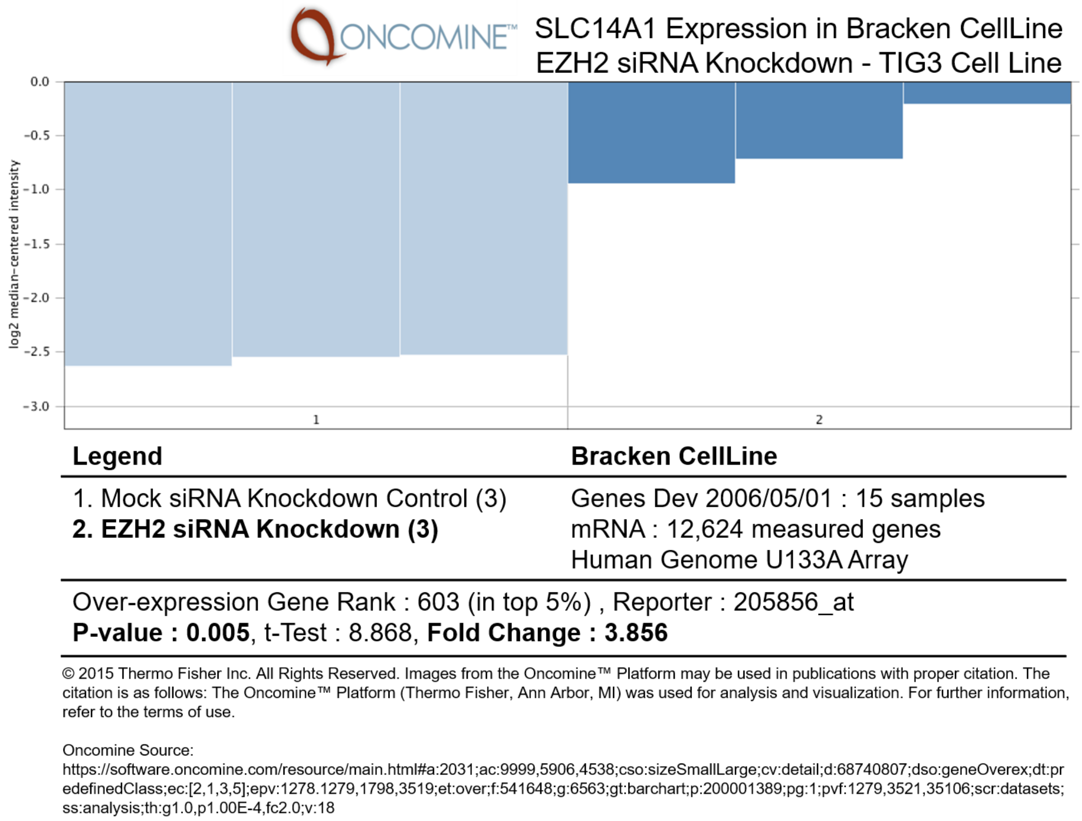


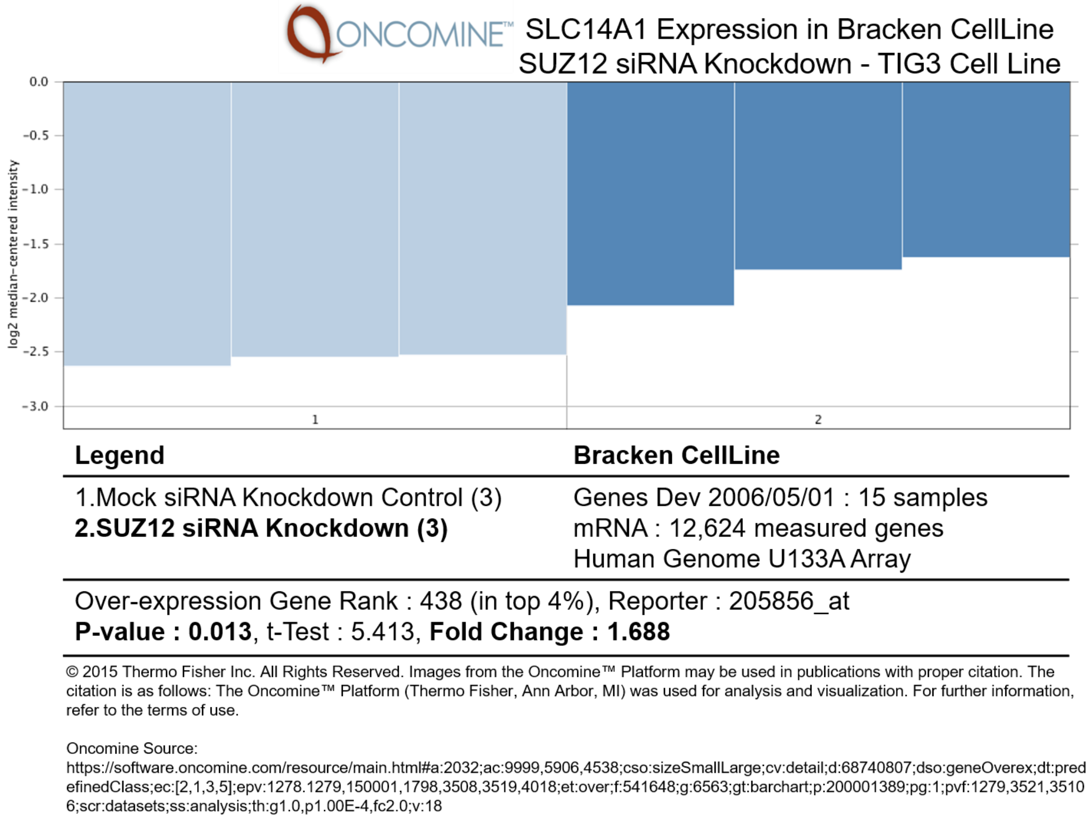


**Figure S3.** Reappraisal on the public genome-wide database shows negative correlations between *SLC14A1* and *EZH2*; *SLC14A1* and *SUZ12* mRNA levels in the TIG-3 cell line.

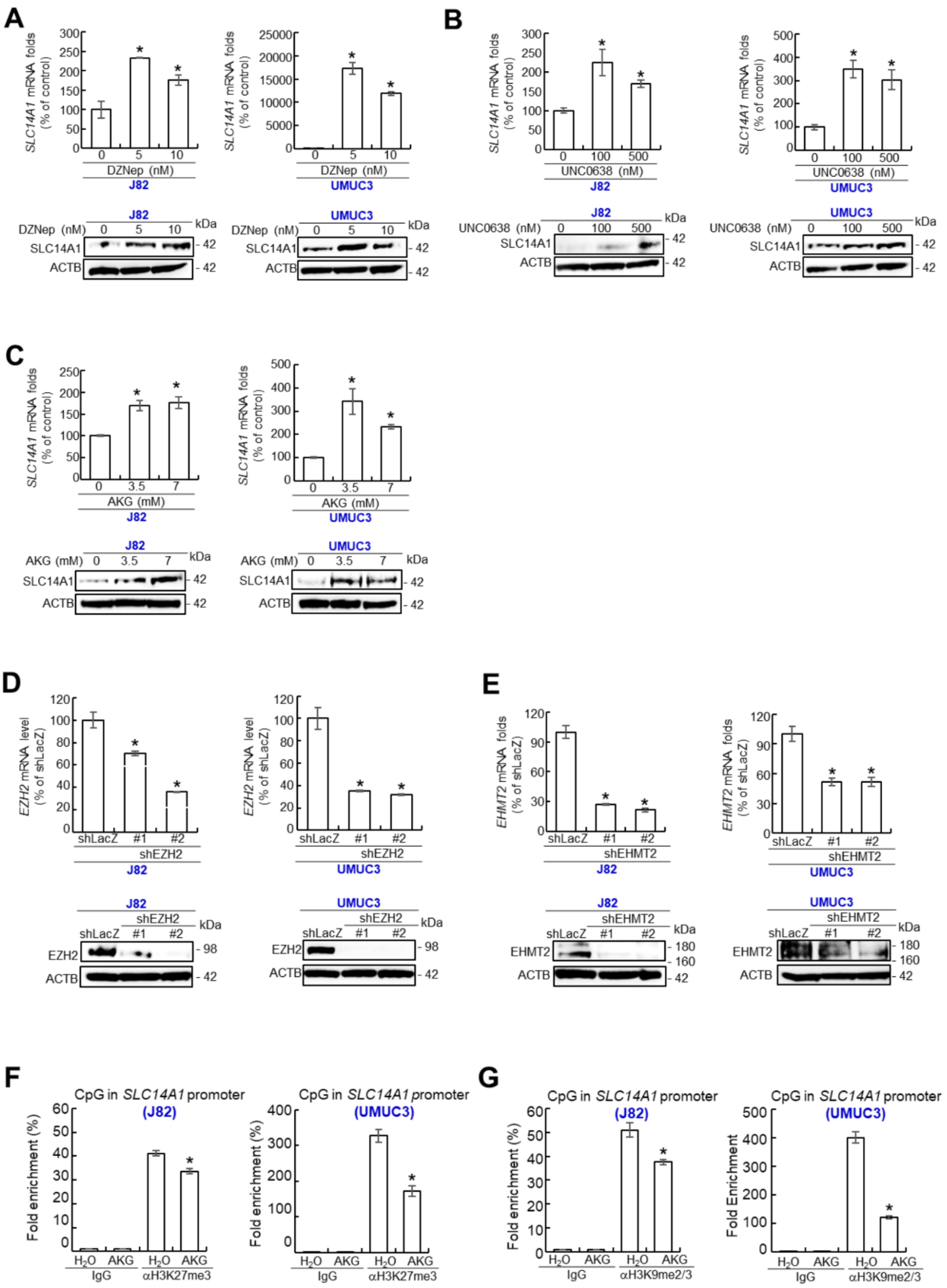


**Figure S4.** Histone methylation in the *SLC14A1* promoter region downregulated its mRNA and protein levels in urothelial carcinoma-derived cells. Quantitative RT-PCR and immunoblot analysis showed that treatment with an EZH2 inhibitor, DZNep (5 or 10 nM; 72 h), an EHMT2 inhibitor, UNC0638 (100 or 500 nM; 72 h) or a histone lysine demethylation reagent, α-ketoglutarate (AKG: 3.5 or 7 mM; 72 h) upregulated endogenous *SLC14A1* mRNA and their corresponding protein levels in J82 and UMUC3 cells **(A, B, C)**. Stable knockdown of the *EZH2* and *EHMT2* gene with 2 distinct small hairpin RNA (shRNA) interference clones (shEZH2#1 or shEZH2#2; shEHMT2#1 or shEHMT2#2), respectively, downregulated *EZH2* and *EHMT2* mRNA and their corresponding protein levels in J82 and UMUC3 cells **(D, E)**. Quantitative chromatin immunoprecipitation (qChIP) assay by probing anti-H3K27me3 (αH3K27me3) and -H3K9me2/3 antibody

(αH3K9me2/3), respectively, and PCR using primers to amplify the CpG region (see also Supplementary Table 5) indicated that AKG treatment (3.5 mM) for 72 h decreased histone methylation level in the *SLC14A1* promoter region compared to the control ($H_2O$) in both cell lines **(F, G)**. All experiments were performed in triplicate and results are expressed as the mean ± SD. For immunoblot analysis, one representative image is show and actin, beta (ACTB) served as a loading control. Statistical significance: $*P < 0.05$.

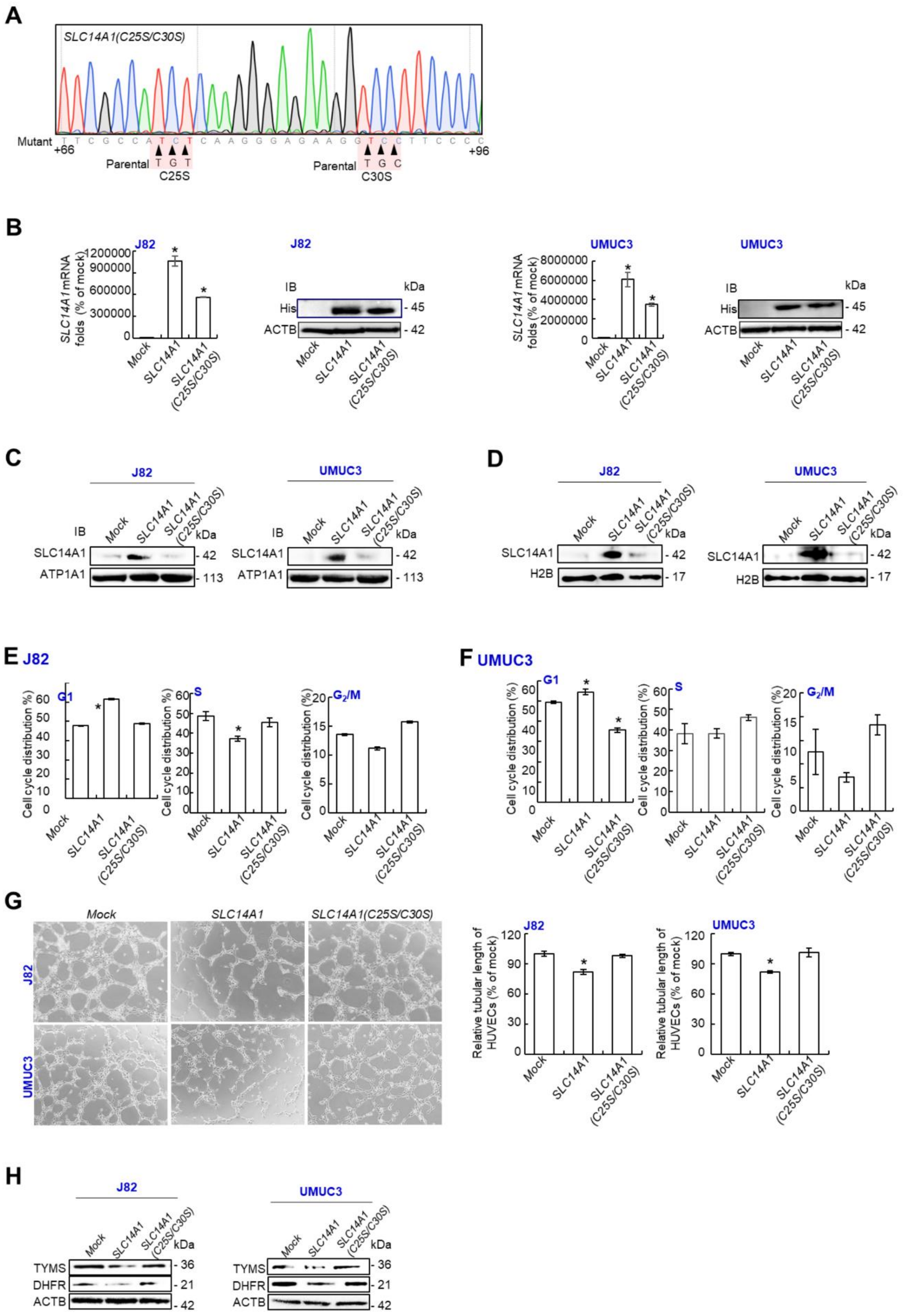


**Figure S5.** Membranous SLC14A1 protein is essential for its tumor suppressive roles. **(A)** Using the wild-type pLVX-puro-6HIS-*SLC14A1*_v1 plasmid as the template, site-directed mutagenesis was performed to generate a double mutation pLVX-puro-6HIS-*SLC14A1(C25S/C30S)*_v1 plasmid, which is not able to express in the cytoplasmic membrane. Mutations were verified by Sanger

sequencing. **(B)** Quantitative RT-PCR and immunoblot analysis by probing anti-His antibody confirmed that stable overexpression of the *SLC14A1* or *SLC14A1(C25S/C30S)* gene upregulated *SLC14A1*, *SLC14A1(C25S/C30S)* mRNA, His-SLC14A1 and His-SLC14A1(C25S/C30S) fusion protein levels in J82 and UMUC3 cells compared to the *mock* ($P < 0.05$). **(C, D)** Membrane, nuclear/cytosol fractionation along with immunoblot analysis showed that stable *SLC14A1* overexpression upregulated membranous and nuclear SLC14A1 protein levels while stable *SLC14A1(C25S/C30S)* overexpression was not able to upregulate either membranous or nuclear SLC14A1 protein level in J82 and UMUC3 cells. ATP1A1 and H2B served as membranous and nuclear control, respectively. **(E, F)** Flow cytometric analysis indicated that *SLC14A1(C25S/C30S)* overexpression failed to induce $G_1$ cell cycle arrest compared to *SCL14A1* overexpression. **(G)** The conditioned media from *SLC14A1(C25S/C30S)*-overexpressed J82 or UMUC3 cells were not able to suppress HUVEC tube formation compared to conditioned media from *SLC14A1*-overexpressed cells. **(H)** Immunoblot analysis showed that overexpression of the *SLC14A1* gene notably downregulated while stable overexpression of the *SLC14A1(C25S/C30S)* gene reverted TYMS and DHFR protein levels compared to the *mock* in J82 and UMUC3 cells. All experiments were performed in triplicate and results are expressed as the mean ± SD. For immunblot and HUVEC tube formation, representative images are shown. For immunoblot analysis, ATP1A1 and H2B served as membranous and nuclear loading control, respectively. *$P < 0.05$.

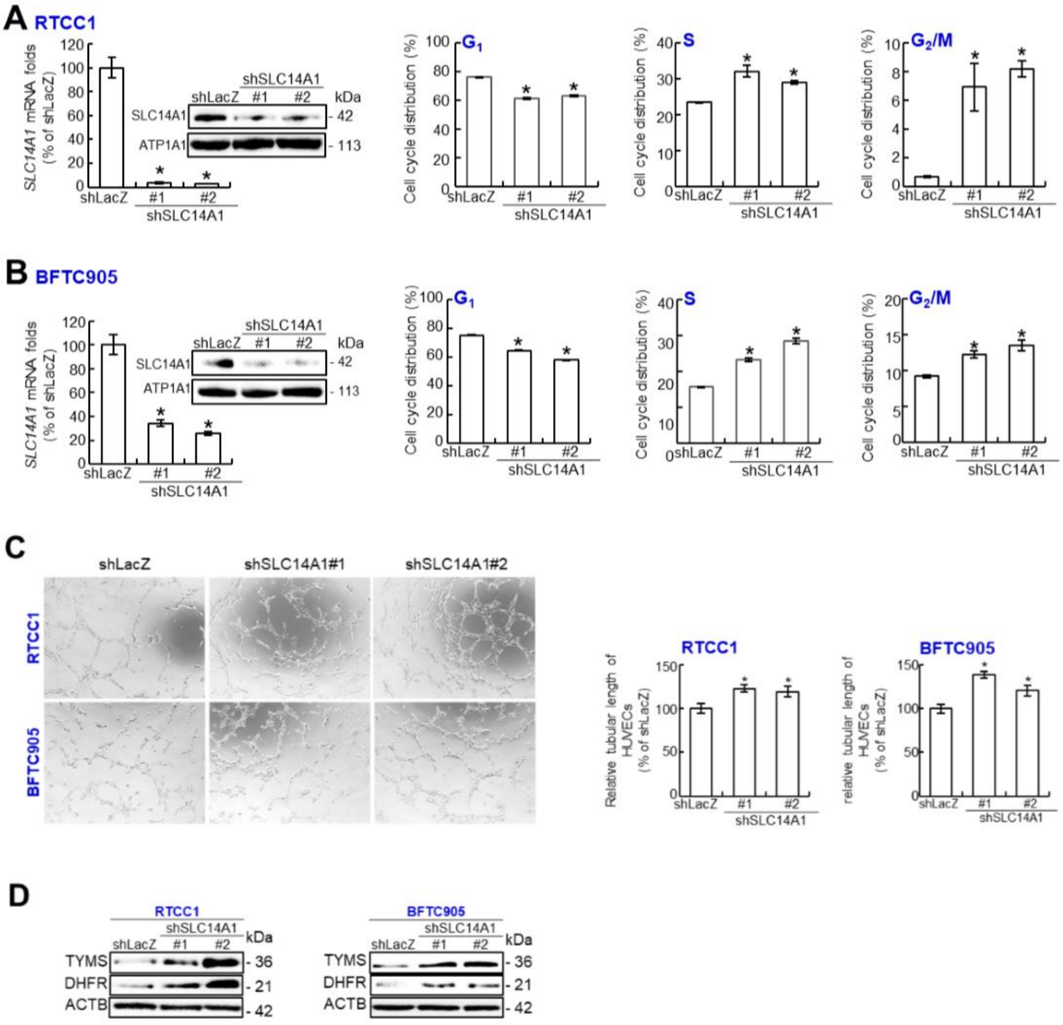


**Figure S6.** Stable knockdown of the *SLC14A1* gene promotes cell cycle progression and HUVEC tube formation in UC-derived cells. Quantitative RT-PCR, membrane protein extraction, immunoblot and flow cytometric assays identified that stable knockdown of the *SLC14A1* gene with 2 distinct shRNA clones downregulated *SLC14A1* mRNA and their corresponding membranous SLC14A1 protein levels, however, promoted cell cycle $G_1$-S transition in RTCC1 and BFTC905 cells **(A, B)**. **(C)** HUVECs (7,000 cells in 25 μL medium) were treated with conditioned medium (25 μL) from *SLC14A1*-knockdown RTCC1 and BFTC905 cells, respectively, and total branching lengths of tube formation were next examined using the ImageJ software. Relative tubular lengths of HUVECs (%) were increased after treatment with conditioned media for 6 h. **(D)** Stable knockdown of the *SLC14A1* gene with 2 distinct shRNA clones notably upregulated TYMS and DHFR protein levels. All experiments were performed in triplicate and results are expressed as the mean ± SD. For immunoblot and tube formation assay, representative images are shown. ATPase Na+/K+ transporting subunit beta 1 (ATP1A1) and actin, beta (ACTB) served as membrane and cytosol control, respectively, for immunoblot analysis. Statistical significance: *$P < 0.05$.

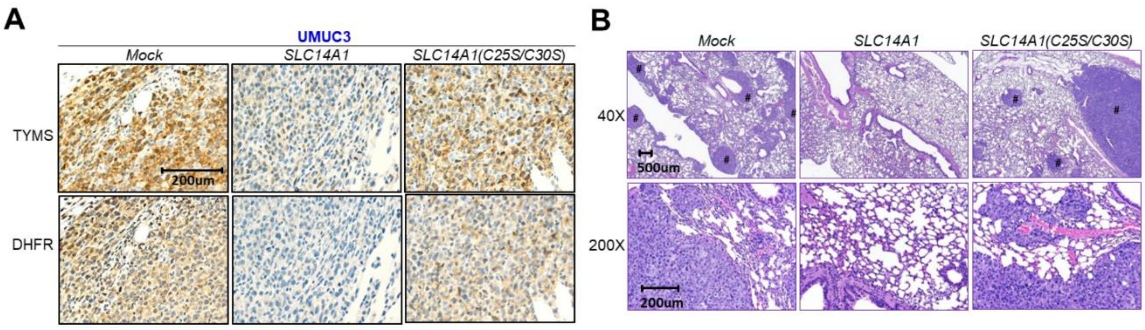


**Figure S7.** Dysfunctional SLC14A1 abolishes its tumor suppressive roles in vivo. **(A)** Immunohistochemistry identified that TYMS and DHFR were remarkably downregulated in xenografts from *SLC14A1*- compared to those from *SLC14A1(C25S/C30S)*-overexpressed UMUC3 cells. **(B)** Hematoxylin and eosin (H&E) staining showed that highly lung-metastasis was observed in *mock* (UMUC3 cells only) and *SLC14A1(C25S/C30S)*- compared to *SLC14A1*-overexpressed UMUC3 cells by tail vein injection, where # indicated the metastatic tumor cells in the lung.

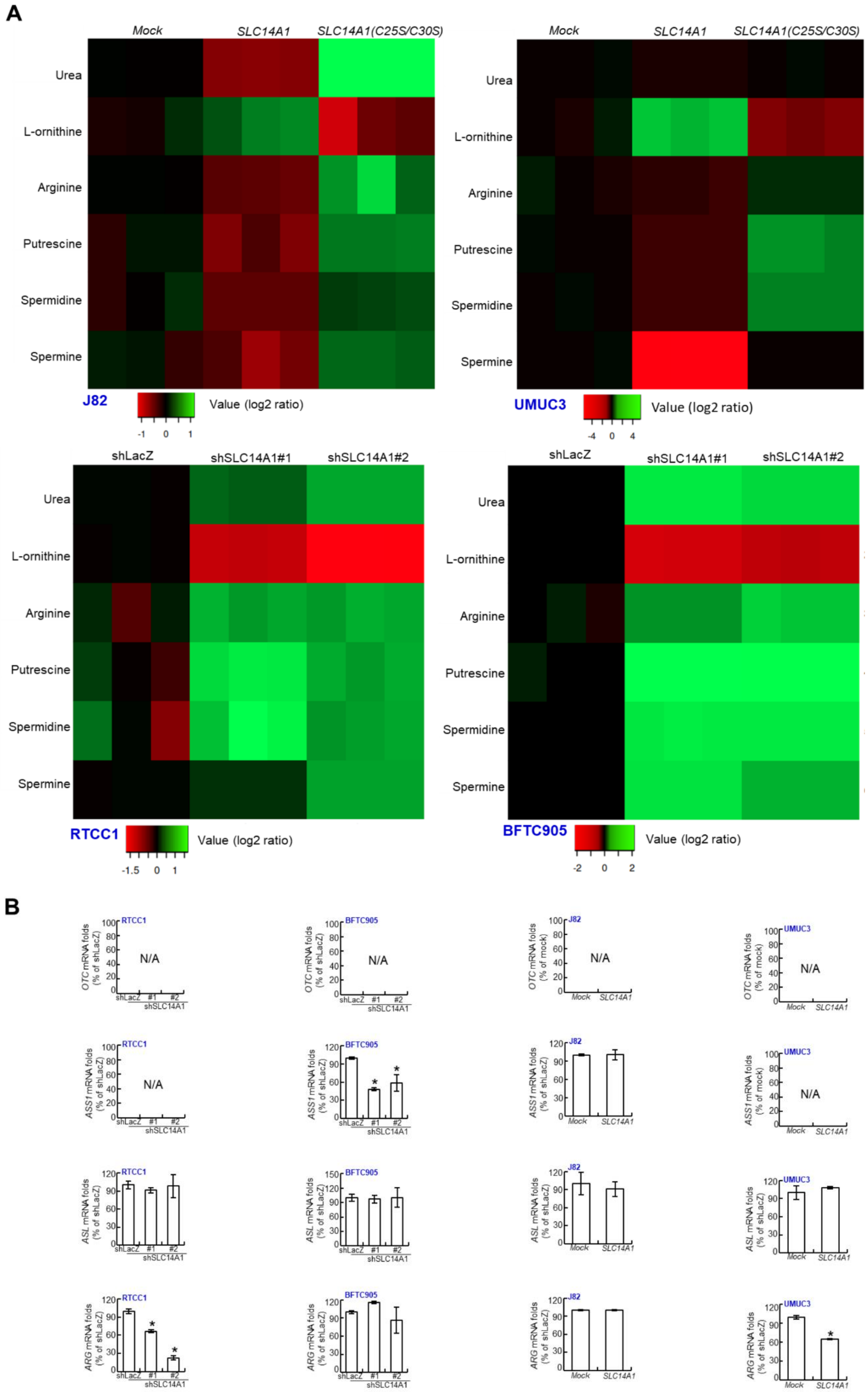
A
Mock
SLC14A1
SLC14A1(C25S/C30S)
Urea
L-ornithine
Arginine
Putrescine
Spermidine
Spermine
J82
Value (log2 ratio)
-1 0 1
UMUC3
Value (log2 ratio)
-4 0 4
shLacZ
shSLC14A1#1
shSLC14A1#2
RTCC1
Value (log2 ratio)
-1.5 0 1
BFTC905
Value (log2 ratio)
-2 0 2
B
OTC mRNA folds (% of shLacZ)
OTC mRNA folds (% of mock)
ASS1 mRNA folds (% of shLacZ)
ASS1 mRNA folds (% of mock)
ASL mRNA folds (% of shLacZ)
ARG mRNA folds (% of shLacZ)
N/A
shLacZ #1 #2
shSLC14A1
Mock SLC14A1

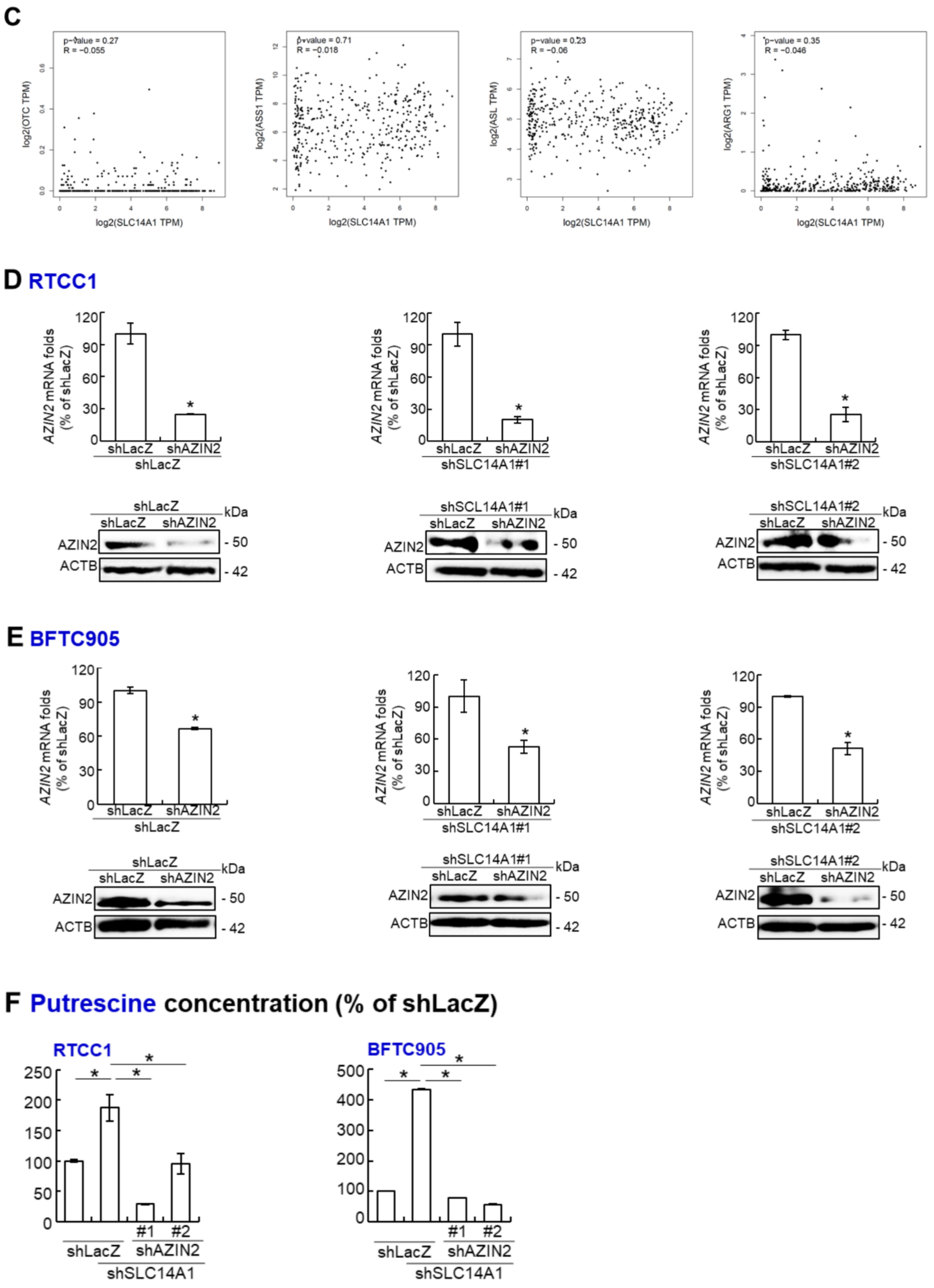


**Figure S8.** Loss of membranous SLC14A1 accumulates several oncometabolites except for L-ornithine involving in the urea cycle and promotes polyamine biosynthesis. **(A)** UPLC-MS/MRM identified that stable overexpression of the *SLC14A1* gene reduced arginine, urea, putrescine, spermidine and spermine in J82 and UMUC3 cells while increased L-ornithine concentration. Stable overexpression of the *SLC14A1(C25S/C30S)* gene (non-membranous SLC14A1, loss of urea transport ability) in J82 and UMUC3 cells, and stable knockdown of the *SLC14A1* gene in RTCC1 and BFTC905 cells enhanced urea, putrescine, spermidine and spermine accumulation in cells

besides L-ornithine (*n* =3 for each cell line). **(B)** Stable knockdown of the *SLC14A1* gene in RTCC1 and BFTC905 cells or overexpression of the *SLC14A1* gene in J82 and UMUC3 cells were not able to consistently regulate the mRNA levels of several metabolic enzymes including *OTC*, *ASS1*, *ASL* and *ARG*. **(C)** Data mining on 414 urothelial bladder cancer (BLCA) samples in the TCGA database showed that *SLC14A1* mRNA level was not correlated to those of *OTC*, *ASS1*, *ASL* and *ARG1* abundance, respectively. **(D, E)** Stable knockdown of the *AZIN2* gene (biosynthesis from arginine and then agmatine to putrescine) in RTCC1 and BFTC905 cells downregulated *AZIN2* mRNA and their corresponding protein levels. Stable knockdown the *AZIN2* gene in stable *SLC14A1*-knockdwon RTCC1 and BFTC905 cells (shSLC14A1#1 & shSLC14A1#2) also downregulated *AZIN2* mRNA and their corresponding protein levels. **(F)** Stable knockdown of the *AZIN2* gene with 2 distinct shRNA clones (shAZIN2#1 & shAZIN#2) in stable *SLC14A1*-knockdown RTCC1 and BFTC905 cells decreased *SLC14A1*-knockdown-induced putrescine concentrations. All experiments were performed in triplicate and results are expressed as the mean ± SD. For immunoblot analysis, one representative image is shown, and actin, beta (ACTB) served as a loading control. Statistical significance: $*P < 0.05$.

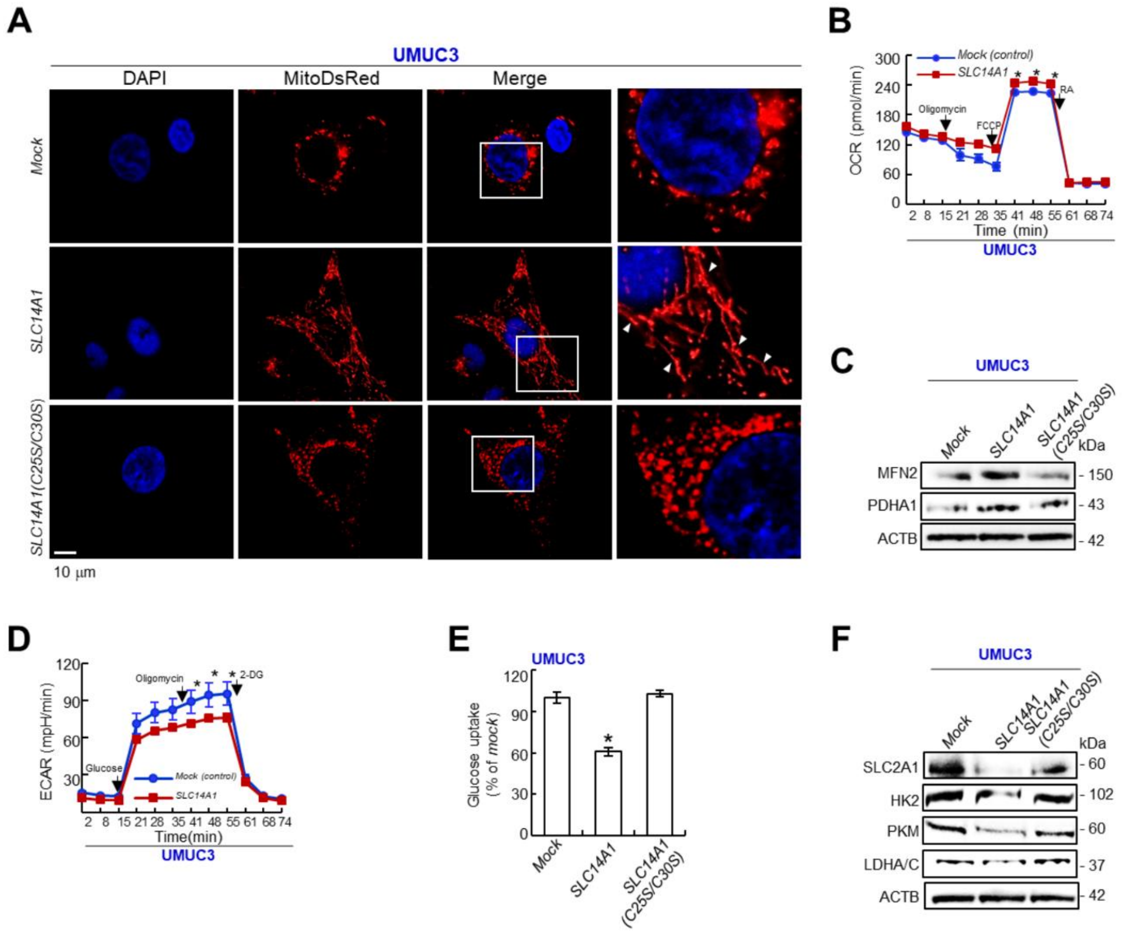
A
UMUC3
DAPI
MitoDsRed
Merge
Mock
SLC14A1
SLC14A1(C25S/C30S)
10 μm
B
Mock (control)
SLC14A1
OCR (pmol/min)
Oligomycin
FCCP
RA
Time (min)
UMUC3
C
UMUC3
Mock
SLC14A1
SLC14A1 (C25S/C30S)
kDa
MFN2
PDHA1
ACTB
- 150
- 43
- 42
D
ECAR (mpH/min)
Glucose
Oligomycin
2-DG
Mock (control)
SLC14A1
Time(min)
UMUC3
E
UMUC3
Glucose uptake (% of mock)
Mock
SLC14A1
SLC14A1 (C25S/C30S)
F
UMUC3
Mock
SLC14A1
SLC14A1 (C25S/C30S)
kDa
SLC2A1
HK2
PKM
LDHA/C
ACTB
- 60
- 102
- 60
- 37
- 42

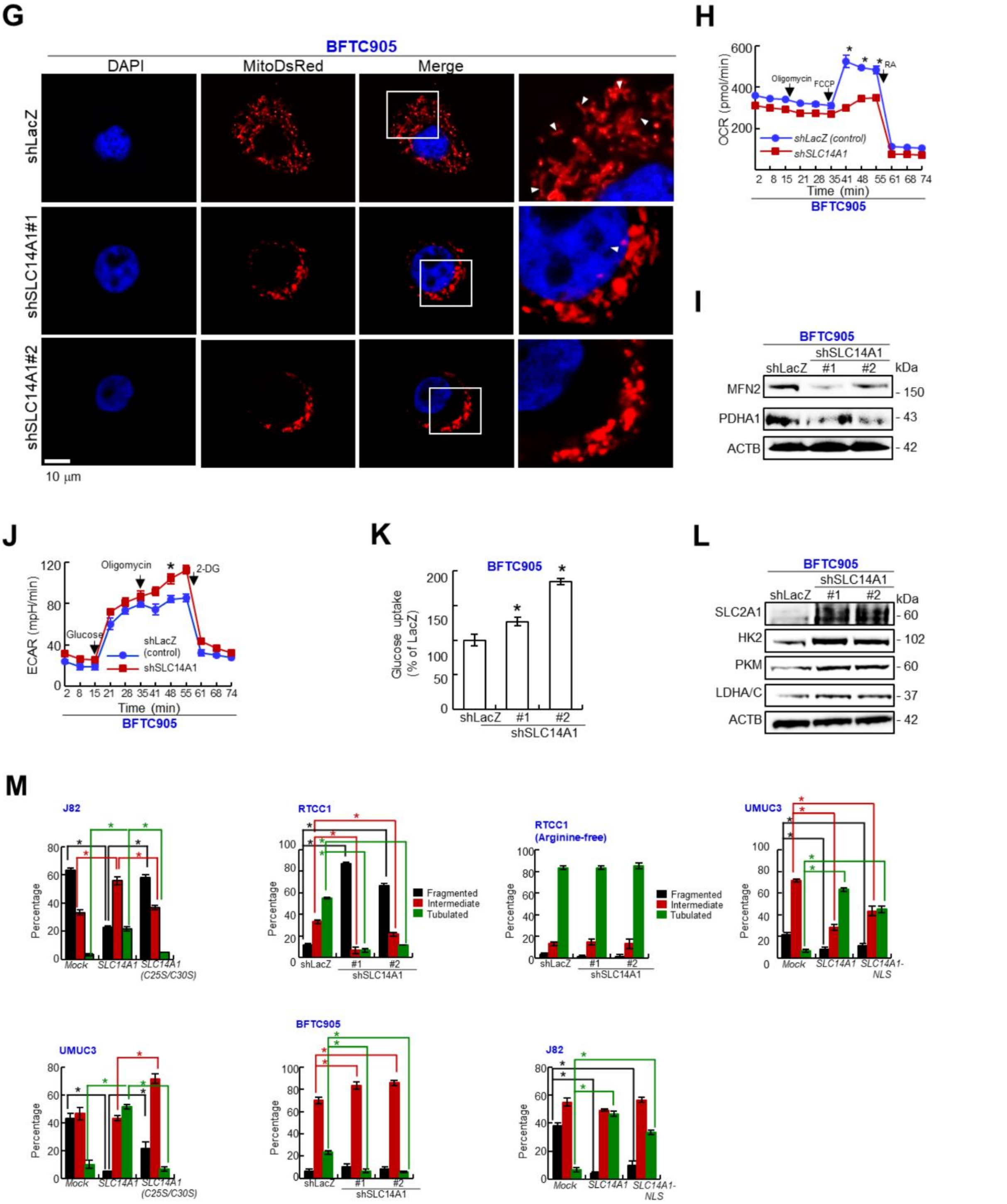


**Figure S9.** Wild-type *SLC14A1* induces while mutation *SLC14A1(C25S/C30S)* or knockdown of the *SLC14A1* gene suppresses mitochondrial fusion, oxygen consumption rate (OCR), extracellular acidification rate (ECAR) and alterations of the expression levels of several aerobic glycolysis- and mitochondrial respiration-related proteins in UC-derived cells. A plasmid containing a mitochondrial targeting sequence fused to a red fluorescence tag (pLV-MitoDsRed) was used to generate replication-incompetent lentivirus. Transduction of the lentiviral particles containing MitoDsRed showed the morphology and localization of each mitochondrion within a single cell. Immunocytofluorescence with confocal microscopy, Seahorse XFp Analyser and immunoblot assays demonstrated that *SLC14A1*-overexpression induced mitochondrial fusion **(A)**, increased OCR **(B)**, upregulated MFN2 and PDHA1 protein levels **(C)**, decreased ECAR **(D)** and glucose

uptake **(E)** and downregulated SLC2A1, HK2, PKM and LDHA/C protein levels compared to the *mock* (control) **(F)** in UMUC3 cells. On the other hand, overexpression of the mutation *SLC14A1(C25S/C30S)* gene in UMUC3 cells **(A-F)** and knockdown of the *SLC14A1* gene with 2 distinct shRNA clones in BFTC905 cells induced mitochondrial fission **(G)**, decreased OCR **(H)**, downregulated MFN2 and PDHA1 protein levels **(I)**, increased ECAR **(J)** and glucose uptake **(K)** and upregulated SLC2A1, HK2, PKM, LDHA/C protein levels compared to the control (*mock* or shLacZ) **(L)**. **(M)** Quantification of the changes in mitochondrial morphagy are shown based on our previous study (Cheng et al. 2016 *Cancer Research* 76:5006-5018). For OCR and ECAR analysis, the arrows point to oligomycin, carbonyl cyanide-4-(trifluoromethoxy)phenylhydrazone (FCCP), rotenone and antimycin A (RA), glucose and 2-deoxy-glucose (2-DG), respectively, were loaded at each indicated time point (min). All experiments were performed in triplicate and results are expressed as the mean ± SD. For immunocytofluorescence and immunoblot analysis, representative images are shown. Actin, beta (ACTB) served as a loading control for immunoblot analysis. Statistical significance: $*P < 0.05$.

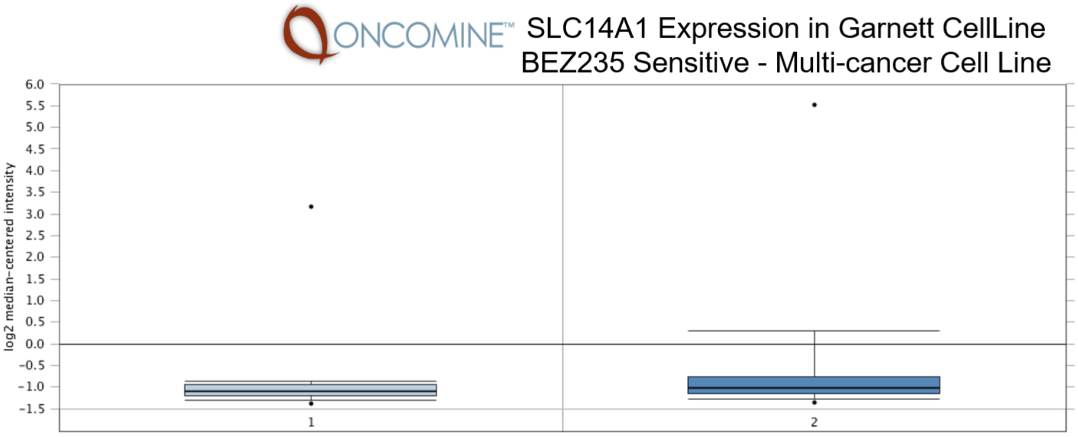


| Legend | Bracken CellLine |
|---|---|
| 1. BEZ235 Resistant (74)<br>**2. BEZ235 Sensitive (173)** | Nature 2012/03/28 : 732 samples<br>mRNA : 12,624 measured genes<br>Human Genome U133A Array |

Over-expression Gene Rank : 608 (in top 5%) , Reporter : 205856_at
**P-value : 0.002**, t-Test : 2.926, **Fold Change : 1.287**

© 2015 Thermo Fisher Inc. All Rights Reserved. Images from the Oncomine™ Platform may be used in publications with proper citation. The citation is as follows: The Oncomine™ Platform (Thermo Fisher, Ann Arbor, MI) was used for analysis and visualization. For further information, refer to the terms of use.

Oncomine Source:
https://software.oncomine.com/resource/main.html#a:8962;cso:sizeSmallLarge;cv:detail;d:156636665;dso:geneOverex;dt:predefinedClass;ec:[2,1,3];epv:1084.1242.800072537,150001.151078.150003,150845,3519,3522;et:over;f:541648;g:6563;gt:boxplot;p:200011996;pg:1;pvf:3521,35106,800072537,800072573;scr:datasets;ss:analysis;th:g100.0,p1.00E-4,fc2.0;v:18

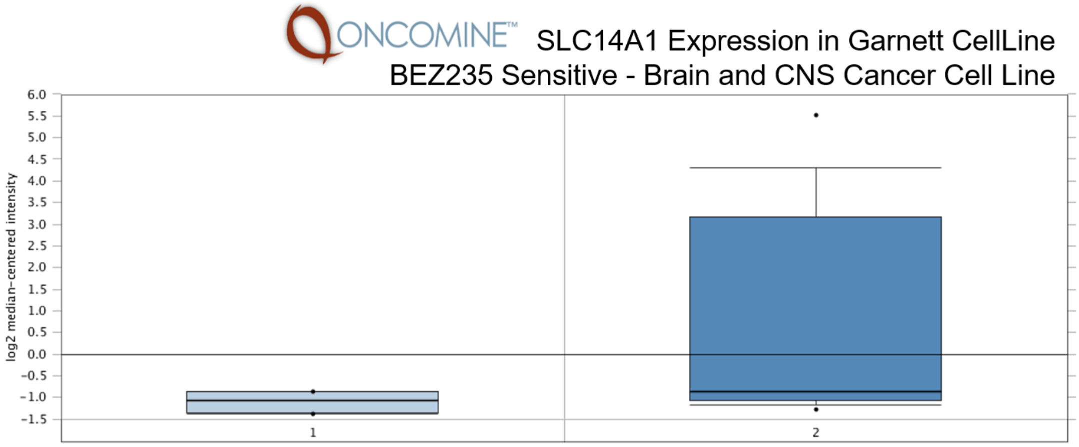


| Legend | Bracken CellLine |
|---|---|
| 1. BEZ235 Resistant (7)<br>**2. BEZ235 Sensitive (19)** | Nature 2012/03/28 : 732 samples<br>mRNA : 12,624 measured genes<br>Human Genome U133A Array |

Over-expression Gene Rank : 568 (in top 5%) , Reporter : 205856_at
**P-value : 9.73E-4**, t-Test : 3.602, **Fold Change : 3.996**

© 2015 Thermo Fisher Inc. All Rights Reserved. Images from the Oncomine™ Platform may be used in publications with proper citation. The citation is as follows: The Oncomine™ Platform (Thermo Fisher, Ann Arbor, MI) was used for analysis and visualization. For further information, refer to the terms of use.

Oncomine Source:
https://software.oncomine.com/resource/main.html#a:8617;cso:sizeSmallLarge;cv:detail;d:156636665;dso:geneOverex;dt:predefinedClass;ec:[2,1,3];epv:1084.1242.800072537,150001.151078.150003,150845,3519,3522;et:over;f:541648;g:6563;gt:boxplot;p:200012075;pg:1;pvf:3521,35106,800072537,800072573;scr:datasets;ss:analysis;th:g100.0,p1.00E-4,fc2.0;v:18

**Figure S10.** Reappraisal on a public genome-wide database identifies that among a series of cell lines, high *SLC14A1* (*n* = 19) were sensitive while low *SLC14A1* (*n* = 7) mRNA levels were resistant to a PI3K/MTOR dual inhibitor (BEZ235) treatment.

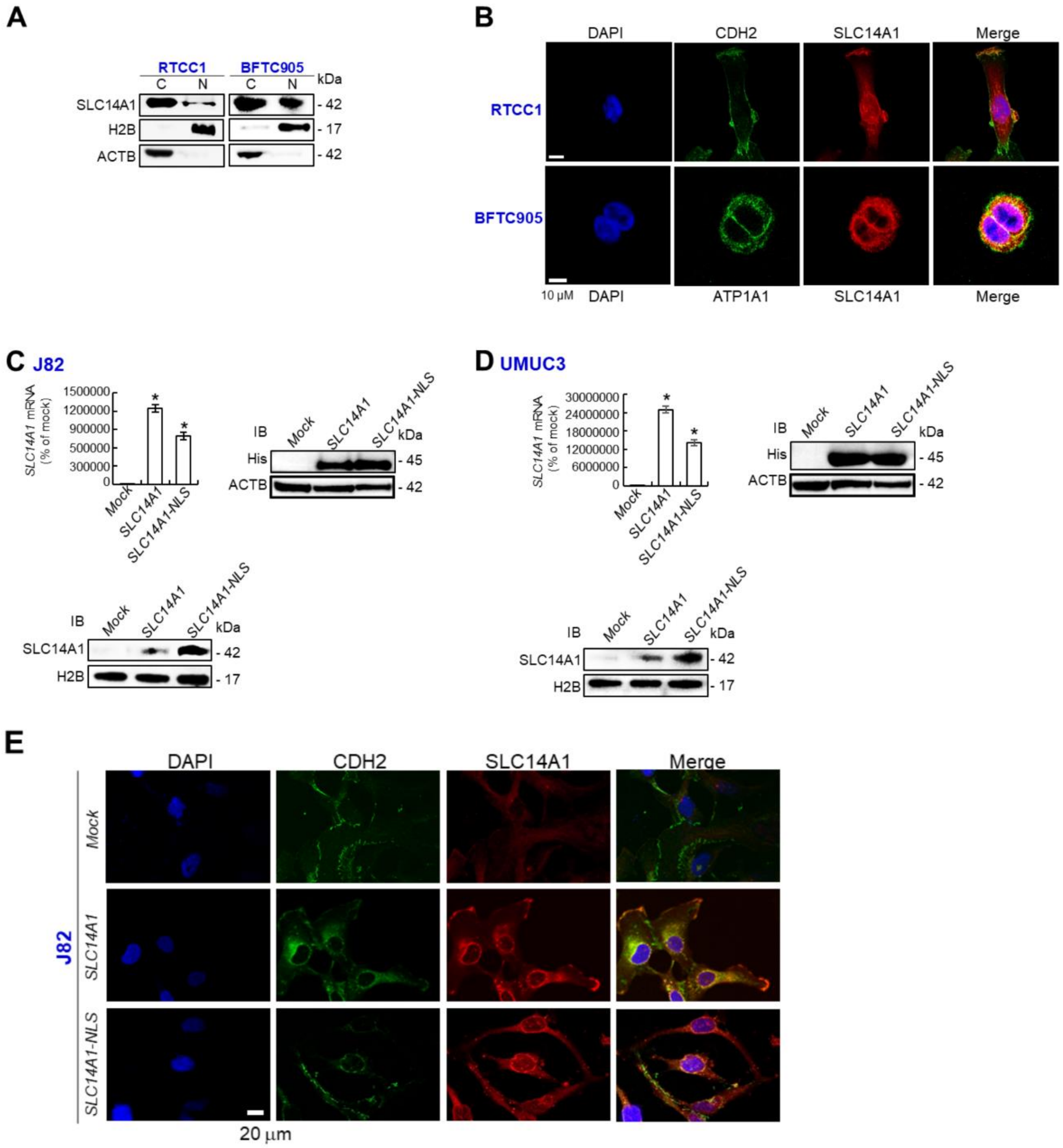


**Figure S11.** A construct containing a nuclear localization signal fused to the *SLC14A1* gene notably increases nuclear SLC14A1 protein levels in UC-derived cells. **(A)** In RTCC1 and BFTC905 cells, cytosol (C)/nuclear (N) fractionation and immunoblot analyses showed that SLC14A1 protein was expressed in cytosol and nucleus. However, cytosolic SLC14A1 protein level is much higher than the nuclear form. **(B)** A construct containing the nuclear localization signal [NLS: CCCAAGAAGAAGAGAAAGGTG (PKKKRKV)] fused to the C-terminal of the *SLC14A1* gene, pLVX-Puro-6HIS-SLC14A1-NLS_v1, was used to generate replication-incompetent lentivirus for stable overexpression of the *SLC14A1-NLS* gene. Immunocytofluorescence with confocal microscopic assay identified that SLC14A1 protein is expressed in the plasma membrane, cytosol and nucleus. **(C, D)** Quantitative RT-PCR and immunoblot analysis by probing anti-His antibody showed that stable overexpression of the *SLC14A1* and *SLC14A1-NLS* genes notbly upregulated *SLC14A1*, *SLC14A1-NLS* mRNA, His-SLC14A1 and His-SLC14A1-NLS fusion proteins, compared to the *mock* in J82 and UMUC3 cells. Cytosol (C)/nuclear (N) fractionation and immunoblot analyses further demonstrated that SLC14A1-NLS was higher expressed in the nuclear compartment. **(E)** Immunocytofluorescence with confocal microscopic assays further verified that higher SLC14A1 protein level was identified in the nucleus after overexpression of the *SLC14A1-*

*NLS* gene compared to overexpression of the *SLC14A1* gene in J82 cells. Subcellular localization specific markers used were DAPI: nuclear; CDH2 and ATP1A1: membrane; H2B: nuclear; ACTB: cytosol. All experiments were performed in triplicate and results are expressed as the mean ± SD. For immunoblot and immunocytofluorescence assays, representative images are shown. Statistical significance: **P* < 0.05.

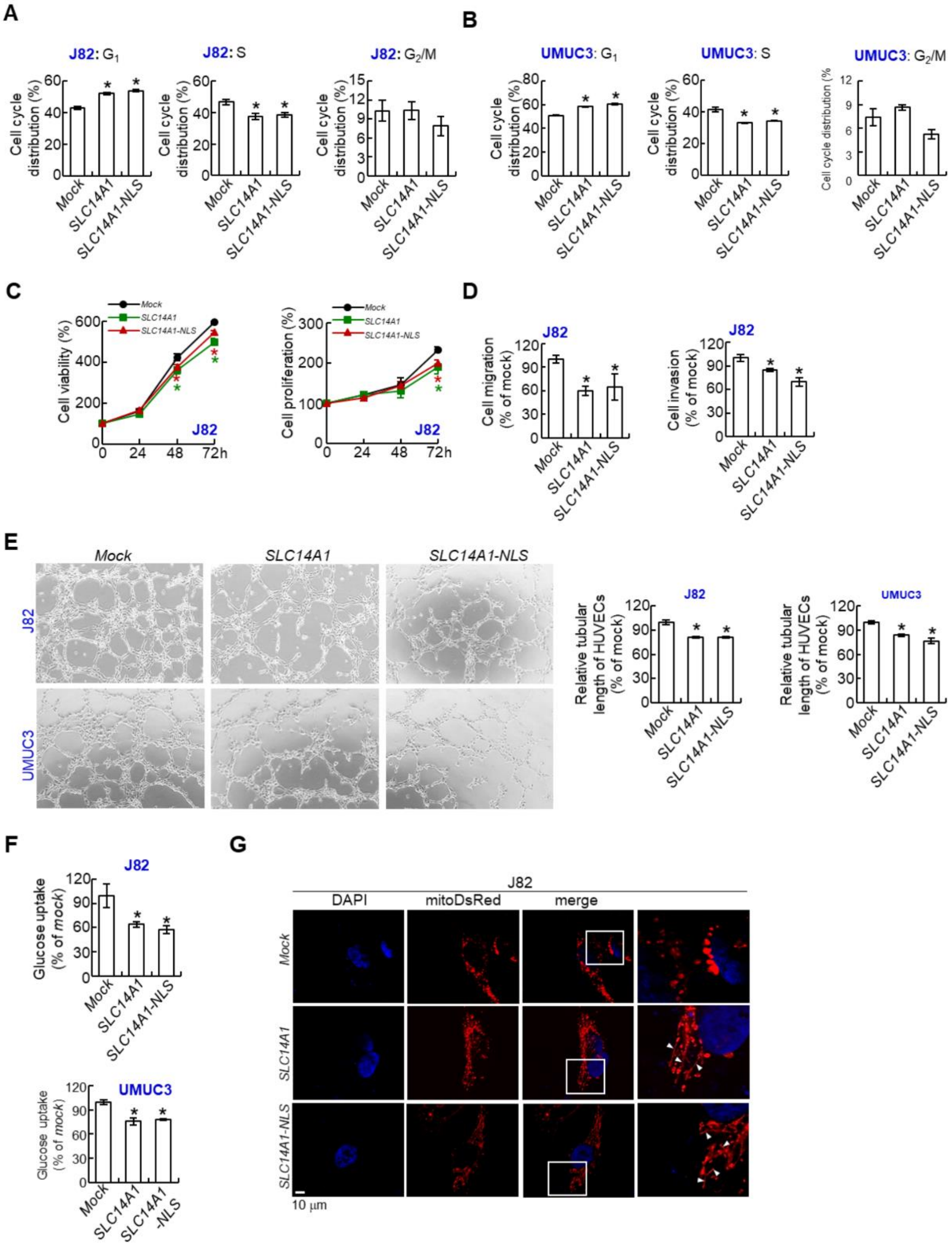
A
J82: G1
J82: S
J82: G2/M
Cell cycle distribution (%)
Mock
SLC14A1
SLC14A1-NLS
B
UMUC3: G1
UMUC3: S
UMUC3: G2/M
Cell cycle distribution (%)
C
Mock
SLC14A1
SLC14A1-NLS
Cell viability (%)
Cell proliferation (%)
J82
0 24 48 72h
D
J82
Cell migration (% of mock)
Cell invasion (% of mock)
E
Mock
SLC14A1
SLC14A1-NLS
J82
UMUC3
Relative tubular length of HUVECs (% of mock)
F
J82
UMUC3
Glucose uptake (% of mock)
G
J82
DAPI
mitoDsRed
merge
10 μm

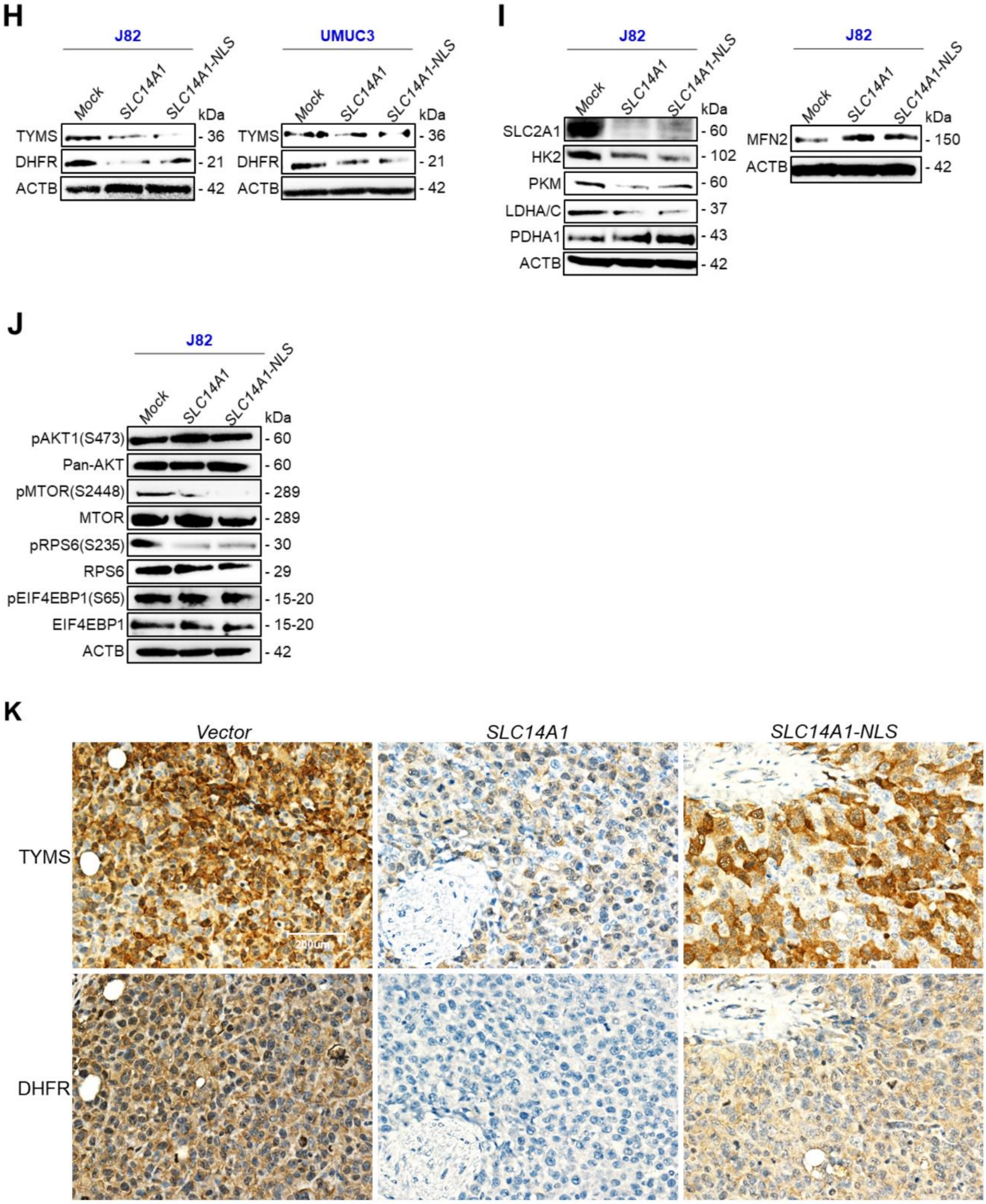


**Figure S12.** Both wild-type SLC14A1 and nuclear SLC14A1 proteins exhibit tumor suppressive roles in vitro and in vivo. **(A-D)** Flow cytometric, XTT, cell proliferation and Boyden chamber assays identified that stable *SLC14A1*- or *SLC14A1-NLS*-overexpression in J82 and/or UMUC3 cells induced $G_1$ cell cycle arrest and decreased cell percentages in the S phase, suppressed cell viability, proliferation, migration and invasion. **(E)** HUVECs (7,000 cells in 25 μL medium) were treated with conditioned medium (25 μL) from *SLC14A1*- or *SLC14A1-NLS*-overexpressed J82 and UMUC3 cells, respectively, and total branching lengths of tube formation were next examined using the ImageJ software. Relative tubular lengths of HUVECs (%) were decreased after treatment with the conditioned medium for 6 h. **(F)** Similar to that of *SLC14A1*, *SLC14A1-NLS*-overexpression also decreased glucose uptake in J82 and UMUC3 cells. **(G)** A plasmid containing a mitochondrial targeting sequence fused to a red fluorescence tag (pLV-MitoDsRed) was used to

generate replication-incompetent lentivirus as previously described. Transduction of the lentiviral particles containing MitoDsRed showed the morphology and localization of each mitochondrion within a single cell. Immunocytofluorescence with confocal microscopy indicated that either *SLC14A1*- or *SLC14A1-NLS*-overexpression in J82 cells enhanced mitochondrial fusion. The statuses of mitochondrial networks were indicated by arrows. **(H, I, J)** Immunoblot analysis showed that similar to the effects of *SLC14A1*-overexpression, *SLC14A1-NLS*-overexpression downregulated TYMS, DHFR, SLC2A1, HK2, PKM, LDHA/C, pMTOR(S2448), pRPS6(S235) while upregulated PDHA1 and MFN2 protein levels in J82 and/or UMUC3 cells. **(K)** Immunohistochemistry in xenografts from *SLC14A1*- and *SLC14A1-NLS*-overexpressed UMUC3 cells showed that TYMS and DHFR protein levels were downregulated. All experiments were performed in triplicate and results are expressed as the mean ± SD. For HUVEC tube formation, immunocytofluorescence, immunoblot and immunohistochemistry assays, representative images are shown. ACTB served as the loading control for immunoblot analysis. DAPI staining showed the cell nucleus. Statistical significance: $^*P < 0.05$.

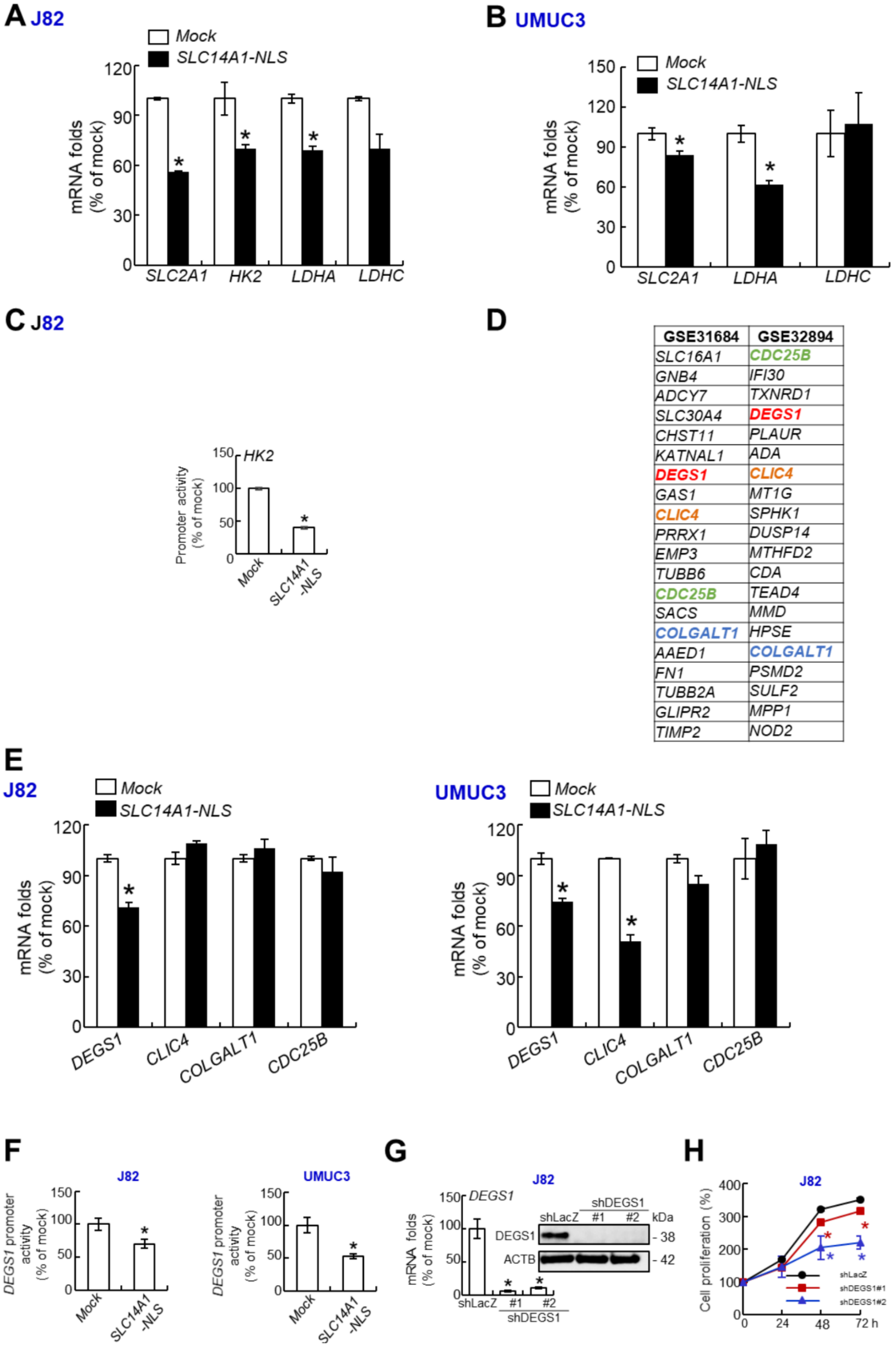


| GSE31684 | GSE32894 |
|---|---|
| SLC16A1 | CDC25B |
| GNB4 | IFI30 |
| ADCY7 | TXNRD1 |
| SLC30A4 | DEGS1 |
| CHST11 | PLAUR |
| KATNAL1 | ADA |
| DEGS1 | CLIC4 |
| GAS1 | MT1G |
| CLIC4 | SPHK1 |
| PRRX1 | DUSP14 |
| EMP3 | MTHFD2 |
| TUBB6 | CDA |
| CDC25B | TEAD4 |
| SACS | MMD |
| COLGALT1 | HPSE |
| AAED1 | COLGALT1 |
| FN1 | PSMD2 |
| TUBB2A | SULF2 |
| GLIPR2 | MPP1 |
| TIMP2 | NOD2 |

**Figure S13.** Nuclear SLC14A1 protein transrepresses the *DEGS1* gene and stable knockdown of the *DEGS1* gene inhibits cell proliferation in UC-derived cells. **(A, B)** Quantitative RT-PCR identified that nuclear *SLC14A1-NLS*-overexpression downregulated *SLC2A1*, *HK2*, *LDHA* and/or *LDHC* mRNA levels in J82 and/or UMUC3 cells. **(C)** Cotransfection of KM2L-phHKII

(RDB05882, Riken BRC) and pGL4.54[luc2/TK] (Promega) plasmids along with Dual-Luciferase® Reporter Assays identified that *HK2* promoter activity was decreased in *SLC14A1-NLS*-overexpressed J82 cells compared to *mock*. **(D)** Data mining on the GEO database (GSE31684 and GSE32894) identified that top 20 transcripts including *DEGS1*, *CLIC4*, *COLGALT1* and *CDC25B* were negatively correlated with the expression level of *SLC14A1* mRNA ($P < 0.05$, data not shown). Four identical transcripts from 2 experiments in the GEO database were highlighted with the same color fonts. **(E**) Quantitative RT-PCR validated that overexpression of the nuclear form *SLC14A1-NLS* gene consistently downregulated *DEGS1* mRNA levels in J82 and UMUC3 cells. **(F)** Cotransfection of one plasmid embracing the *DEGS1* proximal promoter region and its internal control (HPRM45067-LvPG04, Gaussia luciferase, GeneCopoeia) along with Secrete-Pair™ Dual Luminescence Assay (GeneCopoeias) further identified that *SLC14A1-NLS*-overexpression decreased the *DEGS1* promoter activity in both J82 and UMUC3 cells. **(G)** Quantitative RT-PCR and immunoblot analysis showed that stable knockdown of the *DEGS1* gene with 2 distinct shRNA clones in J82 cells downregulated *DEGS1* mRNA and their corresponding protein levels. **(H)** Proliferation assay demonstrated that stable knockdown of the *DEGS1* gene in J82 cells decreased cell proliferation from 0 to 72 h compared to the control (shLacZ). All experiments were performed in triplicate and results are expressed as the mean ± SD. For immunoblot analysis, one representative image is shown and actin, beta (ACTB) served as a loading control. Statistical significance: $*P < 0.05$.

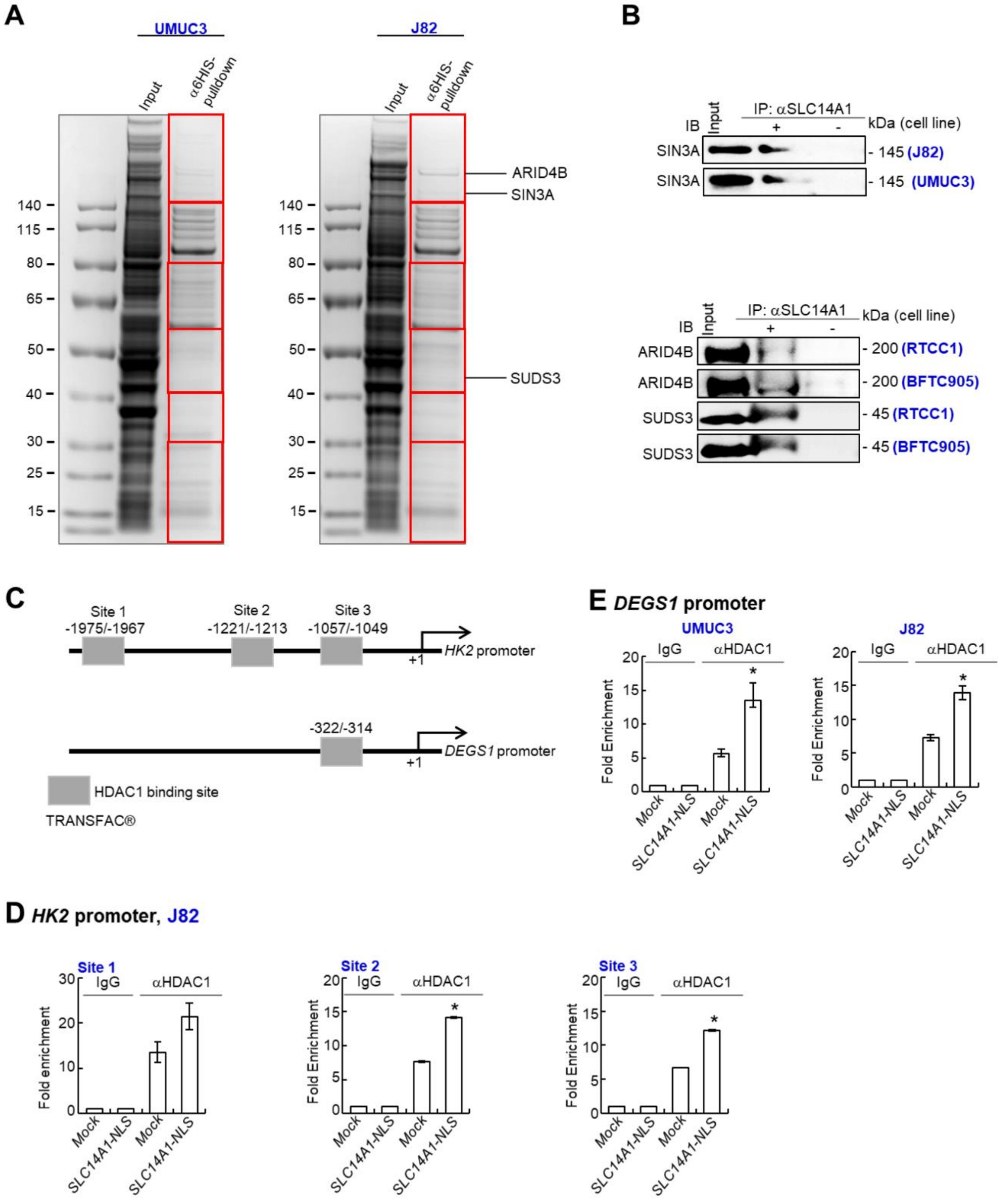


**Figure S14.** SLC14A1 interacts with SIN3A, ARID4B, SUDS3 proteins and nuclear SLC14A1 enhances the interaction between HDAC1 and HDAC1-responsive elements in *HK2* and *DEGS1* promoter regions in UC-derived cells. **(A)** Total protein was extracted from lysates of stable *SLC14A1*-overexpressed J82 and UMUC3 cells, pulled down by probing anti-6HIS antibody (α6HIS pulldown), subjected to SDS-PAGE and specific bands were eluted for mass spectrometry assay. Three potential SLC14A1-interacted proteins, ARID4B, SIN3A and SUDS3 were identified. **(B)** Coimmunoprecipitation (CoIP) assay by probing anti-SLC14A1 antibody and immunoblot (IB) with anti-SIN3A, -ARID4B or -SUDS3 antibody validated that SLC14A1 interacts with SIN3A, ARID4B and SUDS3 in J82, UMUC3, RTCC1 and/or BFTC905 cells. **(C)** TRANSFAC® database predicted 3 and 1 HDAC1-responsive elements in the *HK2* and *DEGS1* promoter region(s), respectively. **(D, E)** Stable overexpression of the nuclear *SLC14A1* (*SLC14A1-NLS*) gene and quantitative chromatin immunoprecipitation (qChIP) assay confirmed that the interaction between

HDAC1 and HDAC1-responsive elements in the *HK2* and *DEGS1* promoter regions in J82 and/or UMUC3 cells. Probing IgG served as negative control. CoIP and qChIP assays were performed in triplicate and results are expressed as the mean ± SD. Statistical significance: *$P < 0.05$.

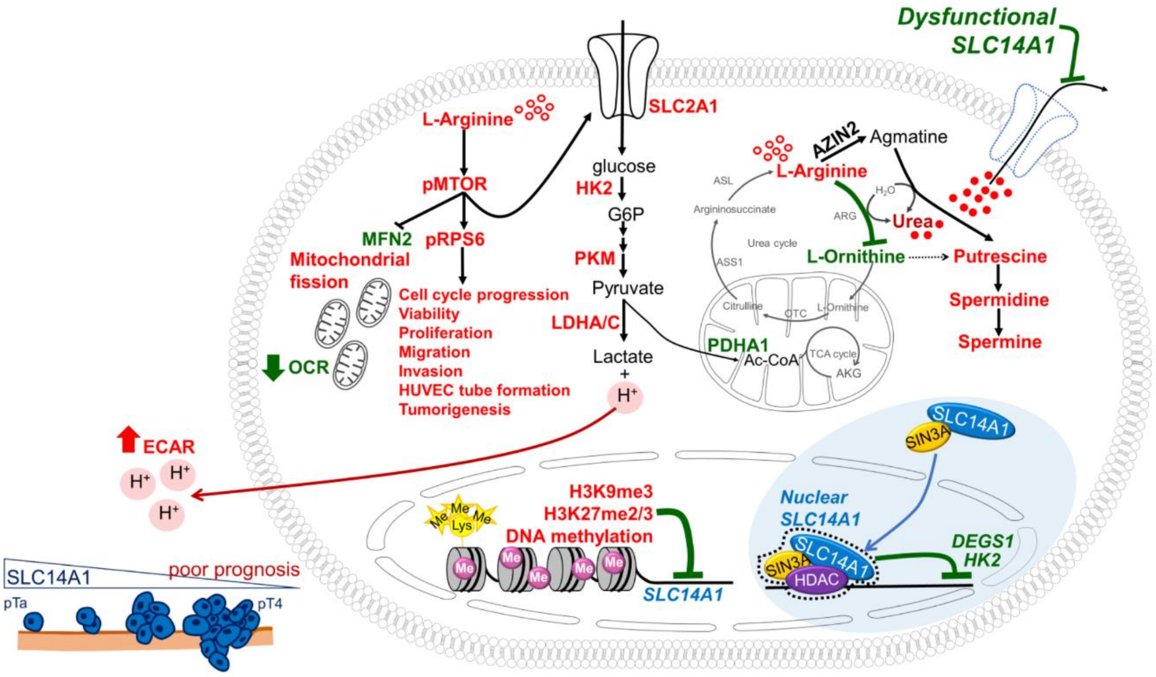


**Figure S15.** Summary of this study. Upregulated and downregulated molecules are indicated with red and green fonts, respectively, by dysfunctional *SLC14A1* gene.
